# Metagenomic insights into communities, functions of endophytes, and their associates with infection by root-knot nematode, *Meloidogyne incognita*, in tomato roots

**DOI:** 10.1038/srep17087

**Published:** 2015-11-25

**Authors:** Bao-Yu Tian, Yi Cao, Ke-Qin Zhang

**Affiliations:** 1 College of Life Science, Fujian Normal University, Fuzhou, Fujian, 350108, China; 2 Key Laboratory for Conservation and Utilization of Bio-resources, and Key Laboratory for Microbial Resources of the Ministry of Education, Yunnan University, Kunming, Yunnan, 650091, China; 3 Key Laboratory of Molecular Genetics, Guizhou Academy of Tobacco Science, Guiyang, Guizhou, 550081, China

## Abstract

Endophytes are known to play important roles in plant’s health and productivity. In this study, we investigated the root microbiome of tomato in association with infection by root knot nematodes. Our objectives were to observe the effects and response of the bacterial endophytes before nematode attacks and to reveal the functional attributes of microbes in plant health and nematode pathogenesis. Community analysis of root-associated microbiomes in healthy and nematode-infected tomatoes indicated that nematode infections were associated with variation and differentiation of the endophyte and rhizosphere bacterial populations in plant roots. The community of the resident endophytes in tomato root was significantly affected by nemato-pathogenesis. Remarkably, some bacterial groups in the nematode feeding structure, the root gall, were specifically enriched, suggesting an association with nematode pathogenesis. Function-based metagenomic analysis indicated that the enriched bacterial populations in root gall harbored abundant genes related to degradation of plant polysaccharides, carbohydrate and protein metabolism, and biological nitrogen fixation. Our data indicated that some of the previously assumed beneficial endophytes or bacterial associates with nematode might be involved in nematode infections of the tomato roots.

Plants harbor a diverse assembly of microbial communities within their tissues. The root microbiomes are termed root endophytes, defined as non-pathogenic microbes residing within plant root tissues^1^. During the past decades, more than 200 genera of endophytic bacteria have been isolated from a large diversity of plants^1^,^2^,^3^. It is widely accepted that endophytes as ubiquitous colonizers of plants play a determining role in the plant’s health and productivity^4^. Recently, the development of culture-independent high-throughput sequencing-based metagenomic analysis has further allowed us to obtain a global view about community structure and diversity of endophytic microbiome residing in plant inner tissues^4^. The best examples are the studies of the bacterial community of root endophytes in *Arabidopsis thaliana*^5^,^6^. In those studies, pyrosequencing of bacterial 16S rRNA gene amplicons was used to define the core endophytic bacterial microbiome in the model plant *Arabidopsis*. The results showed that Actinobacteria and some families from the Proteobacteria were consistently enriched in the endosphere compared with the rhizosphere. Endophytes seemed to be a subset of the host plants’ rhizobacteria, but with a distinct community and composition^5^,^6^,^7^. Sequencing of 16S rRNA gene on root microbiomes of sugarcane and rice also obtained consistent information^8^,^9^. From these work, a wider range of novel endophyte phylogenetic lineages than previously realized was discovered.

Metagenomic analysis of the plant microbiome also explored greater insights beyond the genome information of individual bacterial strains into functional information. Sharing a niche with plant pathogens, endophytes have been thought of as a potential natural source of biological control agents against plant pathogens^2^,^10^. Extensive research has been done to exploit the potential of the plant microbiome as biological control agents. Most endophytic bacteria identified to date have been found to be beneficial for plant health and productivity by suppressing pathogens and promoting plant growth^4^,^7^,^11^. Community genomic and proteomic analyses in plant-associated microbiome demonstrated the roles of endophytic species and their mechanisms in host protection by exploring the genes associated with the production of siderophores, abscisic acid, indole acetic acid, and QS autoinducer. These genes may be associated with biocontrol, plant growth promotion, nutrition, and niche adaptation etc^7^,^8^,^12^,^13^,^14^.

Metagenomic analysis and comparison of plant-associated microbiome have successfully led to a novel phylogenetic and functional insight in the plant microbiome, and their interactions with host plants. However, to date, most community-based analyses on bacterial endophytes have been done with healthy plants. Such studies are insufficient for us to understand what determined whether the endophytes benefitted the host plants, and what shaped the dynamics of the plant-microbiome-pathogen relationship with the absence of pathogens. Further works should be done to investigate the effects and response of the bacterial endophyte on disease attacks in plant.

Root-knot nematodes (*Meloidogyne* spp.), which are among the most damaging sedentary endoparasitic nematodes, often cause severe decline and crop yield losses in agriculture worldwide^15^. Unlike free-living nematodes, root-knot nematodes must feed on a plant host to complete their life cycle. Their eggs hatch into juveniles that infect plant roots and take nutrients from the plant cell. Root-knot nematodes complete most of their life cycle inside the host plant^16^. Here we investigated root microbiome of tomato in association with infection by root knot nematodes to observe bacterial response and functional attributes to the pathogens and plant. This is the first report to observe and define the variation and differentiation of the endophyte and rhizosphere bacterial populations associated with pathogenesis in plant roots based on the 16S rRNA gene-based community analysis and sequencing-based functional metagenomics. Our work will allow us to identify the key bacterial taxa and functions that would benefit or harm plant growth at the community level during infection by plant pathogens.

## Results and Discussion

### General characteristics of the amplicon and metagenomic sequencing

After 55 days of growth, rhizosphere soil samples (HRS1–3 for healthy and IRS1–3 for infected) and root samples (HRC1–3 for healthy and IRC1–3 for infected) were separately collected from the healthy and nematode-infected tomato roots. Approximately 400 bp V3-V4 hypervariable regions of the bacterial 16S rRNA gene were amplified using the genomic DNA extracted from the soil and root samples. The amplicons were sequenced on the Illumina Miseq platform, resulting in 648,339 combined reads with an average length of 411 bp. After initial quality filtering, 186227 quality-filtered reads were obtained, an average of 15523 reads per sample (min = 5,632, max = 27,282). An average of 11272 and 19773 sequences were obtained for rhizospere soil and tomato root samples, respectively (Supplementary Table S1). The difference mainly came from the interference by plant chloroplast DNA, for which about 70% of sequence data was removed as chloroplast sequences from the tomato root sample. The bacterial reads number per sample was rarefied to the smallest number of reads; in this case, 5632 effective bacterial sequences were randomly extracted for the following statistical analysis.

Interference by plant DNA in metagenomics of plant endophytic microbiomes is a common problem in microbial community analysis, and can seriously affect the analysis of community genomics^5^,^17^. In this case, we successfully established a protocol to remove the interference of host DNA (tomato and nematode) in root gall-associated metagenome, and enriched the endophytic microbiome that inhabited the nematode-infected root gall structure. Illumina sequencing for the root gall-associated microbiome resulted in a total of 46,792,278 reads (average length of 100 bp) and about 4.68 Gb of base pairs from a mate-pair library. After preliminary assembly, 21.8 million reads (47.2% of total sequence data) were mapped to the annotated contigs. The majority (85.9%) of the mapped reads were derived from bacteria; only less than 6.3% of reads were mapped to the contigs belonging to nematodes, plants and fungi, suggesting that non-bacterial content was removed in the enrichment and filtration protocols (Supplementary Fig. S4B).

The stringent re-assembly using the extracted bacteria-only sequencing data from the root gall-associated metagenome resulted in 103,448 scaffolds with a total length of 31.27 Mb. Annotation of the gene content predicted 103,851 protein-coding sequences. 82,293 (81.2%) of the predicted protein features could be annotated with similarity to a protein of known function. Of the annotated genes, 67,469 functional features (80.4%) could be assigned into the functional category (Supplementary Fig. S7).

### Community composition and structure of plant-associated microbiome in tomato roots

**Figure 1:**
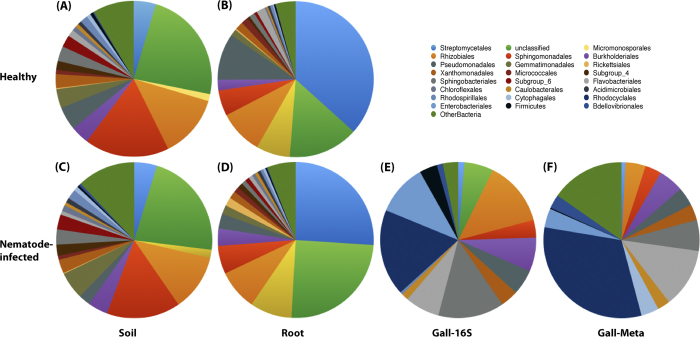
The composition and relative abundance of major bacterial taxa of the root-associated microbiome in healthy and nematode-infected tomato. (**A**) Soil rhizobacteria around the healthy tomato root (HRS); (**B**) Endophytes inhabiting the healthy tomato root (HRC); (**C**) Soil rhizobacteria around the nematode-infected tomato root (IRS); (**D**) Endophytes inhabiting the nematode-infected tomato root (IRC); (**E**) Community composition of the root gall-associated microbiome in the nematode-infected tomato based on 16S rRNA gene-based amplicon (Gall-16S); (**F**) Community composition of the root gall-associated microbiome in the nematode-infected tomato using the taxa-based extracted reads from sequenced shot-gun metagenome (Gall-Meta).

The random subsample of 5362 high quality sequences was clustered into 18921 OTUs (operational taxonomic units) using a 3% distance cutoff, an average of 2666 OTUs per sample (min = 1,767, max = 3,239). The rhizobacteria showed a higher number of OTUs (3048 ± 147), compared to 1442 ± 142 OTUs in the root microbiome (Supplementary Table S3), suggesting a higher OTU richness for plant rhizobacteria than for endophytes. Representative sequences in each OTU were compared with Silva database to assign a taxonomy classification to determine the community composition of the root microbiome. The results demonstrated that the root endophytes and rhizobacteria had significantly different community structures and species abundance (Fig. 1A–D). Actinobacteria (48.67%) and Proteobacteria (32.86%) were the predominant bacterial groups in the tomato root endophytes, followed by Bacteroidetes, TM7, Acidobacteria, and Gemmatimonadetes (Fig. 1B,D). Streptomycetales (36.69%) and Micromonosporales (7.03%) were the predominant groups in Actinobacteria. Members of phylum Proteobacteria were relatively diverse, mainly Rhizobiales (9.15%), Pseudomonadales (9.66%), Sphingomonadales (5.31%), Burkholderiales (2.23%) and Xanthomonadales (1.65%) (Fig. 1A,C). Contrarily, Proteobacteria (52.24%) were the dominant bacterial group in the rhizobacteria, with abundant Sphingomonadales (17.55%) and Rhizobiales (13.09%) of the Alpha-proteobacteria. The results demonstrated that Actinobacteria and Proteobacteria were consistently enriched in the endosphere compared with the rhizosphere, similar to previous observations from plant species such as *Arabidopsis*, rice and sugar cane^5^,^8^,^9^.

**Figure 2:**
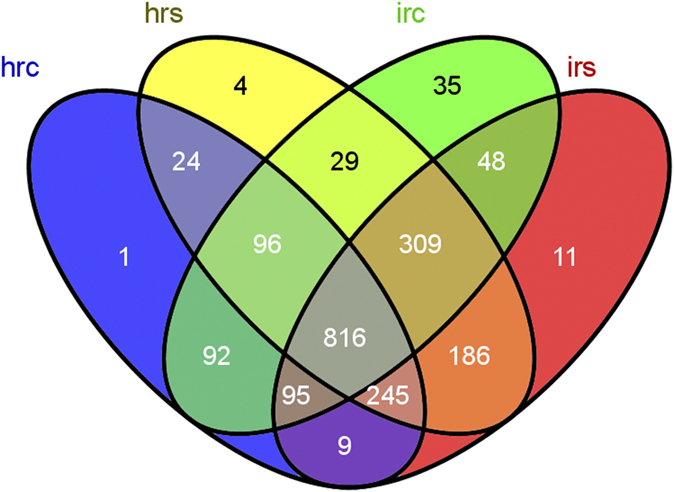
Venn diagram of the tomato root-associated microbiome in healthy and nematode-infected tomato. The observed OTUs for each treatment were produced in the Muthor program and then submitted to VENNY (http://bioinfogp.cnb.csic.es/tools/venny/index.html) to show the shared and unique OTUs. Only the most abundant 2000 OTUs were represented. hrc: healthy tomato root; hrs: healthy rhizosphere soil; irc: nematode-infected tomato root; irs: nematode-infected rhizosphere soil.

The heatmap using relative abundance of the most common 100 defined OTUs in the tomato root endophytes and the rhizobacteria revealed a significant enrichment for the OTUs belonging to Actinomycetales (mainly comprised of Streptomycetales and Micromonosporales) of phylum Actinobacteria, and Pseudomonadales of Gammaproteobacteria. Contrarily, the OTUs belonging to Sphingomonadales and Burkholderiales of Proteobacteria showed a higher relative abundance in the rhizobacteria (Supplementary Fig. S2, Supplementary Table S2). We calculated the statistical differentiation between the root endophytes and the rhizobacteria by a non-parametric T-test metastats. The statistics results confirmed the previous conclusion that the observed community differentiation between tomato root endophytes and rhizobacteria belonged to Streptomycetales, followed by Micromonosporales, Sphingomonadales, Pseudomonadales and Rhizobiales (p < 0.05; Supplementary Table S6). The abundance of Streptomycetales, Micromonosporales and Pseudomonadales was highly enriched in root endophytes, with reduced numbers of Sphingomonadales and Rhizobiales in the rhizobacteria. Further examination of the most abundant 2000 OTUs of the tomato root-associated microbiome in this case revealed that the observed community variation between root and soil samples mainly reflected the quantity of the OUTs, but not species. The majority of the observed OTUs (more than 85.7% in the healthy and 73.76% in the nematode-infected tomato root for the most abundant 2000 OTUs) identified as root endophytes were also identified in the rhizobacteria (Fig. 2). Almost all the most abundant 200 OTUs of the endophytes were also identified in the rhizobacteria around the root, consistent with previous results, suggesting that the endophyte microbiome was derived from the rhizobacteria^4^,^5^.

**Figure 3:**
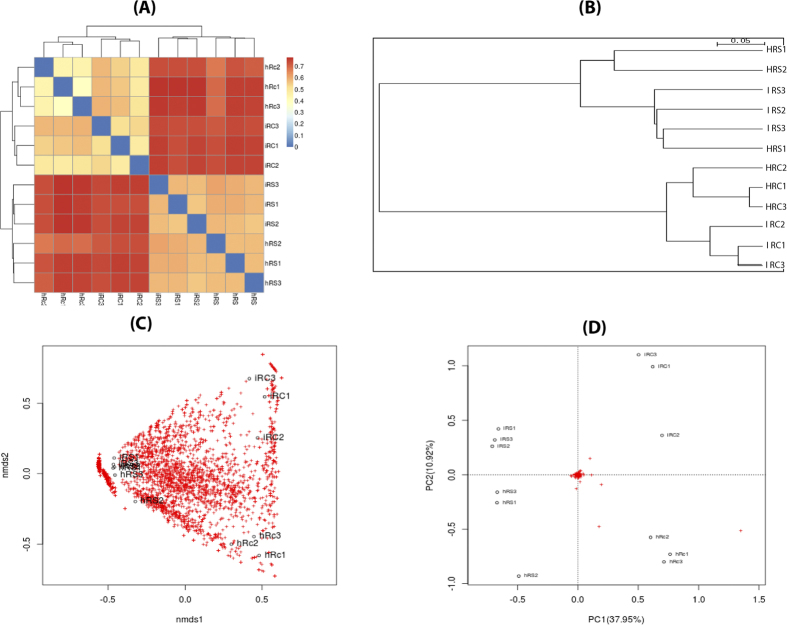
Beta diversity analysis to estimate the dissimilarity and similarity of bacterial community composition among different samples. (**A**) Heatmap derived from dissimilarity matrix of Bray-Curtis distance between bacterial community compositions. (**B**) Thetayc-based cluster analysis of bacterial community composition among the different samples. (**C**) Ordination plot derived from principal coordinated analysis showing the distance between samples or treatments. (**D**) NMDS (nonmetric multidimensional scaling) analysis showed the dispersion of OUTs in each sample. HRC1-3: healthy tomato root; HRS1-3: healthy tomato rhizosphere soil; IRC1-3: nematode-infected tomato root; IRS1-3: nematode-infected tomato rhizosphere soil.

Alpha diversity in soil rhizobacteria and root endophytes was calculated by using the Chao estimator, Shannon index and Simpson’s diversity index, all of which demonstrated a higher bacterial richness and diversity in soil rhizobacteria than those for endophytes, both in healthy and nematode-infected tomato samples (Shannon index: soil = 7.36, root = 5.83, p = 0.0002 < 0.001; HRS = 7.25, HRC = 5.38, p = 0.0006 < 0.001; IRC = 6.38, IRS = 7.44, p = 0.0059 < 0.05; Supplementary Table S3; Supplementary Table S4). Beta diversity analysis of the tomato root-associated microbiome showed that variability in the root microbiome was quite different from rhizobacteria, and the differentiation between them was far higher than that between the healthy and nematode-infected roots (Fig. 3). As shown in the thetayc-based cluster analysis, the root endophytes harbored communities that clustered separately from the root rhizobacteria. In both analyses, the samples in healthy and nematode-infected treatment grouped together, respectively (Fig. 3B). NMDS plot analysis showed differentiation among different OTUs in the root microbiome was higher than that in the soil samples (Fig. 3C,D).

### The effects of nematode infection on tomato root endophytes and rhizobacteria

The infection by nematode resulted in variation and differentiation of the endophyte and rhizosphere bacterial populations when compared to those from healthy plant roots. However, the variation caused by nematode infection in the root endophytes was more significant than that in the rhizobacteria (Supplementary Table S3). Alpha diversity of root endophytes estimated by the Shannon index showed a small but significant variation between healthy and diseased tomato roots, but the differentiation in the rhizobacteria around roots was not statistically significant (HRC = 5.38, IRC = 6.38, p = 0.0155 < 0.05; HRS = 7.25, IRS = 7.44, p = 0.209; Supplementary Table S4). The endophytes in the nematode-infected roots had a higher OTU richness and diversity than did the endophytes in healthy plants (Supplementary Table S3). Similar results could be observed from the VENN diagram in the statistics of shared OTUs between different treatments (Fig. 2). Additional 94 OTUs (35 OTUs specific for IRC and 11 OTUs specific for IRS) were found in the microbiome associated with the nematode-infected tomato root, suggesting that other microbial species entered the tomato roots during nematode infection. Consistent with Alpha diversity analysis, Beta diversity analysis between different samples showed the striking effect of nematode infection on the microbiome both for root endophytes and rhizobacteria (Fig. 3B,C). The root endophytes associated with nematode infection showed a greater dispersion of observed OTUs, suggesting that additional bacterial content was brought into the root in conjunction with nematode infection.

Endophytes in the nematode-infected root were dominated by Streptomycetales (26.02%) and Micromonosporales (8.70%) of Actinobacteria, followed by Rhizobiales (8.39%), Sphingomonadales (5.82%), Burkholdales (3.61%), and Pseudomonadales (3.05%) of Proteobacteria. Compared to the community composition in the healthy root, a significantly reduced richness was found in Streptomycetales and Pseudomonadales, with a slight increase in Burkholdales and Micromonosporales among the diseased root endophytes (Fig. 1). The results from metastats analysis (p < 0.05) supported the observation that the bacterial community affected by nematode infection was characterized primarily by the OTUs belonging to Actinomycetales and Pseudomonadales (Supplementary Table S6). In the soil rhizobacteria around the nematode-infected roots, the OTUs belonging to Sphingomonadales of Alphaproteobacteria contributed primarily to the variation compared to the healthy soil sample (Supplementary Table S6).

To obtain direct evidence of the root gall-inhabiting microbiome associated with nematode infection, we determined community profile of the microbiome specifically inhabited in root knots, plant gall structures formed by *Meloidogyne* infection in tomato roots. The tomato root galls were collected carefully, mashed and filtered to obtain the enriched gall-associated endophytes (Supplementary Fig. S1). Remarkably, Proteobacteria and Bacteroidetes absolutely dominated the bacterial content of endophytes in the root galls, accounting for approximately 92.7% of the total root gall-associated microbiome. Compared to the endophytes from healthy or diseased roots, the gall-associated microbiome demonstrated a distinct structure composition and relative abundance of microbial communities, in which Rhodocyclales (17.93%), Sphingobacteriales (13.67%), Rhizobiales (13.50%), Enterobacteriales (10.65%), Flavobacteriales (7.18%) and Burkholderiales (7.01%) were the dominant bacterial groups at the order level. Only less than 2% of the endophytes were identified as Actinobacteria (Fig. 1E). In the subsequent metagenomic sequencing for the root gall-associated microbiome, percentage of extracted reads that mapped to different bacterial groups from a sequenced shotgun library confirmed composition of root gall-associated endophytes in the 16S rRNA gene-based community analysis (Fig. 1F).

The reduced abundance of Actinobacteria may be explained by the effect of the enrichment process; for example, the hypha-forming microbes might have been filtered out with the fungi and plant tissue by the series of filters. However, some important information regarding the process of nematode infection can be gleaned from the variation and community composition of the root gall-specific endophytes. One of the most striking community variations in the microbiome associated with *M. incognita-*formed root knots was that one sub-community, including Rhodocyclales, Enterobacteriales, Sphingobacteriales and Flavobacteriales, was specifically and highly enriched. These bacteria were identified in a very small fraction (less than 1%) or in the whole root, but a significantly increased fraction in the root gall-specific microbiome (from 7% to 18%), suggested their involvement or association with the infection activity of *M. incognita* in plant root knots. Moreover, few OUTs of Rhodocyclales and Enterobacteriales were identified in the whole healthy and whole nematode-infected tomato roots. A second differentiated sub-community, which predominated among the healthy root endophytes but showed dramatically reduced richness in root galls, was Pseudomonadales and Actinomycetales. Pseudomonadales of Gammaproteobacteria, a predominant bacterial group which was significantly enriched from 4.87% in the rhizobacteria to approximately 10% of total identified OTUs among the endophytes, decreased to about 3% among the root gall-associated endophytes. Actinobacteria (mainly comprised of Streptomycetales and Micromonosporales) was the predominant bacterial group, which also showed a significant enrichment among the healthy root endophytes: approximately 48.76% of total identified OTUs among the endophytes compared to 9.43% among the rhizobacteria. However, a significantly decreased overall proportion (39.82%) of Actinobacteria in the nematode-infected root endophytes was detected compared to the healthy endophytes. Closer examination at the lower taxonomic levels indicated that the variation in the diseased roots was derived from a combination of various members of Actinobacteria, a remarkably decreased OTU richness in Streptomycetales from 36.69% to 26.02%, and an increased abundance of members of Micromonosporales from 1.46% to 8.70%. The paired metastatistical analysis results also confirmed the observation that the differentiation of root endophytes between the healthy and diseased tomatoes mostly came from the variation in members of Actinobacteria. So, although the low fraction of Streptomycetales (1.28%) in the root gall-associated microbiome might have been affected by the filtering process, the decline of species richness of Streptomycetales in the nematode-infected plant roots was still significant and was even lower in the nematode root galls. In addition, Rhizobiales, which was ubiquitously identified among tomato roots and rhizosphere bacteria with a consistent community structure in the healthy and nematode-infected root endophytes, showed a reduced richness in the root gall microbiome. Moreover, the variation of Rhizobiales in the gall-associated microbiome resulted from a change in the dominant bacterial species. In tomato root and rhizosphere bacteria, most of the Rhizobiales bacteria belonged to a diverse assembly of symbiotic nitrogen-fixing bacteria, such as *Rhizobium*, *Devosia* and *Bradyrhizobium*. However, the predominant bacterial group of Rhizobiales in root galls was the genus *Agrobacterium*, which usually acts as a plant pathogen.

Comparison of community composition and OTU richness of the root-associated microbiome between healthy and nematode-infected tomatoes indicated that the disease attack had reassembled community structure of the microbiome in root endophytes, especially in the nematode infection sites, tomato root gall (Supplementary Table S4). Although this variation provides no information regarding cause and effect on the pathogen, the observed community differentiation in the root endophytes and rhizobacteria permits some speculation. In most cases, Actinomycetales were known to produce a vast diversity of active compounds against microbial pathogens^18^,^19^ and nematode pests or eggs^20^,^21^,^22^,^23^. The community differentiation between the healthy and infected plant roots of these bacteria, which were previously assumed to be beneficial to plants, indicated a response by the biocontrol microbes to the infection by the pathogen in the plants. A similar result was observed in Pseudomonadales, the second-most abundant bacterial group in the healthy tomato root. Pseudomonadales was also widely studied for its diverse ability to promote plant growth, suppress nematodes and microbial pathogens, and induce systemic resistance in plants^20^. The overall decrease in community composition and abundance of the antagonistic endophytes in nematode-infected tomato plant roots suggested a cause and effect response, of the beneficial microbes in tomato root tissue upon an attacks by nematode pathogen.

Furthermore, Rhodocyclales, Sphingobacteriales, Enterobacteriales and Flavobacteriales were the main nematode-derived sub-community in the root gall-associated microbiome. OTUs belonging to these bacteria were specifically and highly enriched in root knots formed by nematodes, suggesting that these bacterial groups might be associated with the infection by root-knot nematodes in the plant roots. Among them, Sphingobacteriales and Flavobacteriales were among the dominant bacteria in plant rhizobacteria or endophytes. However, for Rhodocyclales and Enterobacteriales, few or none of the OTUs of these members were detected in tomato rhizobacteria or endophytes, whether in the healthy or nematode-infected roots. A previous study suggested that genera or species of Enterobacteriales were commonly attached to the cuticle of the root knot nematode as part of their lifestyle in suppressive soil or bacterial associates^23^,^24^, suggesting that some members of the root gall-associated endophytes, such as Rhodocyclales, Enterobacteriales or the plant pathogen *Agrobacterium*, may have entered into the root’s inner tissue as bacterial associates of a nematode infection.

### Plant cell-wall degrading enzymes served as the determinant for the colonization by endophytes or as a pathogenic factor

Endophytes were thought to have stemmed from the rhizosphere microbiome or a sub-population of the rhizobacteria^4^. Comparative analysis of community profiles and the OUT richness of the microbiomes in the tomato rhizosphere and root confirmed the results. The observed OTUs in the tomato root endophytes were also identified in the rhizobacteria around the tomato root. The latter showed higher diversity and OTU richness (Supplementary Table S3). The results were consistent with community analysis of the microbiomes associated with other plants, such as rice, *Arabidopsis* and sugar cane. These plants harbored a similar endophytic profile, with Actinobacteria and Proteobacteria as the dominant groups in the endophytic microbiome^7^,^8^,^9^. Although the endophytes were part of the rhizobacteria, not all the rhizobacteria were endophytes. Bulgarelli *et al.*^5^ proposed that lignocellulose nature of the plant hosts played a more important role than the plants’ internal environment in determining whether rhizobacteria were endophytes.

The plant cell wall, predominantly composed of lignocellulose, serves as the main barrier to protect the plant from invasion by foreign organisms. Phytopathogens secrete numerous cell wall-degrading enzymes, such as cellulase, xylanase, pectin lyase etc., to break through the plant cell wall to attack the plant^25^. Solomon and Matthews^26^ suggested that the plant determines the entry of bacteria into host root tissue to become endophytes. That is, like microbial pathogens, the endophytic bacteria also need find a way to break through the plant lignocellulose barrier to enter into the plant tissue. The difference is that endophytes cause no negative symptoms in the host. The evidence has demonstrated that the colonization of endophytes in plant internal tissues involved the production of cellulases and pectinases such as endoglucanase, pectate lyase and polygalacturonase^3^,^27^, indicating that cell wall degrading enzymes were most likely a key determinant for the bacteria to initially enter and colonize the plant host, and the native endophytic bacteria had the ability to secrete diverse enzymes to penetrate the polysaccharide barrier enabling them to reside within the plant.

To obtain an overall insight into the genetic potential for production of lignocellulose-degrading enzymes by root endophytes, we examined the assembly of sequenced root gall-associated metagenomic data for the presence of gene sequences coding for lignocellulose-degrading enzymes. Using the CAZymes database (Carbohydrate degrading enzyme) and KEGG (Kyoto Encyclopedia of Genes and Genomes) pathway Database, we identified within the microbiome a number of genes encoding enzymes that were involved in degrading polysaccharides of plant cell walls (Supplementary Table S7; Table 1). Among these, the largest proportions of the identified features encoding CAZymes were related to the oligosaccharide-degrading enzymes. Only lower proportion of genes encoding cellulases (endo-1,4-β-D-glucanase), hemicellulases/debranching enzymes, or pectate lyases, which mainly act on the backbones of full-length polymers of cellulose, xylan, and pectin, were found (Supplementary Table S7; Table 1). Genes encoding endo-β-1,4-xylanase, pectin lyase, polygalacturonase and lignin-degrading enzymes were not detected. High abundance of the genes encoding CAZymes associated with oligosaccharide degradation or simple sugar utilization, and low number or absence of the genes that encoded enzymes mainly acted on the backbone chain polysaccharides, supported the previous hypothesis that the endophytic bacteria only partially degraded inner plant polysaccharides and utilized the oligosaccharides as nutrients, but did not significantly damage the plant tissues or cause negative symptoms as would phytopathogens^25^,^26^,^27^.

The identified features encoding enzymes involved in the degradation of polymers of xylan, oligosaccharide-degrading enzymes or xylan debranching enzymes were widely distributed across the main bacterial groups of endophytic Sphingobacteriales, Rhizobiales, Pseudomonadales, Flavobacteriales, Enterobacteriales and Bacteroidales. The genes encoding cellulases were identified from the Sphingobacteriales, Rhizobiales, Cytophagales, Enterobacteriales and Burkholderiales assembly. In addition, it was previously reported that Actinomycetales showed the ability to secrete a number of oligosaccharide-degrading enzymes^28^. It was concluded that the main native endophytic groups of the examined tomato root microbiome, Actinomycetales, Sphingobacteriales, Rhizobiales, Pseudomonadales, Flavobacteriales, Bacteroidales, Xanthomonadales, Cytophagales and Caulobacterales, all harbored genes encoding cell wall degrading enzymes with a limited ability to partially degrade plant lignocellulose material. These enzymes might be the key determinants for bacteria to penetrate the polysaccharide barrier to reside inside the plant tissues as plant root endophytes. Contrarily, for some dominant bacterial groups of rhizobacteria, such as Sphingomonadales, only a few oligosaccharide-degrading enzymes but neither cellulase nor xylanase were found. This may explain the low proportion of this bacterium among root endophytes compared to its high proportion among the rhizobacteria.

In gall-specific endophytic bacteria, Sphingobacteriales, Flavobacteriales and Enterobacteriales harbored the most abundant plant cell wall degrading enzyme genes, accounting for more than half of them identified in the root gall microbiome. Moreover, the largest numbers of cellulase genes (12 of 15) in the root microbiome were identified in the root gall endophytic Sphingobacteriales, Enterobacteriales and *Agrobacterium* of Rhizobiales. Specifically enriched lignocellulose-degrading endophytes in tomato root galls suggested the plant cell wall degrading enzymes might be also involved in the infection process of root-knot nematodes.

It has been proposed that wood-feeding insects harbor cellulose-degrading bacteria to help the insect host to penetrate the plant barrier and contribute to host nutrition^28^. In plant-parasitic nematodes, recent studies revealed that several families of cell wall-degrading enzymes, including xylanases, cellulases and pectate lyases, were also involved in the nematode infection process during the invasion and intracellular migration in plant inner tissue^29^. Danchin *et al.*^29^ identified a diverse suite of proteins capable of degrading plant cell walls from the sequenced genome of *Meloidogyne hapla*. Cyst nematodes were also shown to produce a variety of polysaccharide-degrading enzymes during their migration through the root^30^,^31^. However, these studies suggested that the cell wall-degrading enzymes secreted by cyst nematodes were not involved in the development of nematode feeding sites, which required the fusion of root cell protoplasts by partial cell wall dissolution^30^. Despite different ontogeny, functions and cellular features of nematode feeding sites (NFS) of root knot nematode were similar with those of cyst nematode. Formation of both types of feeding sites required the destructive and constructive modification of plant cell walls. There are no reports yet indicating that enzymes secreted by plant-parasitic nematodes are involved in the process^30^,^32^,^33^. Specifically enriched cellulose-degrading bacteria in nematode feeding sites raised the hypothesis that the root gall-enriched bacteria, derived from nematodes, might reinforce the enzyme profiles to dissolve plant cell walls of the giant cells and neighbor cells in plant host for the formation of nematode feeding sites.

In addition, nematode feeding sites were the sole source of nutrients for the developing plant-parasitic nematodes within the root tissue. The nematodes absorbed nutrients from the enlarged plant cells for their subsequent growth and reproduction^33^. Previous studies have detected high metabolic activity inside nematode feeding sites, such as elevated sucrose levels, accumulation of starch and specific sugars, and elevated levels of many amino acids. The identification of numerous oligosaccharide-degrading enzymes in the root gall-enriched bacteria provides an explanation of metabolic activities in the giant cells. Unlike the enzymes produced by the nematode or the plant host, the vast majority of the CAZymes identified in Sphingobacteriales, Flavobacteriales and Enterobacteriales were mainly involved in oligosaccharide degradation or simple sugar utilization, suggesting that these bacteria might be involved in carbohydrate metabolism to compensate for changing carbohydrate solute uptake by the nematode at nematode feeding sites (Supplementary Table S7; Table 1).

### Specific enrichment of Rhodocyclales as biological nitrogen-fixers in root gall-associated microbiome

Rhodocyclales, specifically identified in root galls, was the most abundant bacterial group in the root gall-associated microbiome. The predominant species of Rhodocyclales among the tomato root gall microbiome was identified as *Azozpira suillum* PS, formerly called *Dechlorosoma suillum* PS. Members of the genus had been isolated as endophytes from Kallar grass and rice, however, in most cases, the strains of *Azozpira* were identified as free-living bacteria, for example, from groundwater and waste plants^34^,^35^. In microbial community analysis, few OTUs of Rhodocyclales were identified among tomato root rhizobacteria or endophytes. Contrarily, Rhodocyclales were highly enriched in the tomato root knots. Analyses of CAZymes showed that few lignocellulose-degrading enzymes were detected in the Rhodocyclales assembly, suggesting that the microbes might enter the root gall with invading nematodes.

**Figure 4:**
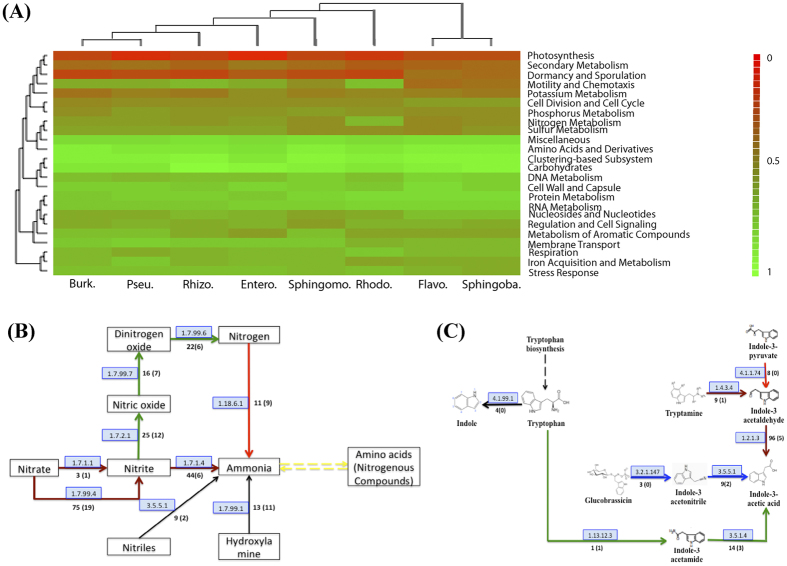
Functional annotation and categories of different taxa-based assemblies in the tomato root gall-associated microbiome. (**A**) Heatmap depicting the relative abundance of functional categories of the major taxa-based assemblies in the tomato root gall-associated microbiome. Burk.: Burkholdales; Pseu.: Pseudomonadales; Rhizo.: Rhizobiales; Entero.: Enterobacteriales; Sphingomo.: Sphingomonadales; Rhodo.: Rhodocyclales; Flavo.: Flavobacteriales; Sphingoba.: Sphingobacteriales. (**B**) Pathway involved in nitrogen metabolism in the tomato root gall-associated microbiome. Arrows indicate nitrogen fixation in red, denitrification in green, nitrate and nitrite assimilation in brown. Numbers inside boxes with light blue background represent EC numbers of the enzymatic reaction. The numbers opposite boxes along the arrows represent the detected proteins in the metagenome (Rhodocyclales). (**C**) Pathway involved in the biosynthesis of IAA (indole-3-acetic acid) in the tomato root gall-associated microbiome. Arrows indicate IPyA pathway in red, the IAM pathway in green, the IAN pathway in blue, and the TAM pathway in brown. Numbers inside boxes with light blue background represent EC numbers of the enzymatic reaction. The numbers opposite boxes along the arrows represent the detected proteins in the metagenome (Pseudomonadales).

The species of genus *Azozpira*, which had shown their ability to grow in nitrogen-free growth medium, were characterized as nitrogen-fixing bacteria^34^. Gene-based evidence for a nearly complete nitrogen metabolism pathway was present in the Rhodocyclales assembly of the root gall metagenome (Fig. 4B; Supplementary Table S8). The genomic analysis for the Rhodocyclales assembly indicated that Rhodocyclales contained the genes encoding a nitrogenase complex, which can convert atmospheric nitrogen into ammonium. The nitrogenase complex in Rhodocyclales was comprised of two components, MoFe protein NifDK and Fe protein NifH, which clustered with *nifA* and *nifB* genes (Supplementary Table S8)^36^. Additionally, the abundant gene contents related to the pathways of denitrification and nitrate and nitrite ammonification, which assimilate nitrate and nitrite to ammonium, or denitrificate to dinitrogen oxide or even nitrogen, were also represented in the Rhodocyclales assembly^37^. The fixed ammonium could be assimilated by the glutamine synthetase pathway, through transformation of nitrogenous compounds to synthesize alpha-amino acids (Fig. 4B; Supplementary Table S8). Specific enrichment of the nitrogen-fixing bacteria in the root gall suggested that Rhodocyclales might be an important provider of nitrogen for the development and reproduction of root knot nematode or the host plant.

It is widely known that bacterial symbionts are involved in providing host insects with essential nutrients not found in their diets^38^,^39^. An example is phloem-sap aphids, which acquire nitrogen-poor nutrients from plant phloemsap^39^. As for other insect-host plant interactions, nitrogen is the most limiting nutrient to root-knot nematodes and their plant hosts, which could become even more limiting after root damage caused by infection as the galled roots have a reduced ability to absorb and transport water and nutrients from the soil^40^. However, metabolic profiling analysis showed increased metabolic activities related to many amino acids and carbohydrates in nematode feeding sites, suggesting that the plant-parasitic nematodes had other ways of acquiring nutrients^33^. Metagenomic analysis of the nematode knot-associated microbiome demonstrated that, high abundance of gene contents in nitrogen metabolism, including biological nitrogen fixation capacity in Rhodocyclales, and also the ammonification and ammonia assimilation pathways in other root gall-associated endophytes Sphingobacteriales and Flavobacteriales (Supplementary Table S8). The result suggested that the microbes inhabiting root galls might build a mutualistic relationship with their nematode hosts by providing necessary nutrients for the giant cells of the plant root to supplement nitrogen uptake for the developing nematodes. The well-established mutualistic relationship between endophytes and plants has been demonstrated in a previous study that endophyte transferred fixed nitrogen to the plant host^41^. Although the underlying mechanism for the nutrient delivery system between the gall-specific endophytes and the plant or nematode is not fully understood, the transport proteins in plasmalemme, such as the transporter for amino acids and nitrogen source, likely play an active role in nutrient uptake in symplastically isolated giant cells at nematode feeding sites^33^.

In fact, the effect of nitrogen sources on root-knot nematode parasitism in plant roots has been investigated for a long time. It has been accepted for decades that the utilization of organic amendments in soils can suppress plant-parasitic nematodes and reduce nematode invasion through release of ammonia^42^. However, the underlying mechanism about how the nitrogen sources affected the root-knot nematode infection of plant roots remained unknown. Linford *et al.*^43^ believed that the addition of organic matter stimulated nematode-trapping fungi to kill nematodes. A recent study revealed that urea produced by the bacteria from cow dung was catabolized to ammonia, which served as a signal molecule in nematode-trapping fungi to induce the formation of traps to kill free-living nematodes^44^. Another study was conducted to compare the effects of different nitrogen forms on root-knot nematodes. The result attributed the reduction of nematode populations to the attraction or repulsion effects of different forms of nitrogen^45^. However, both experiments were executed under laboratory conditions, and the effects of nitrogen might be interfered in practice by the microbial nitrogen metabolism in the soil (nitrification and denitrification), and lack of nematode-trapping fungi among root endophytes in the case of root knot nematode. The biological nitrogen fixation process is a tightly regulated process, affected by the available nitrogen sources in the soil^36^. Here we hypothesize that, organic soil amendments, ammonia, and high concentrations of inorganic nitrogen sources in the soil might repress the biological nitrogen fixation capacity of the nitrogen-fixing bacteria associated with nematode root gall and result in a reduced supply of nutrients which can further suppress the development of nematodes.

### Plant growth promotion endophytes and their benefits to plants

In the past decades, plant endophytic bacteria attracted extensive attention mainly due to their beneficial effects on plant health and productivity, such as plant-growth promotion, disease suppression, and biological nitrogen fixation etc^1^. Metagenomic analysis for the root gall-associated microbiome also revealed that endophytes harbored a number of plant-beneficial traits by either providing necessary nutrients or producing phytohormones, or indirectly by suppressing various pathogens as biocontrol bacteria.

It has been widely accepted that the soil or plant-associated microorganisms could produce IAA to promote plant growth^7^,^36^,^46^. Four pathways involving biosynthesis of IAA (indole-3-acetic acid) using the various intermediates from the precursor tryptophan were detected in the tomato gall root metagenome: the indole-3-pyruvic acid (IPyA) pathway, the indole-3-acetamide (IAM) pathway, the indole-3-acetonitrile (IAN) pathway and the tryptamine (TAM) pathway (Fig. 4C; Supplementary Table S9). Almost all the predominant bacterial groups in tomato root endophytes harbored the IAA biosynthesis pathways, consistent with previous studies that the majority of the bacterial isolates from plant rhizobacteria and endophytes were able to synthesize IAA, suggesting that plant growth might be promoted by the root endophytes^36^,^46^. However, among tomato root endophytes, only Pseudomonadales harbored an intact biosynthesis route for IAA production from the tryptophan precursor through the indole-3-acetonitrile pathway. In other tomato root endophytes, the pathways of IAA synthesis were incomplete, with the absence of genes responsible for biotransformation from tryptophan to the intermediates indole-3-pyruvic acid, indole-3-acetamide and tryptamine. Observation of incomplete IAA biosynthesis pathways in root endophytes suggested that, if the bacteria could not synthesis the intermediates themselves, they might produce auxins using intermediates from the plant. In this sense, the assumed plant growth promotion in the rhizobacteria or endophytes with IAA biosynthesis pathways might not be surprising. The IAA production in these bacteria might disturb the biosynthesis pathways and regulation processes in the host plant, thereby influencing either tumor formation or normal plant growth and development. As was the case in plant pathogenic bacteria, IAA production was also widely identified, and is thought to be involved in plant tumor formation^7^.

Metagenomic analysis of tomato root endophytes also revealed numerous beneficial features that could protect plants from phytopathogens, providing candidate biological control agents. The genes responsible for several mechanisms were identified, such as production of antagonistic compounds including antibiotics, siderophores, and chitinases to suppress microbial and nematode pathogens (Table 1; Supplementary Table S10).

### Genes with functions specific to different bacteria

To provide an indication of the enriched functions contributing to the prevalence of a specific bacterial group among the root endophytes, we compared the functional categories based on a subsystem for the dominant bacterial groups (Fig. 4A; Supplementary Table S11). The identified dominant bacteria in root gall-associated microbiome demonstrated higher relative abundance for the genes associated with carbohydrate metabolisms, clustering-based subsystems and amino acids and derivatives metabolisms, suggesting high rates of metabolic activity related to carbohydrate and amino acid metabolism in the root gall-specific endophytes. Sphingobacteriales and Flavobacteriales were clustered together apart from other endophytic groups, showing their close functional similarity (Fig. 4A). We found significant functional enrichment related to the specifically enriched endophytes: carbohydrate (glycoside hydrolases and central carbohydrate metabolism) and protein metabolism (protein biosynthesis) in Sphingobacteriales, and nitrogen metabolism in Rhodocyclales, supporting the previous discussion about the putative functional contribution of gall-specific endophytes to the infection by and development of nematode pathogens in tomato root knots formed by nematode infection. Gene functional enrichment analysis also showed higher relative abundance of pathogenicity-related genes, such as regulation and cell signaling (quorum sensing and biofilm formation) and membrane transport (protein secretion system, Type VI) in Rhizobiales (*Agrobacterium*), Burkholderiales, Flavobacteriales, Enterobacteriales and Rhodocyclales, all which were defined as specifically enriched bacterial groups in root knots, suggested these bacteria might serve as the pathogenic complex of nematode pathogens in the infection on host plant.

## Concluding Remarks

In this study, we investigated root-associated microbiome in healthy and nematode-infected tomatoes, to observe the responses of bacterial communities during nematode pathogenesis and to reveal their functional attributes in microbe-plant-nematode interactions. Comparative community analysis of rhizospheric and endophytic bacteria in healthy and nematode-infected tomato roots showed that nematode pathogenesis resulted in a decreased abundance of the predominant endophytic groups Streptomycetaceae and Pseudomonadales, both of which were known to produce a vast diversity of active compounds against plant pathogens. Further analysis of the root gall-associated microbiome in nematode-infected tomatoes found the enrichment of the endophytic groups Sphingobacteriales, Flavobacteriales, and nematode-associated bacteria Rhodocyclales and Enterobacteriales. The results indicated that, infection by root-knot nematode resulted in reassembled microbial communities of the root microbiome in diseased tomato host, especially in the specialized root gall formed by nematode infection. Metagenomic analysis for the root gall-associated microbiome revealed that the nematode associated bacterial groups seemed to be involved in several key infection processes during nematode pathogenesis in tomato root, including reinforcing plant cell walls destruction at the nematode feeding sites, or allowing a mutualistic relationship involving provision of nutrients. Further identification and investigation of the root gall-associated bacterial group and their functional attributes in nematode pathogenesis in the future will help us better understand the complex microbe-plant-pathogen interaction and improve agricultural practice by developing new strategy to control plant-parasitic nematodes.

## Materials and Methods

### Sample collection and DNA extraction

Tomato plants (*Solanum lycopersicum* cv. Jiabao, a tomato cultivar susceptible to *Meloidogyne incognita*) used for the healthy and nematode-infected treatments were separately grown in pots in a greenhouse in Guizhou, China. The healthy and nematode-infected treatments were performed with at least three repetitions. Tomatoes in nematode-infected experiment were inoculated with single egg masses of root-knot nematode (*Meloidogyne incognita* race1) at the fourth true leaf stage. The rhizosphere soil and tomato root samples were separately harvested from the healthy or infected tomatoes 55 days after infection (Supplementary Fig. S1). The surface sterilized root samples were further pulverized according to the previously described method^24^. Metagenomic DNA was separately extracted from the collected rhizosphere soil and pre-pulverized tomato roots including in four treatments: healthy tomato root (HRC), healthy tomato rhizosphere soil (HRS), nematode-infected tomato root (IRC) and nematode-infected tomato rhizosphere soil (IRS), using Power Soil DNA Isolation Kit (MoBio Laboratories, Carlsbad, CA, USA). Finally, 12 soil and root genomic DNA samples, each treatment with three repeated samples, for Illumina Miseq sequencing were obtained.

To obtain direct genetic information of the microbiome associated with nematode pathogenesis, we constructed a shotgun library and performed sequencing-based metagenomic analysis on the microbiomes inhabiting tomato root knots with nematodes (galls) (Supplementary Fig. S1). The 30 of nematode-infected tomato roots were harvested and surface-sterilized. The root galls containing *M. incognita* (with excess root material removed) were carefully collected and soaked for 4 hours at room temperature in a pectinase solution to soften the plant tissue. Softened root galls were placed in a sterile mortar with beads and gently mashed with a pestle to crush galls. The resultant slurry was transferred to a 250 mL glass flask with 50 mL distilled 0.2 M PBS buffer (pH 7.0) and then shaken to disperse the root materials. The homogenized solution was filtered through a series of stacked sieves: 250-μm, a 75-μm, and a 25-μm pore sieve to remove the host plant and nematode material. The pooled solution was then passed through a 10-μm polycarbonate filter (Millipore) under vacuum and the filtrate was recovered and centrifuged at 8,000 rpm to obtain five tubes of endophytic bacteria sediment. Total genomic DNA was extracted from one tube of endophytic bacteria sediment using Power Soil DNA Isolation Kit and used for further metagenomic sequencing analysis.

### PCR amplification of 16S rRNA gene and Pyrosequencing

Hypervariable regions V3-V4 of bacterial 16S rRNA gene were amplified using the primer pair 338F (5′-ACTCCTACGGGAGGCAGCA-3′) and 806R: (5′-GGACTACHVGGGTWTCTAAT-3′) fused with the appropriate Illumina adapters and a 12-bp index sequence unique to each sample^47^. All the PCR reactions were carried out in triplicate with about 10 ng metagenomic DNA per reaction. DNA amplicons were then combined in equimolar ratios into a single tube. A single library was generated for producing paired 250-nucleotide reads in an Illumina’s MiSeq platform (Illumina).

### Data processing and 16S rRNA gene-based community analysis

Data were processed according to the SOP pipelines of the software package Mothur with minor modifications^5^,^6^,^48^,^49^. The resulting high-quality sequences were aligned to SILVA reference alignment^50^ (Database release 119 updated Aug 8, 2014). Chimeric sequences were identified and removed with a *de novo* method. Sequences classified as Archaea, Eukaryota, chloroplasts, or mitochondria were also removed. A summary of data processing steps is provided in Supplementary Table S1.

For all the downstream analysis, we rarefied to 5632 randomly selected sequences per sample to correct the differences in sequencing depth. The selected sequences were clustered into 18921 OTUs (operational taxonomic units) using a 3% distance cutoff. Representative sequences in each OTU were aligned to the SILVA database. Taxonomy was subsequently assigned to each representative sequence using the SILVA database classifier with the minimum support threshold of 85%. Relative abundance of the first 100 most abundant OTUs in each sample was visualized by drawing heatmaps (Supplementary Fig. S2; Supplementary Table S2).

Rarefaction was performed using Mothur to discern levels of alpha diversity (diversity index and species richness estimator) for each sample (Supplementary Table S3). Rarefaction curves were separately calculated at 0.03, 0.05 and 0.1 distance levels (Supplementary Fig. S3), and statistical analysis was performed using a *t* test with *p* values to determine whether the diversity of observed OTUs was statistically significantly different among the four treatments: HRC, HRS, IRC and IRS (Supplementary Table S4). To estimate Beta diversity, Bray-Curtis dissimilarity matrix, Thetayc calculators and Pearson correlation coefficient (r values) were used to examine the similarity of the membership and structure found in the various samples. We used NMDS and PCoA plots to visualize differences in bacterial community composition among samples. A non-parametric T-test tool metastats in Mothur was used to determine the OTUs or taxa, which were differentially represented between the samples or were responsible for differences between healthy and infected samples.

### Sequencing, assembly and binning of the root gall-associated metagenome

A mate-pair shotgun library with a large insertion of 5 kb was constructed using the genomic DNA extracted from the enriched root gall-associated microbiome. Pair-end sequencing (2 × 100 bp) was performed by using Illumina Hiseq 2000 at Sangon Biotechnology Company (Shanghai, China). After trimming, one third of randomly extracted high quality reads were used to generate a preliminary assembly by using the CLC Genomic Workbench (v7.0.3, CLC bio) with default parameters. The assembly was searched against the NCBI nr database using blastx command with E-value cut-off of 1e-5 and then assigned the taxonomy using the NCBI taxonomy database. Of the assembled contigs, 127,083 (59%) were annotated and assigned the taxonomy (Supplementary Fig. S4A). Subsequently, the contigs belonging to different organisms (bacteria, nematodes, plants and fungi) were separated according to their GC content, coverage and taxonomic annotation information^51^,^52^,^53^. Extracting all the reads that mapped to the contigs belonging to bacteria generated a set of enriched bacteria-only sequencing data.

The extracted bacteria-only sequencing data were reassembled through Velvet 1.1.06 with optimized kerm 57 and average insert length of 4618 bp^54^. The assembly of the root gall-associated microbiome (above 200 bp) was assigned taxonomy, and then plotted with GC content, coverage and the assigned taxonomic information at the different taxonomic levels using a set of perl and R scripts^51^,^53^ (Supplementary Fig. S5). Based on the information of taxonomic assignments, coverage and GC content, the assembly was further grouped into different bacterial taxa (mainly at the Order level), and the remainder was combined as “Other Bacteria” (Supplementary Table S5). As above, the reads that mapped to the contigs belonging to different bacterial groups were extracted again. The filtered reads in pairs were used for scaffolding the contigs in each bacterial group^55^. The scaffolds from the different bacterial groups were collected and combined into the final metagenomic assembly to use for the following annotation and metagenomic analysis.

The extracted reads belonging to different bacterial groups were also used to determine the community profile of the endophyte microbiome associated with nematode-infected tomato root gall. To obtain a clearer insight into community composition of the endophyte microbiome in tomato root galls, hypervariable regions V3-V4 of the bacterial 16S rRNA gene were also amplified for community analysis using the genomic DNA from the root gall-associated microbiome according to the protocol described above.

### Gene prediction and annotation

The final metagenomic assembly for each bacterial group was uploaded into MG-RAST (Rapid Annotation using Subsystem Technology for Metagenomes) separately or as a whole for gene prediction and annotation^56^. To assess the completeness of the genomic content for each bacterial group, the minimal gene sets were determined according to the methods described by Engel *et al.*^57^. To evaluate the concentration of the gene contents specific to the predominant specie in each group, the annotated coding sequences were assigned to the reference genome of the closet relative using the function “Recruitment” in MG-RAST. The total statistics of metagenome, annotation and functional-based taxonomic hits distribution was summarized (Supplementary Fig. S6, Supplementary Table S5).

Plant cell wall-degrading enzymes, including cellulase, hemicellulase, pectinases, lignin-degrading enzymes etc, were annotated based on CAZymes (Carbohydrate-active enzymes) and KEGG databases using methods previously described^58^,^59^. The annotations for all predicted CAZymes were inspected manually, counted, and named according to the CAZY nomenclature.

### Functional characterization and metagenomic analysis

Assignment of functional categories and construction of KEGG pathway were performed in MG-RAST. To enrich the functional categories that were specific to a bacterial group, the relative abundance of functional categories based on the subsystem for the dominant bacterial groups with more than 60% genomic completeness in the tomato root gall-associated microbiome, including Sphingobacteriales, Flavobacteriales, Rhodocyclales, Burkholderiales, Rhizobiales, Enterobacteriales, Pseudomonadales and Sphingomonadales, were calculated on the basis of normalized gene counts by using the tool “Functional abundance” in MG-RAST server (Fig. 4A; Supplementary Table S11). The functional heatmap was also clustered using a maximum e-value of 1e-5, a minimum identity of 60%, and a minimum alignment length of 15 measured in amino acids for protein based on the Bray-Curtis distance metric (Fig. 4A).

### Nucleotide sequence accession numbers

The sequence data were deposited in MG-RAST server (Rapid Annotation using Subsystem Technology for Metagenomes) as name of endophytes_metagenome with MG-RAST ID: 4603086.

## Additional Information

**How to cite this article**: Tian, B.-Y. *et al.* Metagenomic insights into communities, functions of endophytes, and their associates with infection by root-knot nematode, *Meloidogyne incognita*, in tomato roots. *Sci. Rep.* **5**, 17087; doi: 10.1038/srep17087 (2015).

## Supplementary Material

Supplementary Information

## Figures and Tables

**Table 1:** KEEG database-based identification of the putative plant cell wall-degrading enzymes in the major taxa-based assemblies of the tomato root gall-associated microbiome.

EC	CAZymes	Metagenome	Spingoba	Entero	Flavo	Rhizo	Burk	Spingomo	Pseu	Rhodo	Others
3.2.1.1	α-amylase	16	5	4	2	2					1
3.2.1.10	oligo-1,6-glucosidase	2									3
3.2.1.122	maltose-6′-phosphate glucosidase	6		5		1					
3.2.1.147	thioglucosidase	3		3							
3.2.1.20	α-glucosidase	53	13	8	8	8	2	1			8
3.2.1.21	β-glucosidase	99	27	10	22	2	2	9	4	1	13
3.2.1.22	α-galactosidase	26	6	4	2	11					2
3.2.1.23	β-galactosidase	47	14	11	3	2					17
3.2.1.24	α-mannosidase	4		2							
3.2.1.25	β-mannosidas	18		2	7	6					2
3.2.1.26	β-fructofuranosidase	11		7		3			1		
3.2.1.28	α-trehalase	9		5							2
3.2.1.31	β-glucuronidase	1					1				
3.2.1.37	xylan 1,4-β-xylosidase	18	1	6	2	3			1		3
3.2.1.39	endo-1,3-β-D-glucosidase	2			2						
3.2.1.4	cellulase	15	2	5		3	1				4
3.2.1.45	glucosylceramidase	19	6		8		1				6
3.2.1.50	α-*N-*acetylglucosaminidase	2	2								
3.2.1.51	α-L-fucosidase	39	20		6						10
3.2.1.52	β-L-*N-*acetylhexosaminidase	71	17	9	3	10	6	1	2	1	15
3.2.1.55	α-L-arabinofuranosidase	10		2	2	2					7
3.2.1.58	glucan 1,3-β-glucosidase	3				1			2		
3.2.1.8	endo-β-1,4-xylanase	0									
3.2.1.86	6-phospho-β-glucosidase	18		18							
3.2.1.93	α-phosphotrehalase	5		3			1		1		
3.1.1.11	pectinesterase	8		1			1				3
3.2.1.67	Polygalacturonase	0									
4.2.2.2	pectate lyase	4		1	1		2				
4.2.2.3	poly(β-D-mannuronate) lyase	5		1	3						
4.2.2.6	oligogalacturonide lyase	6		3							
4.2.2.9	exo-pectate lyase	1		1							
4.2.2.10	pectin lyase	0									
1.11.1.13	Lignin Peroxidase	0									
1.11.1.14	Manganese Peroxidase	0									
1.10.3.2	Laccase	0									
Total		521	113	111	71	54	17	11	11	2	96

Notes: Sphingoba: Sphingobacteriales; Entero: Enterobacteriales; Flavo: Flavobacteriales; Rhizo: Rhizobiales; Burk: Burkholdales; Sphingomo: Sphingomonadales; Pseu: Pseudomonadales; Rhodo: Rhodocyclales; Others: Other bacteria.
