# Supplementary material for: Metagenomic insights into communities, functions of endophytes, and their associates with infection by root-knot nematode, Meloidogyne incognita, in tomato roots
Source: Sci Rep. 2015 Nov 25;5:17087. doi: 10.1038/srep17087 (PMC4658523; doi:10.1038/srep17087)
Supplement: Supplementary Information [file srep17087-s1.pdf]

Metagenomic insights into communities, functions of endophytes, and their associates with infection by root-knot nematode, *Meloidogyne incognita*, in tomato roots

Baoyu Tian[1,*], Yi Cao[2,3], Keqin Zhang[2,*]

Affiliations:

1 College of Life Science, Fujian Normal University, Fuzhou, 350108, Fujian, China

2 Key Laboratory for Conservation and Utilization of Bio-resources, and Key Laboratory for Microbial Resources of the Ministry of Education, Yunnan University, Kunming, 650091, Yunnan, China

3 Guizhou Academy of Tobacco Science, Guiyang, 550081, Guizhou, China

*** Corresponding author**:

Keqin Zhang

Address: Key Laboratory for Conservation and Utilization of Bio-resources, and Key Laboratory for Microbial Resources of the Ministry of Education, Yunnan University, No.2, North Green Lake Road, Wuhua District, Kunming, 650091, Yunnan, China

Tel: 86-0871-65034878

E-mail: kqzhang1@ynu.edu.cn

or

Baoyu Tian

Address: College of Life Science, Fujian Normal University, No.8, Shangsan Road, Cangshan District, Fuzhou, 350108, Fujian, China

Tel: 86-0591-22868196

E-mail: tianby@fjnu.edu.cn

Supplementary Figures

Figure S1. Diagram of the protocols to separately extract genomic DNA from the tomato root with root-knot nematode infection for 16S rRNA gene-based community analysis and sequencing-based metagenomic analysis.

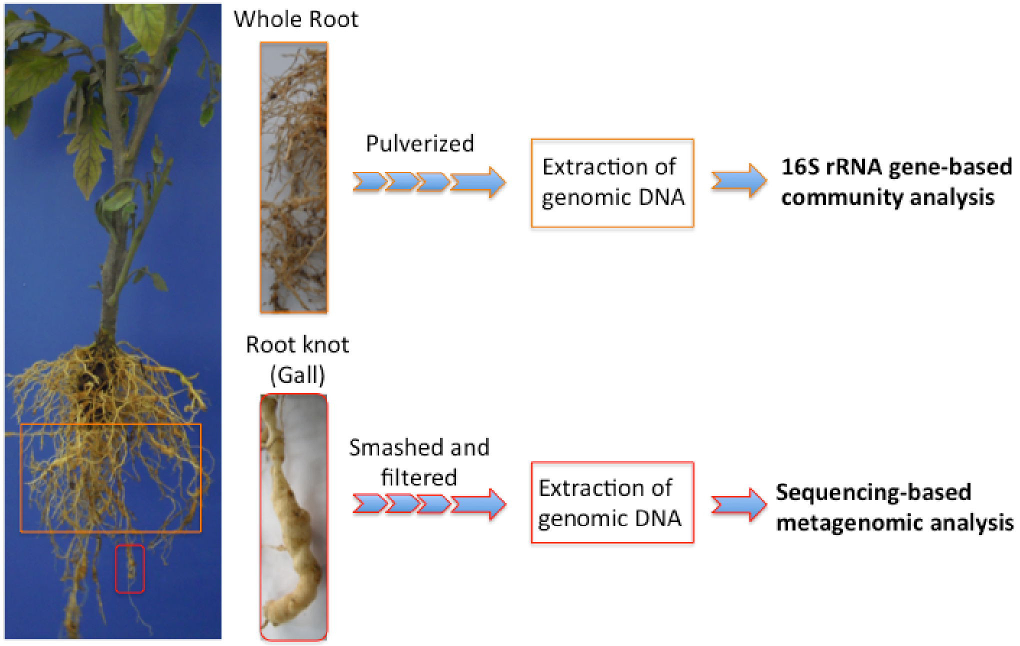

Figure S2. Heatmap depicting the most abundant 100 OUTs of the root-associated microbiome in healthy and nematode-infected tomato. Dendrogram linkages and distances of OTUs are not phylogenetic, but based upon reads number (log transformed) of OTUs within the samples. Legend and scale shown in the upper right corner of the figure represent colors in heat map associated with the relative percentage of bacteria (cluster of variables in Y-axis) within each nematode sample (X-axis clustering). HRC1-3: healthy tomato root endophytes; HRS1-3: healthy tomato rhizobacteria; IRC1-3: nematode-infected tomato root endophytes; IRS1-3: nematode-infected tomato rhizobacteria.

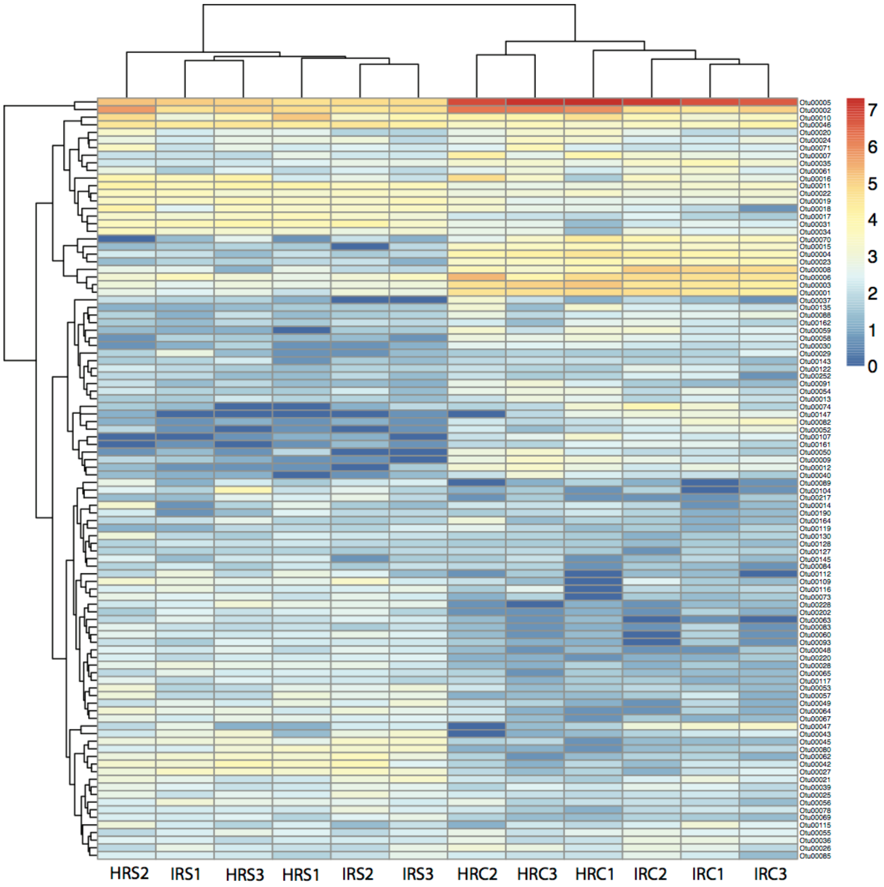

Figure S3. Rarefaction curves of the root-associated microbiome in healthy and nematode-infected tomato at 3, 5, and 10% distance cutoff, respectively. HRC1-3: healthy tomato root endophytes; HRS1-3: healthy tomato rhizobacteria; IRC1-3: nematode-infected tomato root endophytes; IRS1-3: nematode-infected tomato rhizobacteria.

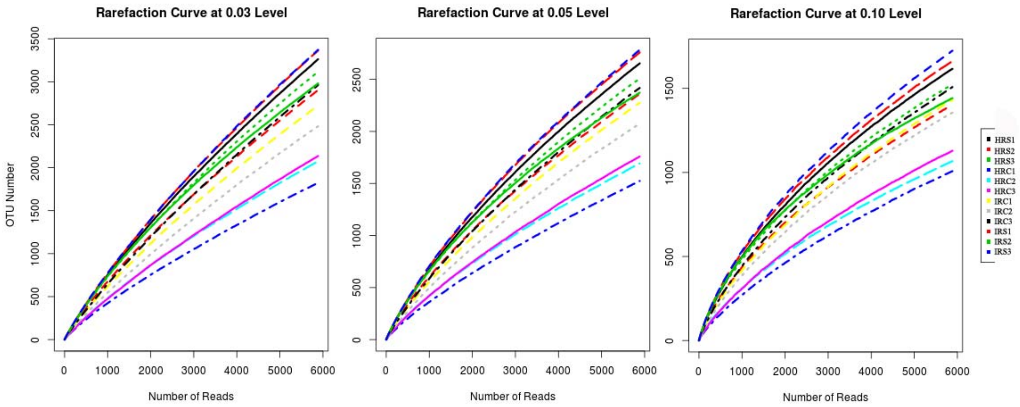

Figure S4. Taxonomic assignment of the assembled contigs and reads from the shotgun sequencing of the root gall-associated metagenome. (A) **Taxonomic assignment of the assembled contigs of the root gall-associated metagenome.** A preliminary assembly with 215,301 (>200bp) contigs was generated from Illumina sequencing data of the tomato root gall-associated metagenome. The assembled contigs were searched against the NCBI nr database using blastx command with E-value cut-off of 1e-5 and then assigned taxonomy using the NCBI taxonomy database. (B) **Taxonomic assignment of Illumina sequencing reads of root gall-associated metagenome.** The sequenced reads were remapped to the annotated contigs and then extracted according to their taxonomic assignments.

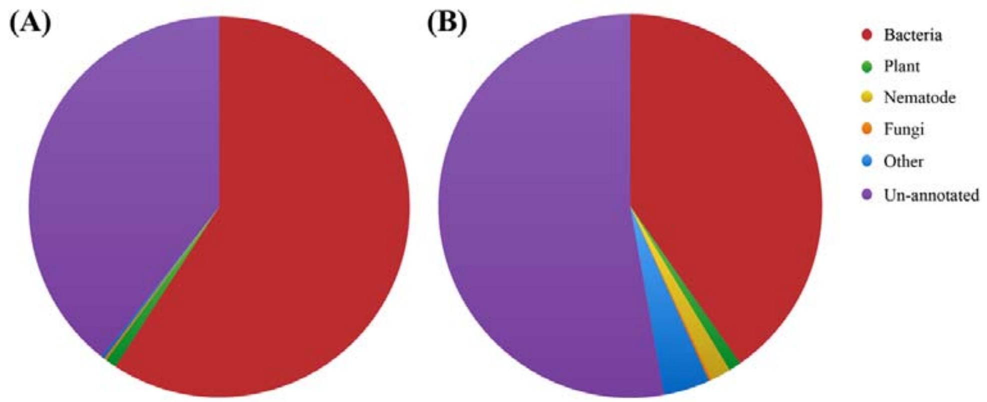

Figure S5. TAGC plots of the tomato root gall-associated microbiome assembly. The TAGG plots of bacterial assembly were produced based on their GC content, read coverage and taxonomic assignment at Phylum and Order levels. The taxonomic units above 2% of annotated contigs were marked with colors, and contigs without an annotation were grey. Finally, the dominant genera of the major bacterial groups (Order) in the tomato root gall-associated microbiome were represented.

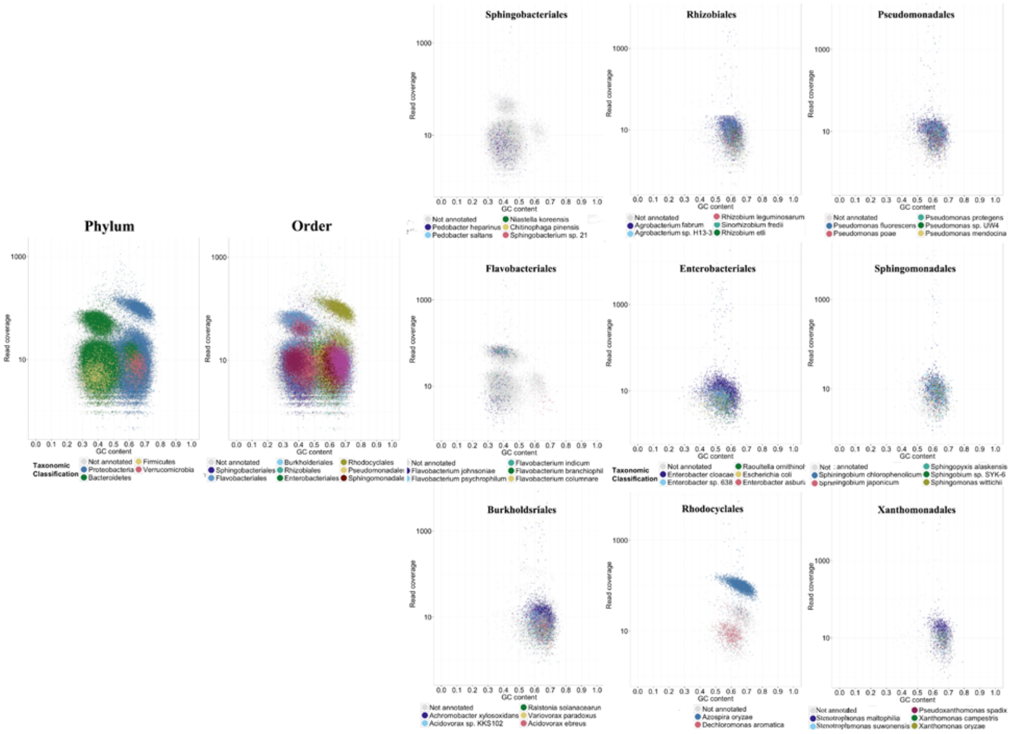

Figure S6. Taxonomic hits distribution for the functional annotation features of the tomato root gall-associated microbiome. Pie chart in the center illustrates the percentage of sequences with predicted functional features annotated to the indicated taxonomic level based on the databases in MG-RAST. The circular maps surrounding the central pie chart were generated by mapping the annotated features of the microbiome to proteins in a reference genome of the predominant species for each of the major bacterial groups in the root gall-associated microbiome to illustrate genome completeness and species concentration. Mapped/Total indicates the percentage of the sequences for each assembly of the metagenome to proteins in the corresponding reference genome.

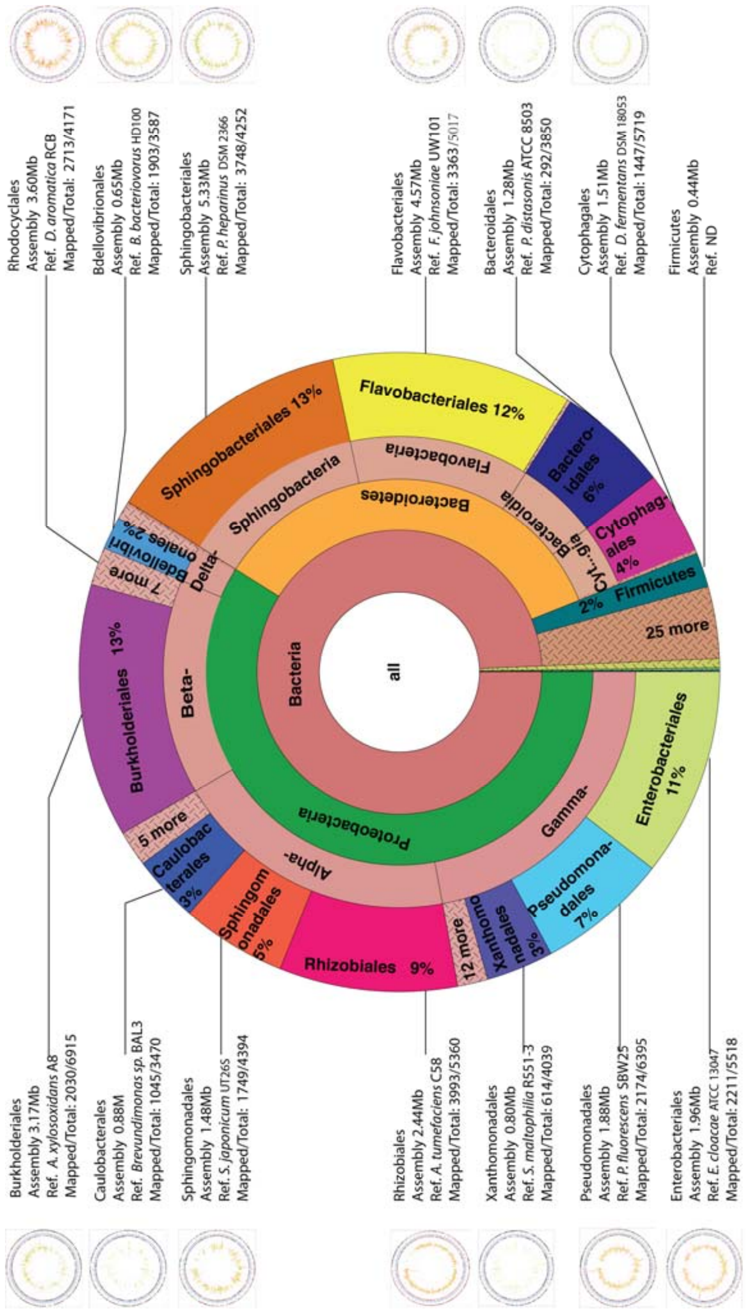

Figure S7. Functional category hits distribution of the tomato root gall-associated microbiome assembly. The pie charts illustrate the distribution of major functional categories of the metagenome. Each slice indicates the percentage of sequences assigned to the functional category in the subsystem database.

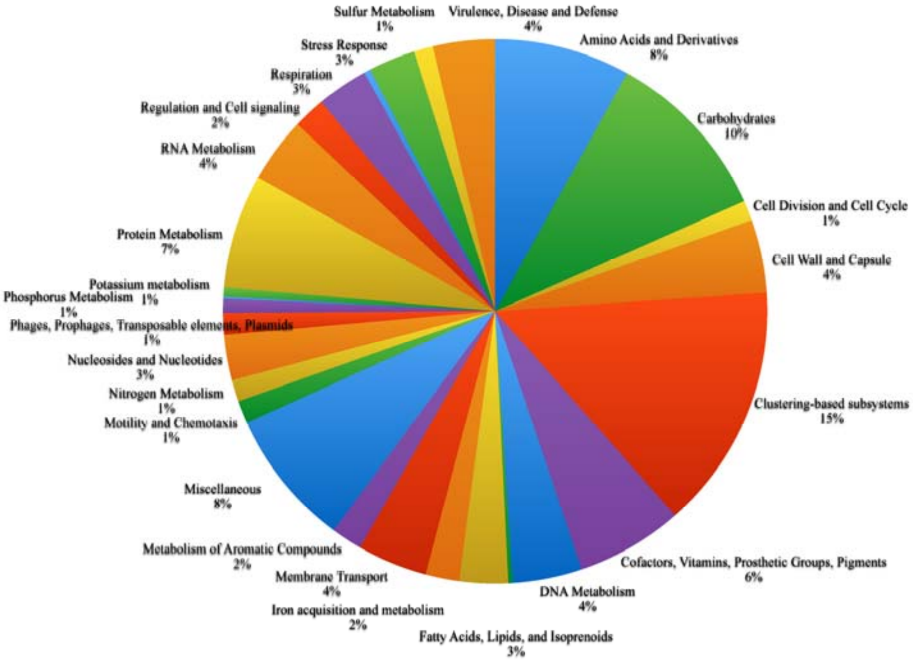

Supplementary Tables

Supplementary Table S1 Summary of data statistics and read processing steps.xls

Supplementary Table S2 Taxonomic annotation for the most abundant 100 OTUs in tomato rhizobactera and root endophytes.xls

Supplementary Table S3 Summary of alpha diversity for each sample.xls

Supplementary Table S4 statistical test results based on Shannon index.xls

Supplementary Table S5 Summary of assembly and minimal gene sets for the major bacterial groups in the root gall-associated metagenome.xls

Supplementary Table S6 The OTU list that represented the difference between the four treatments by metastatistical analysis.xls

Supplementary Table S7 List of annotation for the cell wall-degrading enzymes produced by the root gall-associated metagenome in CAZymes database.xls

Supplementary Table S8 Putative genes related to nitrogen metabolism in the tomato root gall-associated microbiome.xls

Supplementary Table S9 Putative genes related to pathways of synthesis of IAA (indole-3-acetic acid) in the tomato root gall-associated microbiome.xls

Supplementary Table S10 Putative genes related to biosynthesis of the siderophore group of non-ribosomal peptides in tomato root gall-associated microbiome.xls

Supplementary Table S11 Subsystem functional categories significantly enriched in major bacterial groups of the root gall-associated metagenome.xls

Supplementary Table S1 Summary of data statistics and read processing steps

Sample ID	Conditions	Treatment	Total reads	Combined reads	Percent Comb	Combined ba	Min.len	Max.len	Avg.len.	quality-filtere	chloroplast r	Percent chloroplast
HRC1	tomato root	Healthy	78180	77255	98.82	31700574	249	448	410	52875	20730	60.79
HRC2	tomato root	Healthy	54983	54218	98.61	22344272	247	450	412	30594	19061	37.70
HRC3	tomato root	Healthy	29658	29359	98.99	12047724	236	446	410	20516	7170	65.05
HRS1	rhizosphere s	Healthy	35728	35300	98.8	14589730	239	451	413	15772	15254	3.28
HRS2	rhizosphere s	Healthy	53171	52577	98.88	21718601	235	451	413	22883	22001	3.85
HRS3	rhizosphere s	Healthy	55854	55103	98.66	22782028	232	451	413	23239	22526	3.07
IRC1	tomato root	Nematode-infe	57892	57324	99.02	23426374	231	449	409	40887	5632	86.23
IRC2	tomato root	Nematode-infe	65377	64715	98.99	26465601	241	450	409	48223	8021	83.37
IRC3	tomato root	Nematode-infe	84639	83797	99.01	34237404	237	451	409	58538	7022	88.00
IRS1	rhizosphere s	Nematode-infe	36455	35929	98.56	14855201	246	451	413	15838	15020	5.16
IRS2	rhizosphere s	Nematode-infe	63893	63049	98.68	26059596	231	453	413	28863	27282	5.48
IRS3	rhizosphere s	Nematode-infe	40386	39773	98.48	16468815	231	451	414	17534	16558	5.57

Supplementary Table S2 Taxonomic annotation for the first 100 abundance OTUs in tomato rhizobactera and root endophytes

OTUNumber	HRC	HRS	IRC	IRS	out_all	OTUConTaxonomy						
Otu00005	3816	448	2739	424	7427	k__Bacteria(	p__Actinoba	c__Actinobacteria(1(	o__Actinomycetales(100)	f__Streptom	g__Streptom	unclassified(100)
Otu00002	1384	583	345	298	2610	k__Bacteria(	p__Proteoba	c__Gammaproteoba	o__Pseudomonadales(100)	f__Pseudom	g__Pseudom	s__umsongensis(100)
Otu00006	387	55	326	93	861	k__Bacteria(	p__Actinoba	c__Actinobacteria(1(	o__Actinomycetales(100)	f__Micromor	unclassified(	unclassified(100)
Otu00003	431	51	254	47	783	k__Bacteria(	p__Actinoba	c__Actinobacteria(1(	o__Actinomycetales(100)	f__Streptom	g__Streptom	s__reticuliscabiei(95)
Otu00010	230	342	95	93	760	k__Bacteria(	p__TM7(100	c__TM7-3(100)	o__(100)	f__(100)	g__(100)	s__(100)
Otu00046	98	263	126	243	730	k__Bacteria(	p__Proteoba	c__Alphaproteobact	o__Sphingomonadales(100)	f__Sphingom	g__Sphingob	s__(100)
Otu00001	340	35	276	45	696	k__Bacteria(	p__Actinoba	c__Actinobacteria(1(	o__Actinomycetales(100)	f__Streptom	g__Streptom	unclassified(64)
Otu00008	108	31	425	24	588	k__Bacteria(	p__Actinoba	c__Actinobacteria(1(	o__Actinomycetales(100)	f__Micromor	g__Actinopla	s__(100)
Otu00011	47	174	77	117	415	k__Bacteria(	p__Proteoba	c__Alphaproteobact	o__Sphingomonadales(100)	f__Sphingom	g__Sphingob	s__(100)
Otu00016	163	125	65	60	413	k__Bacteria(	p__Bacteroic	c__Flavobacteriia(10	o__Flavobacteriales(100)	f__Flavobact	g__Flavobact	s__succinicans(100)
Otu00004	213	19	160	17	409	k__Bacteria(	p__Actinoba	c__Actinobacteria(1(	o__Actinomycetales(100)	f__Streptom	g__Streptom	s__reticuliscabiei(75)
Otu00031	30	128	30	127	315	k__Bacteria(	p__Proteoba	c__Alphaproteobact	o__Sphingomonadales(100)	f__Sphingom	g__Kaistobac	s__(100)
Otu00022	64	83	58	95	300	k__Bacteria(	p__Proteoba	c__Alphaproteobact	o__Rhizobiales(100)	f__Rhizobiac	g__Rhizobiur	s__(80)
Otu00019	49	105	46	99	299	k__Bacteria(	p__Proteoba	c__Betaproteobacte	o__Burkholderiales(100)	f__Comamor	g__Rubriviva	s__(100)
Otu00018	52	127	17	74	270	k__Bacteria(	p__TM7(100	c__TM7-1(100)	o__(100)	f__(100)	g__(100)	s__(100)
Otu00070	115	13	119	12	259	k__Bacteria(	p__Actinoba	c__Actinobacteria(1(	o__Actinomycetales(100)	f__Micromor	g__Couchiop	s__(97)
Otu00023	110	14	115	14	253	k__Bacteria(	p__Actinoba	c__Actinobacteria(1(	o__Actinomycetales(100)	f__Streptom	g__Streptom	s__mirabilis(85)
Otu00007	134	28	51	23	236	k__Bacteria(	p__Actinoba	c__Actinobacteria(1(	o__Actinomycetales(100)	f__Micromor	g__Couchiop	s__(69)
Otu00035	62	27	93	37	219	k__Bacteria(	p__Proteoba	c__Alphaproteobact	o__Rhizobiales(100)	f__Rhizobiac	unclassified(	unclassified(99)
Otu00034	32	82	41	60	215	k__Bacteria(	p__Proteoba	c__Alphaproteobact	o__Rhizobiales(100)	f__Hyphomic	g__Devosia(	s__(100)
Otu00015	103	12	84	10	209	k__Bacteria(	p__Actinoba	c__Actinobacteria(1(	o__Actinomycetales(100)	f__Streptom	g__Streptom	s__reticuliscabiei(82)
Otu00017	21	97	20	70	208	k__Bacteria(	p__Proteoba	c__Alphaproteobact	o__Sphingomonadales(100)	f__Sphingom	g__Kaistobac	s__(92)
Otu00042	13	99	19	74	205	k__Bacteria(	p__Proteoba	c__Alphaproteobact	o__Sphingomonadales(100)	f__Sphingom	g__Kaistobac	s__(100)
Otu00027	14	71	20	84	189	k__Bacteria(	p__Proteoba	c__Alphaproteobact	o__Sphingomonadales(100)	f__Sphingom	g__Kaistobac	s__(98)
Otu00024	70	47	36	33	186	k__Bacteria(	p__Actinoba	c__Actinobacteria(1(	o__Actinomycetales(100)	f__Micrococ	g__(67)	s__(67)
Otu00071	63	58	26	26	173	k__Bacteria(	p__Proteoba	c__Alphaproteobact	o__Rhizobiales(100)	f__Rhizobiac	g__Agrobact	s__(85)
Otu00020	68	52	26	19	165	k__Bacteria(	p__Proteoba	c__Alphaproteobact	o__Rhizobiales(100)	f__Rhizobiac	g__Agrobact	s__(77)
Otu00062	9	67	12	76	164	k__Bacteria(	p__Chlorofle	c__TK17(100)	o__mle1-48(100)	f__(100)	g__(100)	s__(100)
Otu00021	18	33	37	59	147	k__Bacteria(	p__Proteoba	c__Alphaproteobact	o__Rhizobiales(100)	f__Bradyrhiz	g__Bradyrhiz	s__(59)
Otu00045	12	51	9	75	147	k__Bacteria(	p__Gemmati	c__Gemmatimonade	o__Gemmatimonadales(92)	unclassified(	unclassified(	unclassified(87)
Otu00061	40	33	49	20	142	k__Bacteria(	p__Proteoba	c__Alphaproteobact	o__Rhizobiales(100)	f__Rhizobiac	g__Shinella(	s__granuli(90)
Otu00080	5	60	13	61	139	k__Bacteria(	p__Acidobac	c__Chloracidobacter	o__RB41(100)	f__Ellin6075(	g__(100)	s__(100)
Otu00026	44	22	35	34	135	k__Bacteria(	p__Actinoba	c__Actinobacteria(1(	o__Actinomycetales(100)	f__Streptom	g__Streptom	s__reticuliscabiei(95)
Otu00109	10	49	18	55	132	k__Bacteria(	p__Proteoba	c__Alphaproteobact	o__Rhizobiales(100)	f__Bradyrhiz	g__Bosea(10	s__genosp.(99)
Otu00055	34	31	36	26	127	k__Bacteria(	p__Proteoba	c__Betaproteobacte	o__Burkholderiales(100)	f__Comamor	g__Acidovor	unclassified(77)
Otu00036	28	26	44	27	125	k__Bacteria(	p__Proteoba	c__Alphaproteobact	o__Rhizobiales(100)	f__Hyphomic	g__Devosia(	s__(100)
Otu00039	24	30	30	38	122	k__Bacteria(	p__Proteoba	c__Alphaproteobact	o__Rhizobiales(100)	f__Rhizobiac	unclassified(	unclassified(91)
Otu00047	8	10	71	33	122	k__Bacteria(	p__Proteoba	c__Betaproteobacte	o__Burkholderiales(100)	f__Comamor	g__Acidovor	s__(96)
Otu00056	26	37	16	41	120	k__Bacteria(	p__Proteoba	c__Gammaproteoba	o__Xanthomonadales(100)	f__Xanthom	g__Dokdone	s__(100)
Otu00025	22	37	21	39	119	k__Bacteria(	p__Proteoba	c__Alphaproteobact	o__Rhizobiales(100)	f__Hyphomic	g__Devosia(	s__(100)
Otu00074	33	4	71	11	119	k__Bacteria(	p__Actinoba	c__Actinobacteria(1(	o__Actinomycetales(100)	f__Micromor	g__Actinopla	s__(90)
Otu00043	5	32	23	57	117	k__Bacteria(	p__Gemmati	c__Gemm-1(100)	o__(100)	f__(100)	g__(100)	s__(100)
Otu00009	66	9	34	5	114	k__Bacteria(	p__Actinoba	c__Actinobacteria(1(	o__Actinomycetales(100)	f__Streptom	g__Streptom	s__reticuliscabiei(52)
Otu00012	65	5	37	5	112	k__Bacteria(	p__Actinoba	c__Actinobacteria(1(	o__Actinomycetales(100)	f__Streptom	g__Streptom	unclassified(67)
Otu00064	11	46	8	46	111	k__Bacteria(	p__Proteoba	c__Alphaproteobact	o__Sphingomonadales(100)	f__Sphingom	unclassified(	unclassified(94)
Otu00115	16	34	41	19	110	k__Bacteria(	p__Proteoba	c__Betaproteobacte	o__Burkholderiales(100)	f__Comamor	g__Acidovor	s__delafieldii(81)
Otu00059	51	5	42	10	108	k__Bacteria(	p__Actinoba	c__Actinobacteria(1(	o__Actinomycetales(100)	f__Streptom	g__Streptom	unclassified(91)
Otu00054	48	16	26	17	107	k__Bacteria(	p__Actinoba	c__Actinobacteria(1(	o__Actinomycetales(100)	f__Micrococ	g__(78)	s__(78)
Otu00053	15	39	13	36	103	k__Bacteria(	p__TM7(100	c__TM7-1(100)	o__(100)	f__(100)	g__(100)	s__(100)
Otu00085	23	23	25	30	101	k__Bacteria(	p__Proteoba	c__Betaproteobacte	o__Burkholderiales(100)	f__Comamor	unclassified(	unclassified(100)
Otu00057	8	44	15	33	100	k__Bacteria(	p__TM7(100	c__TM7-1(100)	o__(100)	f__(100)	g__(100)	s__(100)
Otu00116	16	27	16	41	100	k__Bacteria(	p__Proteoba	c__Alphaproteobact	o__Rhizobiales(100)	f__Hyphomic	g__Rhodopla	s__(100)
Otu00104	7	64	6	22	99	k__Bacteria(	p__TM7(100	c__TM7-3(100)	o__I025(100)	f__(100)	g__(100)	s__(100)
Otu00028	10	41	8	39	98	k__Bacteria(	p__Proteoba	c__Alphaproteobact	o__Sphingomonadales(100)	f__Sphingom	g__Kaistobac	s__(92)
Otu00049	14	32	9	43	98	k__Bacteria(	p__Proteoba	c__Gammaproteoba	o__Xanthomonadales(100)	f__Xanthom	g__(100)	s__(100)
Otu00078	14	26	22	36	98	k__Bacteria(	p__Proteoba	c__Alphaproteobact	o__Rhizobiales(100)	f__Bradyrhiz	g__Bradyrhiz	s__(80)
Otu00058	49	8	31	9	97	k__Bacteria(	p__Actinoba	c__Actinobacteria(1(	o__Actinomycetales(100)	f__Actinosyn	unclassified(	unclassified(75)
Otu00082	21	5	67	4	97	k__Bacteria(	p__Actinoba	c__Actinobacteria(1(	o__Actinomycetales(100)	f__Micromor	g__Actinopla	s__(96)
Otu00088	31	15	40	11	97	k__Bacteria(	p__Proteoba	c__Gammaproteoba	o__Pseudomonadales(100)	f__Pseudom	g__Pseudom	unclassified(90)
Otu00135	50	9	26	10	95	k__Bacteria(	p__Actinoba	c__Actinobacteria(1(	o__Actinomycetales(100)	f__Streptom	g__Streptom	unclassified(71)
Otu00067	12	42	9	30	93	k__Bacteria(	p__Proteoba	c__Alphaproteobact	o__Sphingomonadales(100)	f__Sphingom	g__Sphingob	s__(99)
Otu00228	3	43	7	36	89	k__Bacteria(	p__Bacteroic	c__Saprospirae(100)	o__Saprospirales(100)	f__Chitinoph	g__Flavisolib	s__(100)
Otu00013	36	17	22	12	87	k__Bacteria(	p__Actinoba	c__Actinobacteria(1(	o__Actinomycetales(100)	f__Streptom	g__Streptom	unclassified
Otu00073	7	35	11	33	86	k__Bacteria(	p__Proteoba	c__Alphaproteobact	o__Sphingomonadales(100)	f__Erythroba	g__(84)	s__(84)
Otu00014	24	42	10	8	84	k__Bacteria(	p__TM7(100	c__TM7-3(100)	o__(100)	f__(100)	g__(100)	s__(100)
Otu00069	11	26	12	34	83	k__Bacteria(	p__Proteoba	c__Gammaproteoba	o__Xanthomonadales(100)	f__Xanthom	g__Thermon	s__(100)
Otu00162	24	14	27	16	81	k__Bacteria(	p__TM7(100	c__TM7-3(100)	o__(100)	f__(100)	g__(100)	s__(100)
Otu00050	50	8	17	3	78	k__Bacteria(	p__Actinoba	c__Actinobacteria(1(	o__Actinomycetales(100)	f__Streptom	unclassified(	unclassified(94)
Otu00060	7	30	6	35	78	k__Bacteria(	p__Proteoba	c__Alphaproteobact	o__Sphingomonadales(100)	f__Sphingom	g__Kaistobac	s__(100)
Otu00091	44	16	10	8	78	k__Bacteria(	p__Proteoba	c__Gammaproteoba	o__Pseudomonadales(100)	f__Pseudom	g__Pseudom	unclassified(97)
Otu00107	43	3	30	2	78	k__Bacteria(	p__Actinoba	c__Actinobacteria(1(	o__Actinomycetales(100)	f__Streptom	g__Streptom	s__reticuliscabiei(98)
Otu00130	14	32	11	21	78	k__Bacteria(	p__Proteoba	c__Betaproteobacte	o__Burkholderiales(100)	f__Oxalobac	g__(100)	s__(100)
Otu00122	16	16	27	16	75	k__Bacteria(	p__Proteoba	c__Alphaproteobact	o__Rhizobiales(100)	f__Rhizobiac	g__Agrobact	s__(86)
Otu00164	26	24	9	16	75	k__Bacteria(	p__Proteoba	c__Alphaproteobact	o__Rhizobiales(100)	f__Hyphomic	g__Devosia(	s__(100)
Otu00065	10	25	9	29	73	k__Bacteria(	p__Proteoba	c__Alphaproteobact	o__Sphingomonadales(100)	f__Sphingom	g__Kaistobac	s__(99)
Otu00147	20	2	49	1	72	k__Bacteria(	p__Actinoba	c__Actinobacteria(1(	o__Actinomycetales(100)	f__Streptom	g__Streptom	unclassified(85)
Otu00048	7	29	6	29	71	k__Bacteria(	p__Proteoba	c__Alphaproteobact	o__Sphingomonadales(100)	f__Sphingom	g__Kaistobac	s__(100)
Otu00117	12	31	9	19	71	k__Bacteria(	p__Proteoba	c__Alphaproteobact	o__Rhizobiales(100)	f__Phyllobac	g__Mesorhiz	s__(89)
Otu00112	5	28	6	31	70	k__Bacteria(	p__Proteoba	c__Alphaproteobact	o__Sphingomonadales(100)	f__Sphingom	g__Kaistobac	s__(100)
Otu00030	35	7	21	5	68	k__Bacteria(	p__Actinoba	c__Actinobacteria(1(	o__Actinomycetales(100)	f__Streptom	g__Streptom	unclassified
Otu00052	18	7	41	2	68	k__Bacteria(	p__Actinoba	c__Actinobacteria(1(	o__Actinomycetales(100)	f__Streptom	g__Streptom	unclassified(93)
Otu00220	5	28	8	27	68	k__Bacteria(	p__Proteoba	c__Alphaproteobact	o__Ellin329(100)	f__(100)	g__(100)	s__(100)
Otu00040	37	5	18	6	66	k__Bacteria(	p__Actinoba	c__Actinobacteria(1(	o__Actinomycetales(100)	f__Streptom	g__Streptom	unclassified(55)
Otu00190	18	23	10	13	64	k__Bacteria(	p__TM7(100	c__TM7-3(100)	unclassified(99)	unclassified(	unclassified(	unclassified(99)
Otu00252	16	12	21	15	64	k__Bacteria(	p__Proteoba	c__Gammaproteoba	o__Xanthomonadales(100)	f__Xanthom	g__Thermon	s__(99)
Otu00119	21	14	11	17	63	k__Bacteria(	p__Proteoba	c__Gammaproteoba	o__Xanthomonadales(100)	f__Xanthom	g__Rhodanol	s__(77)
Otu00029	13	9	20	20	62	k__Bacteria(	p__Proteoba	c__Alphaproteobact	o__Sphingomonadales(100)	f__Sphingom	g__Sphingob	s__(96)
Otu00037	32	14	10	5	61	k__Bacteria(	p__Bacteroic	c__Flavobacteriia(10	o__Flavobacteriales(100)	f__Flavobact	g__Flavobact	s__succinicans(97)
Otu00128	12	20	10	19	61	k__Bacteria(	p__Proteoba	c__Alphaproteobact	o__Rhizobiales(100)	f__Bradyrhiz	g__Bosea(97	s__genosp.(96)
Otu00145	12	29	12	8	61	k__Bacteria(	p__Proteoba	c__Alphaproteobact	o__Rhizobiales(100)	f__Rhizobiac	unclassified(	unclassified(91)
Otu00202	4	21	8	28	61	k__Bacteria(	p__Actinoba	c__Acidimicrobiia(10	o__Acidimicrobiales(100)	f__C111(100	g__(100)	s__(100)
Otu00217	6	25	6	24	61	k__Bacteria(	p__Cyanoba	c__ML635J-21(100)	o__(100)	f__(100)	g__(100)	s__(100)
Otu00063	7	20	1	32	60	k__Bacteria(	p__Acidobac	c__Solibacteres(100)	o__Solibacterales(100)	f__(100)	g__(100)	s__(100)
Otu00093	8	27	5	20	60	k__Bacteria(	p__Proteoba	c__Alphaproteobact	o__Sphingomonadales(100)	f__Sphingom	g__Kaistobac	s__(99)
Otu00143	20	14	14	12	60	k__Bacteria(	p__Proteoba	c__Alphaproteobact	o__Rhizobiales(100)	f__Hyphomic	g__Devosia(	s__(99)
Otu00089	7	28	3	20	58	k__Bacteria(	p__Proteoba	c__Alphaproteobact	o__Sphingomonadales(100)	f__Sphingom	g__Kaistobac	s__(99)
Otu00083	6	25	8	18	57	k__Bacteria(	p__Proteoba	c__Alphaproteobact	o__Sphingomonadales(100)	f__Sphingom	unclassified(	unclassified(71)
Otu00084	12	17	8	20	57	k__Bacteria(	p__Proteoba	c__Gammaproteoba	o__Xanthomonadales(100)	f__Xanthom	g__Rhodanol	s__(88)
Otu00127	16	15	11	15	57	k__Bacteria(	p__Proteoba	c__Alphaproteobact	o__Rhizobiales(100)	unclassified(	unclassified(	unclassified(100)
Otu00161	22	1	29	5	57	k__Bacteria(	p__Actinoba	c__Actinobacteria(1(	o__Actinomycetales(100)	f__Streptom	unclassified(	unclassified(97)

Supplementary Table S3 Summary of alpha diversity for each sample

Sample ID	label	nseqs	num_otus	coverage	ace	simpson	invsimpson	shannon	chao
hRc1	0.03	5632	1767	74.41%	21007.48	0.0787	12.71	5.11	9627.00
hRc2	0.03	5632	1956	72.16%	18747.65	0.0410	24.41	5.64	8724.15
hRc3	0.03	5632	2044	69.94%	22738.63	0.0678	14.75	5.41	10519.02
HRS1	0.03	5632	3195	54.47%	30917.89	0.0028	357.21	7.40	13495.21
HRS2	0.03	5632	2901	61.01%	23044.34	0.0058	172.87	7.02	11026.63
HRS3	0.03	5632	3049	57.65%	22854.26	0.0027	374.94	7.33	11586.30
IRC1	0.03	5632	2582	62.62%	28686.50	0.0271	36.91	6.42	12294.54
IRC2	0.03	5632	2406	64.60%	28964.99	0.0429	23.33	6.03	11479.16
IRC3	0.03	5632	2838	58.47%	30651.92	0.0192	52.00	6.69	13775.16
IRS1	0.03	5632	3239	54.42%	26861.42	0.0021	482.30	7.50	13280.04
IRS2	0.03	5632	2861	62.04%	18120.68	0.0019	514.37	7.34	9934.61
IRS3	0.03	5632	3152	56.53%	23796.52	0.0018	545.19	7.48	12092.68

Supplementary Table S4 statistical test results based on Shannon index

Summary of t-tests for difference in alpha diversity among different treatments

	Mean	SD	SEM	N
HRC	5.38	0.2658	0.1534	3
HRS	7.25	0.2022	0.1168	3
IRC	6.38	0.3318	0.1916	3
IRS	7.44	0.0872	0.0503	3

	t-ratio	df	standard error	P value
Diversity index				
shannon index				
HRC-HRS	9.6638	4	0.193	0.0006
IRC-IRS	5.3515	4	0.198	0.0059
HRC-IRC	4.047	4	0.245	0.0155
IRS-HRS	1.4943	4	0.127	0.2094

Supplementary Table S5 Summary of assembly and minimal gene sets for the major bacterial groups in root gall-associated metagenome

Groups	Taxonomy	Size (bp)	Number of Scaffo	GC content ('	Predicted Pro	the closest re	ID	Conserved m	mini gene se	Completenes	mapped	Completenes	align/total
Sphingobacteriales	Bacteroidetes	5332719	18206	41.02+5.79	17946	Pedobacter heparinus DSM		193	178	92%	3748	88%	21%
Flavobacteriales	Bacteroidetes	4568585	13098	40.21+7.03	13682	Flavobacterium johnsoniae		195	176	90%	3363	66%	25%
Rhodocyclales	Betaproteobacteria	3250561	6706	61.93+6.58	7180	Dechloromonas suillum PS		189	165	87%	3445	86%	48%
Burkholderiales	Betaproteobacteria	3173248	11252	63.45+5.38	11689	Achromobacter xylosoxida		193	158	82%	2030	29%	17%
Rhizobiales	Alphaproteobacteria	2435488	8908	60.14+4.59	8988	Agrobacterium tumefacien		186	141	76%	3993	74%	44%
Enterobacteriales	Gammaproteobacteria	1956759	6957	53.92+6.00	7258	Enterobacter cloacae subs		203	128	63%	2211	40%	30%
Pseudomonadales	Gammaproteobacteria	1879980	6450	60.16+5.34	6735	Pseudomonas fluorescens		198	120	61%	2174	34%	32%
Cytophagales	Bacteroidetes	1510755	5008	44.71+7.31	4915	Dyadobacter fermentans D		194	105	54%	1447	25%	29%
Sphingomonadales	Alphaproteobacteria	1480909	5320	61.67+4.08	5342	Sphingobium japonicum U		191	121	63%	1749	40%	33%
Bacteroidales	Bacteroidetes	1280132	4241	39.91+7.24	4153	Parabacteroides distasonis		192	119	62%	292	8%	7%
Caulobacterales	Alphaproteobacteria	875789	3205	66.54+4.17	3408	Brevundimonas sp. BAL3 (		nd	nd	nd	1045	30%	31%
Xanthomonadales	Gammaproteobacteria	807687	2688	64.91+4.69	2849	Stenotrophomonas maltop		nd	nd	nd	614	15%	22%
Bdellovibrionales	Deltaproteobacteria	648541	2198	43.99+3.42	2317	Bdellovibrio bacteriovorus		nd	nd	nd	1903	53%	82%
Firmicutes	Firmicutes	437820	1689	41.94+8.77	1509	nd		nd	nd	nd		nd	nd
Actinobacteria	Actinobacteria	188217	732	61.09+7.18	754	nd		nd	nd	nd		nd	nd
otherBacteria	nd	2589139	8535	57.18+10.51	7200	nd		nd	nd	nd		nd	nd

Supplementary Table S6 The OTU list that represented the difference between the four treatments by metastats analysis

OTUNumber	HRS	HRC	IRC	IRS		p-value (HRC	p-value (HRS	p-value (HRC	p-value (IRC-	OTUConTaxonomy					
Otu00001	494	4478	3048	489	16529			0.00433	0.005834	Bacteria(100	Actinobacter	Actinobacter	Streptomyce	Streptomyce	Streptomyces(98)
Otu00003	623	1343	356	291	4935	0.003177		0.025517		Bacteria(100	Proteobacter	Gammaprote	Pseudomona	Pseudomona	Pseudomonas(100)
Otu00002	68	639	412	55	2293			0.006055	0.007018	Bacteria(100	Actinobacter	Actinobacter	Streptomyce	Streptomyce	Streptomyces(89)
Otu00004	72	410	337	94	1732			0.044037	0.006348	Bacteria(100	Actinobacter	Actinobacter	Micromonos	Micromonos	unclassified(100)
Otu00007	305	126	162	273	1459			0.008157		Bacteria(100	Proteobacter	Alphaproteol	Sphingomon	Sphingomon	Sphingobium(100)
Otu00014	331	223	83	95	1369	0.043438				Bacteria(100	Candidate_d	unclassified(	unclassified(	unclassified(	unclassified(100)
Otu00005	28	118	471	34	1268	0.004781		0.012855	0.00412	Bacteria(100	Actinobacter	Actinobacter	Micromonos	Micromonos	Actinoplanes(91)
Otu00010	181	51	81	149	775	0.039577		0.005441	0.006907	Bacteria(100	Proteobacter	Alphaproteol	Sphingomon	Sphingomon	Sphingobium(100)
Otu00009	183	39	45	155	689			0.005586	0.005499	Bacteria(100	Proteobacter	Alphaproteol	Sphingomon	Sphingomon	Sphingomonas(99)
Otu00016	20	150	138	23	639			0.03524	0.004321	Bacteria(100	Actinobacter	Actinobacter	Micromonos	Micromonos	unclassified(100)
Otu00018	87	82	59	97	553	0.010799			0.030885	Bacteria(100	Proteobacter	Alphaproteol	Rhizobiales(1	Rhizobiaceae	Rhizobium(99)
Otu00021	16	113	129	17	533			0.007298	0.003138	Bacteria(100	Actinobacter	Actinobacter	Streptomyce	Streptomyce	Streptomyces(95)
Otu00069	102	38	57	80	474			0.007123		Bacteria(100	Proteobacter	Betaproteob	Burkholderia	Comamonad	Azohydromonas(88)
Otu00031	45	59	92	26	418				0.027583	Bacteria(100	Proteobacter	Alphaproteol	Rhizobiales(1	Rhizobiaceae	Shinella(83)
Otu00020	115	22	19	68	380		0.002881	0.003154	0.004181	Bacteria(100	Proteobacter	Alphaproteol	Sphingomon	Sphingomon	Sphingomonas(94)
Otu00022	9	92	79	14	374			0.013429	0.002605	Bacteria(100	Actinobacter	Actinobacter	Streptomyce	Streptomyce	Streptomyces(78)
Otu00039	89	41	15	69	359	0.032226			0.025989	Bacteria(100	Candidate_d	unclassified(	unclassified(	unclassified(	unclassified(100)
Otu00046	98	18	20	85	357			0.013284	0.032819	Bacteria(100	Proteobacter	Alphaproteol	Sphingomon	Sphingomon	Sphingomonas(99)
Otu00030	73	34	38	64	354				0.002821	Bacteria(100	Proteobacter	Alphaproteol	Rhizobiales(1	Hyphomicrob	Devosia(92)
Otu00011	20	100	41	18	340				0.033924	Bacteria(100	Actinobacter	Actinobacter	Micromonos	Micromonos	unclassified(99)
Otu00017	9	77	58	12	300			0.003634	0.007465	Bacteria(100	Actinobacter	Actinobacter	Streptomyce	Streptomyce	Streptomyces(97)
Otu00033	26	43	65	25	293				0.030641	Bacteria(100	Proteobacter	Alphaproteol	Rhizobiales(1	Rhizobiaceae	Shinella(97)
Otu00038	0	29	110	3	281			0.032361		Bacteria(100	unclassified(	unclassified(	unclassified(	unclassified(	unclassified(100)
Otu00006	4	71	60	8	278			0.015273	0.012223	Bacteria(100	Actinobacter	Actinobacter	Streptomyce	Streptomyce	Streptomyces(78)
Otu00034	46	62	20	18	274	0.031237				Bacteria(100	Proteobacter	Alphaproteol	Rhizobiales(1	Rhizobiaceae	Rhizobium(88)
Otu00008	8	58	60	12	264			0.015201	0.030474	Bacteria(100	Actinobacter	Actinobacter	Streptomyce	Streptomyce	Streptomyces(81)
Otu00028	61	12	22	73	263	0.038609		0.026579	0.006403	Bacteria(100	Proteobacter	Alphaproteol	Sphingomon	Sphingomon	Sphingomonas(99)
Otu00055	75	6	16	66	260	0.048612		0.033686	0.040274	Bacteria(100	Acidobacteri	Acidobacteri	Subgroup_4(	Unknown_Fa	Blastocatella(100)
Otu00053	9	22	87	8	244	0.028006			0.015009	Bacteria(100	Actinobacter	Actinobacter	Micromonos	Micromonos	Actinoplanes(79)
Otu00047	61	6	9	85	237			0.005659	0.002676	Bacteria(100	Chloroflexi(1	TK10(100)	unclassified(	unclassified(	unclassified(100)
Otu00073	60	13	16	52	230			0.012994	0.006756	Bacteria(100	Proteobacter	Alphaproteol	Rhizobiales(1	Bradyrhizobi	Rhodopseudomonas(100)
Otu00024	7	62	39	5	221			0.007196	0.005666	Bacteria(100	Actinobacter	Actinobacter	Streptomyce	Streptomyce	Streptomyces(99)
Otu00043	33	24	40	25	219	0.003672			0.015117	Bacteria(100	Proteobacter	Alphaproteol	Rhizobiales(1	Hyphomicrob	Devosia(97)
Otu00051	55	10	11	63	215			0.032216	0.003323	Bacteria(100	Gemmatimo	Gemmatimo	Gemmatimo	Gemmatimo	Gemmatimonas(100)
Otu00067	23	56	18	17	211	0.00981		0.035168		Bacteria(100	Actinobacter	Actinobacter	Streptomyce	Streptomyce	Streptomyces(82)
Otu00095	11	13	58	41	205	0.010443	0.004751			Bacteria(100	Proteobacter	Betaproteob	Burkholderia	Comamonad	Acidovorax(85)
Otu00036	49	12	16	47	201			0.005535	0.030529	Bacteria(100	Proteobacter	Alphaproteol	Rhizobiales(1	Phyllobacteri	Mesorhizobium(89)
Otu00066	43	12	21	40	192			0.015436		Bacteria(100	Proteobacter	Alphaproteol	Rhizobiales(1	Bradyrhizobi	unclassified(97)
Otu00059	11	47	28	10	182				0.026405	Bacteria(100	Actinobacter	Actinobacter	Pseudonocar	Pseudonocar	unclassified(77)
Otu00042	42	11	10	49	175			0.003226	0.003417	Bacteria(100	Proteobacter	Alphaproteol	Sphingomon	Sphingomon	Sphingomonas(90)
Otu00012	11	43	24	12	168	0.032065		0.006444		Bacteria(100	Actinobacter	Actinobacter	Streptomyce	Streptomyce	Streptomyces(86)
Otu00080	26	38	8	21	165				0.03087	Bacteria(100	Proteobacter	Alphaproteol	Rhizobiales(1	Hyphomicrob	Devosia(99)
Otu00045	29	3	21	58	164	0.032123		0.031999	0.039899	Bacteria(100	Gemmatimo	Gemmatimo	Gemmatimo	Gemmatimo	unclassified(100)
Otu00120	29	23	11	37	163				0.002788	Bacteria(100	Proteobacter	Alphaproteol	Rhizobiales(1	Xanthobacte	Pseudolabrys(100)
Otu00071	4	25	50	3	161				0.003026	Bacteria(100	Actinobacter	Actinobacter	Streptomyce	Streptomyce	Streptomyces(90)
Otu00078	18	46	10	7	155	0.005483	0.035829	0.025275		Bacteria(100	Proteobacter	Gammaprote	Pseudomona	Pseudomona	Pseudomonas(100)
Otu00015	32	29	12	9	155	0.028309				Bacteria(100	Candidate_d	unclassified(	unclassified(	unclassified(	unclassified(100)
Otu00035	46	11	2	32	150	0.028369		0.013212	0.030418	Bacteria(100	Proteobacter	Alphaproteol	Sphingomon	Sphingomon	Sphingomonas(98)
Otu00121	32	13	21	18	150			0.046448		Bacteria(100	Proteobacter	Alphaproteol	Rhizobiales(1	Rhizobiaceae	Rhizobium(100)
Otu00056	36	15	13	21	149			0.046807		Bacteria(100	Proteobacter	Alphaproteol	Rhizobiales(1	Hyphomicrob	Devosia(77)
Otu00052	39	9	10	31	147			0.01314		Bacteria(100	Proteobacter	Alphaproteol	Sphingomon	Sphingomon	Sphingomonas(100)
Otu00027	38	7	7	41	145			0.005369	0.006571	Bacteria(100	Proteobacter	Alphaproteol	Sphingomon	Sphingomon	Sphingomonas(97)
Otu00025	22	14	19	34	144				0.04005	Bacteria(100	Proteobacter	Betaproteob	Burkholderia	Comamonad	Azohydromonas(59)
Otu00019	3	45	20	8	144	0.022788		0.003298		Bacteria(100	Actinobacter	Actinobacter	Streptomyce	Streptomyce	Streptomyces(74)
Otu00040	31	7	9	45	139			0.033831	0.004245	Bacteria(100	Proteobacter	Alphaproteol	Sphingomon	Sphingomon	Sphingomonas(98)
Otu00068	36	9	12	25	139			0.032071	0.012589	Bacteria(100	Proteobacter	Gammaprote	Xanthomona	Xanthomona	unclassified(100)
Otu00092	18	16	19	33	139		0.042783		0.027354	Bacteria(100	Proteobacter	Betaproteob	Burkholderia	Comamonad	unclassified(98)
Otu00160	32	13	8	30	136			0.034931	0.003585	Bacteria(100	Proteobacter	Alphaproteol	Sphingomon	Sphingomon	Sphingobium(99)
Otu00100	22	21	11	26	134				0.039937	Bacteria(100	Proteobacter	Gammaprote	Xanthomona	Xanthomona	Rhodanobacter(100)
Otu00156	32	3	6	45	127			0.047067	0.006459	Bacteria(100	Bacteroidete	Sphingobacte	Sphingobacte	Chitinophaga	Flavisolibacter(100)
Otu00061	5	14	41	5	125	0.029212			0.007409	Bacteria(100	Actinobacter	Actinobacter	Micromonos	Micromonos	Actinoplanes(90)
Otu00077	26	16	9	20	122				0.007074	Bacteria(100	Proteobacter	Alphaproteol	Rhizobiales(1	Bradyrhizobi	Rhodopseudomonas(62)
Otu00064	26	11	9	29	121				0.004041	Bacteria(100	Proteobacter	Alphaproteol	Sphingomon	Sphingomon	Sphingomonas(98)
Otu00060	27	6	5	43	119			0.032505	0.007914	Bacteria(100	Proteobacter	Alphaproteol	Sphingomon	Sphingomon	Sphingomonas(93)
Otu00054	24	10	8	27	111			0.04411	0.006627	Bacteria(100	Proteobacter	Gammaprote	Xanthomona	Xanthomona	Rhodanobacter(100)
Otu00341	19	27	6	4	108		0.004751			Bacteria(100	Proteobacter	Gammaprote	Pseudomona	Pseudomona	Pseudomonas(84)
Otu00074	27	13	8	10	106		0.025842	0.044224		Bacteria(100	Proteobacter	Alphaproteol	Rhizobiales(1	unclassified	unclassified
Otu00213	28	8	2	29	105				0.040864	Bacteria(100	Proteobacter	Gammaprote	Xanthomona	Xanthomona	Rhodanobacter(99)
Otu00159	10	20	17	8	102				0.040802	Bacteria(100	Proteobacter	Alphaproteol	Rhizobiales(1	Hyphomicrob	Devosia(93)
Otu00070	30	5	5	18	98			0.034812	0.044446	Bacteria(100	Proteobacter	Alphaproteol	Sphingomon	Sphingomon	Sphingomonas(100)
Otu00103	20	5	13	22	98			0.007631		Bacteria(100	Proteobacter	Alphaproteol	Sphingomon	unclassified(8	unclassified(80)
Otu00168	33	5	5	10	96		0.002424	0.00337	0.026332	Bacteria(100	Bacteroidete	Sphingobacte	Sphingobacte	Sphingobacte	Pedobacter(100)
Otu00164	10	8	22	14	94	0.004585			0.039787	Bacteria(100	Proteobacter	Alphaproteol	Caulobactera	Caulobactera	Caulobacter(100)
Otu00104	27	5	3	21	91			0.006273	0.004301	Bacteria(100	Proteobacter	Alphaproteol	Sphingomon	Sphingomon	Sphingomonas(93)
Otu00195	3	11	30	1	89				0.025479	Bacteria(100	Actinobacter	Actinobacter	Streptomyce	Streptomyce	Streptomyces(96)
Otu00181	18	3	9	29	89			0.006201	0.048812	Bacteria(100	Actinobacter	Acidimicrobii	Acidimicrobi	Acidimicrobi	Ilumatobacter(100)
Otu00084	3	27	11	6	88			0.025903		Bacteria(100	Actinobacter	Actinobacter	Streptomyce	Streptomyce	Streptomyces(75)
Otu00175	8	20	10	12	88			0.032598		Bacteria(100	Actinobacter	Actinobacter	Micrococcale	Microbacteri	unclassified(82)
Otu00130	1	25	17	1	87				0.006834	Bacteria(100	Actinobacter	Actinobacter	Micromonos	Micromonos	unclassified(100)
Otu00048	30	2	5	13	87			0.026069		Bacteria(100	Proteobacter	Alphaproteol	Sphingomon	Sphingomon	Sphingomonas(90)
Otu00163	23	1	5	28	86	0.032618		0.003893	0.003082	Bacteria(100	Gemmatimo	Gemmatimo	Gemmatimo	Gemmatimo	Gemmatimonas(100)
Otu00072	28	3	4	16	86			0.025975	0.04092	Bacteria(100	Proteobacter	Alphaproteol	Sphingomon	Sphingomon	Sphingomonas(99)
Otu00109	23	2	5	25	85			0.013067	0.012678	Bacteria(100	Proteobacter	Alphaproteol	Sphingomon	Erythrobacte	Porphyrobacter(88)
Otu00381	4	23	14	3	85			0.003443	0.040419	Bacteria(100	Actinobacter	Actinobacter	Streptomyce	Streptomyce	Streptomyces(98)
Otu00062	19	4	12	15	85			0.047139		Bacteria(100	Proteobacter	Alphaproteol	Sphingomon	Erythrobacte	unclassified(64)
Otu00114	15	8	8	22	84		0.030355	0.044707	0.01251	Bacteria(100	Proteobacter	Alphaproteol	Rhizobiales(1	Xanthobacte	Pseudolabrys(100)
Otu00136	0	13	29	0	84			0.032456	0.014954	Bacteria(100	Actinobacter	Actinobacter	Streptomyce	Streptomyce	unclassified(55)
Otu00255	15	8	7	19	79				0.04092	Bacteria(100	Proteobacter	Gammaprote	Xanthomona	Xanthomona	Dokdonella(100)
Otu00137	16	3	5	30	78			0.012711	0.024871	Bacteria(100	Proteobacter	Alphaproteol	Rhizobiales(1	Rhizobiales_I	Rhizomicrobium(100)
Otu00226	14	5	9	22	78			0.04236		Bacteria(100	Proteobacter	Alphaproteol	Rhodospirilla	unclassified(	unclassified(94)
Otu00093	19	4	2	25	75			0.033613	0.003641	Bacteria(100	Acidobacteri	Acidobacteri	Subgroup_3(	Unknown_Fa	Bryobacter(100)
Otu00090	19	2	5	23	75			0.008229		Bacteria(100	Proteobacter	Alphaproteol	Sphingomon	Sphingomon	Sphingomonas(98)
Otu00236	16	8	9	9	75		0.003839			Bacteria(100	Proteobacter	Alphaproteol	Rhizobiales(1	Rhizobiaceae	Ensifer(70)
Otu00176	19	5	6	14	74			0.042195	0.02391	Bacteria(100	Proteobacter	Gammaprote	Xanthomona	Xanthomona	Luteimonas(91)
Otu00169	19	3	7	16	74			0.042288	0.031119	Bacteria(100	Proteobacter	Alphaproteol	Sphingomon	Sphingomon	Sphingobium(63)
Otu00243	7	20	6	8	74	0.010712		0.029219		Bacteria(100	Actinobacter	Actinobacter	Micrococcale	Micrococcac	Arthrobacter(96)
Otu00110	18	2	6	21	73				0.013038	Bacteria(100	Acidobacteri	Acidobacteri	Subgroup_4(	Unknown_Fa	Blastocatella(100)
Otu00086	21	5	3	14	72			0.026247		Bacteria(100	Proteobacter	Alphaproteol	Sphingomon	Sphingomon	Sphingomonas(100)
Otu00081	21	2	1	23	71			0.003965	0.00262	Bacteria(100	Gemmatimo	Gemmatimo	Gemmatimo	Gemmatimo	unclassified(100)
Otu00249	15	6	3	23	71				0.003986	Bacteria(100	Proteobacter	Alphaproteol	Rhizobiales(9	Rhizobiales_I	Rhizomicrobium(98)

Otu00284	17	4	6	17	71			0.004037		Bacteria(100	Bacteroidete	Cytophagia(1	Cytophagales	Cytophagace	Dyadobacter(100)
Otu00065	4	19	8	7	69			0.041631		Bacteria(100	Proteobacter	Gammaprote	Pseudomona	Pseudomona	Pseudomonas(100)
Otu00548	11	3	6	28	68		0.026078	0.047513	0.012826	Bacteria(100	Nitrospirae(1	Nitrospira(10	Nitrospirales	Nitrospiracea	Nitrospira(100)
Otu00063	2	20	10	3	67	0.041549		0.007366		Bacteria(100	Actinobacter	Actinobacter	Streptomyce	Streptomyce	Streptomyces(66)
Otu00504	11	6	5	22	66		0.045157		0.007858	Bacteria(100	Gemmatimo	Gemmatimo	Gemmatimo	Gemmatimo	unclassified(100)
Otu00144	14	6	8	10	66			0.047719		Bacteria(100	Proteobacter	Alphaproteo	Sphingomon	Sphingomon	Sphingomonas(85)
Otu00083	9	17	6	2	66	0.012605				Bacteria(100	Proteobacter	Gammaprote	Pseudomona	Pseudomona	Pseudomonas(100)
Otu00209	17	5	2	17	65				0.005778	Bacteria(100	Bacteroidete	Sphingobacte	Sphingobacte	Sphingobacte	Mucilaginibacter(100)
Otu00158	13	6	4	19	65				0.043235	Bacteria(100	Proteobacter	Alphaproteo	Sphingomon	WW2-159(93	unclassified(93)
Otu00094	12	6	4	19	63				0.005722	Bacteria(100	Proteobacter	Alphaproteo	Sphingomon	Sphingomon	Sphingomonas(100)
Otu00262	10	9	2	21	63	0.0253			0.032469	Bacteria(100	Proteobacter	Alphaproteo	Rhizobiales(1	Rhizobiales_I	Bauldia(98)
Otu00166	5	15	10	2	62			0.050556	0.006818	Bacteria(100	Actinobacter	Actinobacter	Streptomyce	Streptomyce	Streptomyces(75)
Otu00327	13	4	0	28	62	0.013185			0.006962	Bacteria(100	Gemmatimo	Gemmatimo	Gemmatimo	Gemmatimo	unclassified(100)
Otu00119	17	5	4	10	62		0.011165	0.004258	0.013275	Bacteria(100	Proteobacter	Alphaproteo	Sphingomon	Sphingomon	Sphingomonas(92)
Otu00178	0	21	10	0	62			0.015509	0.048812	Bacteria(100	Actinobacter	Actinobacter	Streptomyce	Streptomyce	unclassified(88)
Otu00322	9	8	11	6	62				0.048812	Bacteria(100	Bacteroidete	Flavobacterii	Flavobacteria	Flavobacteria	Flavobacterium(100)
Otu00286	18	5	2	12	62			0.005731		Bacteria(100	Proteobacter	Alphaproteo	Sphingomon	Sphingomon	Sphingobium(100)
Otu00179	16	5	4	12	62			0.032144		Bacteria(100	Proteobacter	Alphaproteo	Sphingomon	Sphingomon	Sphingobium(98)
Otu00128	17	5	0	17	61			0.032578	0.024967	Bacteria(100	Acidobacteri	Acidobacteri	Subgroup_3(	Unknown_Fa	Bryobacter(100)
Otu00399	2	14	13	3	61			0.007509		Bacteria(100	Actinobacter	Actinobacter	Streptomyce	Streptomyce	Streptomyces(100)
Otu00091	2	16	10	4	60			0.025738	0.04092	Bacteria(100	Actinobacter	Actinobacter	Streptomyce	Streptomyce	Streptomyces(79)
Otu00313	13	9	2	12	60	0.042082				Bacteria(100	Proteobacter	Alphaproteo	Sphingomon	Sphingomon	Sphingomonas(100)
Otu00116	0	13	15	3	59			0.003515	0.012622	Bacteria(100	Actinobacter	Actinobacter	Streptomyce	Streptomyce	Streptomyces(62)
Otu00132	21	3	4	3	59		0.036785	0.041703		Bacteria(100	Candidate_d	unclassified(	unclassified(	unclassified(	unclassified(100)
Otu00330	0	21	8	0	58			0.04411	0.01573	Bacteria(100	Actinobacter	Actinobacter	Streptomyce	Streptomyce	unclassified(94)
Otu00214	14	3	6	12	58			0.006734		Bacteria(100	Proteobacter	Alphaproteo	Rhizobiales(1	Phyllobacteri	Mesorhizobium(63)
Otu00465	10	15	3	2	58	0.025941				Bacteria(100	Proteobacter	Alphaproteo	Rhizobiales(1	Hyphomicrob	Devosia(100)
Otu00088	14	5	5	9	57			0.04236		Bacteria(100	Proteobacter	Alphaproteo	Sphingomon	Sphingomon	Sphingomonas(97)
Otu00097	2	14	11	2	56			0.004185		Bacteria(100	Actinobacter	Actinobacter	Streptomyce	Streptomyce	Streptomyces(69)
Otu00238	13	3	2	19	55				0.012865	Bacteria(100	Candidate_d	unclassified(	unclassified(	unclassified(	unclassified(100)
Otu00294	14	3	3	14	54	0		0.041997	0.007297	Bacteria(100	Proteobacter	Alphaproteo	Sphingomon	Sphingomon	Sphingomonas(71)
Otu00247	11	1	1	28	54				0.025084	Bacteria(100	Chloroflexi(1	KD4-96(100)	unclassified(	unclassified(	unclassified(100)
Otu00323	17	7	0	6	54				0.027639	Bacteria(100	Bacteroidete	Sphingobacte	Sphingobacte	Sphingobacte	unclassified(97)
Otu00216	15	4	2	12	54			0.006903	0.040179	Bacteria(100	Proteobacter	Alphaproteo	Rhodospirilla	Rhodospirilla	Reyranella(100)
Otu00251	11	5	8	6	54			0.042742		Bacteria(100	Proteobacter	Alphaproteo	Rhizobiales(1	unclassified(	unclassified(100)
Otu00143	13	2	4	15	53			0.043874		Bacteria(100	Chloroflexi(1	Chloroflexia(	Chloroflexale	Roseiflexacea	Roseiflexus(100)
Otu00212	14	4	1	14	52			0.034459	0.003361	Bacteria(100	Proteobacter	Betaproteob	SC-I-84(100)	unclassified(	unclassified(100)
Otu00105	10	7	2	14	52				0.007521	Bacteria(100	Proteobacter	Alphaproteo	Sphingomon	Sphingomon	Sphingomonas(97)
Otu00145	13	3	3	14	52				0.040553	Bacteria(100	Proteobacter	Alphaproteo	Sphingomon	Sphingomon	Sphingomonas(82)
Otu00118	2	12	12	0	52			0.041776	0.044223	Bacteria(100	Actinobacter	Actinobacter	Micromonos	Micromonos	unclassified(89)
Otu00306	9	2	5	20	52			0.027867		Bacteria(100	Acidobacteri	Acidobacteri	Acidobacteri	Acidobacteri	unclassified(100)
Otu00125	15	3	2	12	52			0.029292		Bacteria(100	Proteobacter	Alphaproteo	Caulobactera	Caulobactera	Phenylobacterium(85)
Otu00122	14	3	1	15	51			0.015128	0.002732	Bacteria(100	Proteobacter	Alphaproteo	Sphingomon	unclassified(	unclassified(64)
Otu00227	1	4	20	1	51	0.004866			0.00349	Bacteria(100	Proteobacter	Betaproteob	Methylophila	Methylophila	Methylophilus(97)
Otu00279	0	11	14	1	51			0.015056		Bacteria(100	Actinobacter	Actinobacter	Micromonos	Micromonos	unclassified(100)
Otu00587	0	4	21	0	50	0.010682		0.016515	0.006555	Bacteria(100	Proteobacter	Gammaprote	Pseudomona	Pseudomona	Pseudomonas(100)
Otu00575	3	7	13	4	50				0.040746	Bacteria(100	Actinobacter	Actinobacter	Micrococcale	Microbacteri	Lysinimonas(86)
Otu00875	4	1	12	16	50		0.02673			Bacteria(100	Acidobacteri	Acidobacteri	Subgroup_4(	RB41(100)	unclassified(100)
Otu00276	9	2	4	19	49				0.007632	Bacteria(100	Acidobacteri	Acidobacteri	Subgroup_3(	Unknown_Fa	Bryobacter(100)
Otu00329	11	2	1	21	49			0.015581	0.031484	Bacteria(100	Bacteroidete	Sphingobacte	Sphingobacte	Chitinophaga	unclassified(100)
Otu00250	15	1	3	11	49			0.025467	0.032875	Bacteria(100	Proteobacter	Alphaproteo	Rhizobiales(1	Phyllobacteri	unclassified(80)
Otu00452	13	0	4	14	48	0.013185				Bacteria(100	Candidate_d	unclassified(	unclassified(	unclassified(	unclassified(100)
Otu00421	3	12	7	3	47			0.046573		Bacteria(100	Actinobacter	Actinobacter	Micromonos	Micromonos	unclassified(100)
Otu00228	4	13	6	0	46				0.027639	Bacteria(100	Actinobacter	Actinobacter	Streptomyce	Streptomyce	unclassified(92)
Otu00126	3	12	7	2	46	0.048612		0		Bacteria(100	Actinobacter	Actinobacter	Micromonos	Micromonos	unclassified(96)
Otu00201	10	2	3	15	45				0.00561	Bacteria(100	Gemmatimo	Gemmatimo	Gemmatimo	Gemmatimo	Gemmatimonas(100)
Otu00278	11	3	3	11	45			0.024742	0.02391	Bacteria(100	Proteobacter	Alphaproteo	Rhizobiales(1	Xanthobacte	Pseudolabrys(100)
Otu00234	2	6	13	3	45				0.025159	Bacteria(100	Proteobacter	Betaproteob	Burkholderia	Comamonad	Acidovorax(92)
Otu00113	5	15	1	3	45	0.003732		0.032959		Bacteria(100	Proteobacter	Gammaprote	Pseudomona	Pseudomona	Pseudomonas(100)
Otu00202	3	9	9	2	44			0.029292	0.026531	Bacteria(100	Actinobacter	Actinobacter	Micromonos	Micromonos	unclassified(74)
Otu00277	13	0	3	12	44			0.012761		Bacteria(100	Acidobacteri	Acidobacteri	Subgroup_6(	unclassified(	unclassified(100)
Otu00378	5	3	11	6	44	0.003559				Bacteria(100	Proteobacter	Alphaproteo	Caulobactera	Caulobactera	Asticcacaulis(100)
Otu00479	8	4	2	15	43				0.006004	Bacteria(100	Acidobacteri	Acidobacteri	Subgroup_3(	SJA-149(100)	unclassified(100)
Otu00149	14	0	0	15	43				0.013038	Bacteria(100	Acidobacteri	Acidobacteri	Subgroup_6(	unclassified(	unclassified(100)
Otu00135	1	15	5	1	43	0.028831		0.013501	0.033997	Bacteria(100	Actinobacter	Actinobacter	Streptomyce	Streptomyce	unclassified(69)
Otu01327	15	2	0	9	43			0.005731		Bacteria(100	Candidate_d	unclassified(	unclassified(	unclassified(	unclassified(100)
Otu00390	3	2	15	2	42	0.004498			0.006004	Bacteria(100	Proteobacter	Betaproteob	Burkholderia	Comamonad	Pelomonas(78)
Otu00444	0	5	16	0	42				0.012921	Bacteria(100	Cyanobacteri	unclassified(7	unclassified(7	unclassified(7	unclassified(72)
Otu00199	0	15	6	0	42			0.032313	0.027639	Bacteria(100	Actinobacter	Actinobacter	unclassified(5	unclassified(5	unclassified(58)
Otu00289	0	3	18	0	42				0.033868	Bacteria(100	Gemmatimo	Gemmatimo	Gemmatimo	Gemmatimo	unclassified(91)
Otu00151	13	3	2	6	42			0.025666		Bacteria(100	Proteobacter	Alphaproteo	Sphingomon	Sphingomon	Sphingomonas(96)
Otu00471	2	14	4	2	42	0.041308		0.026267		Bacteria(100	Bacteroidete	Sphingobacte	Sphingobacte	Chitinophaga	Niastella(100)
Otu00615	10	3	6	4	42		0.011794	0.027788		Bacteria(100	Actinobacter	Thermoleoph	Gaiellales(10	unclassified(	unclassified(100)
Otu00299	13	3	2	6	42			0.032886		Bacteria(100	Proteobacter	Alphaproteo	Sphingomon	Sphingomon	Novosphingobium(59)
Otu00146	13	6	1	2	42	0.00557				Bacteria(100	Candidate_d	unclassified(	unclassified(	unclassified(	unclassified(100)
Otu00745	9	7	3	3	41				0	Bacteria(100	Proteobacter	Alphaproteo	Caulobactera	Caulobactera	Brevundimonas(100)
Otu00193	0	10	9	3	41		0	0.00366	0.027639	Bacteria(100	Actinobacter	Actinobacter	Streptomyce	Streptomyce	Streptomyces(78)
Otu00161	1	8	11	0	40			0.033106	0.007074	Bacteria(100	Actinobacter	Actinobacter	Micromonos	Micromonos	unclassified(85)
Otu00591	8	1	5	12	40			0.033106	0.043291	Bacteria(100	Gemmatimo	Gemmatimo	Gemmatimo	Gemmatimo	Gemmatimonas(100)
Otu00157	7	6	3	8	40				0.048812	Bacteria(100	Proteobacter	Alphaproteo	Rhizobiales(1	Rhizobiaceae	Rhizobium(100)
Otu00332	10	2	6	4	40			0.041477		Bacteria(100	Proteobacter	Alphaproteo	Sphingomon	Sphingomon	Sphingopyxis(96)
Otu00484	7	1	4	15	39		0.015426	0.013656	0.007297	Bacteria(100	Acidobacteri	Acidobacteri	Subgroup_4(	Unknown_Fa	Blastocatella(100)
Otu00269	0	2	17	1	39	0.031446			0.031636	Bacteria(100	Actinobacter	Actinobacter	Streptomyce	Streptomyce	unclassified
Otu00519	8	7	1	7	39	0.010885			0.04092	Bacteria(100	Proteobacter	Betaproteob	Methylophila	Methylophila	unclassified(96)
Otu00266	11	2	1	11	39			0.015581	0.043082	Bacteria(100	Proteobacter	Alphaproteo	Rhizobiales(1	Rhizobiales_I	Rhizomicrobium(100)
Otu00371	9	2	4	9	39			0.044707		Bacteria(100	Proteobacter	Alphaproteo	Caulobactera	Caulobactera	Caulobacter(88)
Otu00263	12	0	1	12	38				0.007074	Bacteria(100	Acidobacteri	Acidobacteri	Subgroup_6(	unclassified(	unclassified(100)
Otu00189	13	1	1	8	38				0.031652	Bacteria(100	Proteobacter	Alphaproteo	Sphingomon	Sphingomon	Sphingomonas(100)
Otu00273	1	9	8	2	38			0.015907		Bacteria(100	Actinobacter	Actinobacter	Streptomyce	Streptomyce	Streptomyces(65)
Otu00173	7	8	1	5	37	0.009897			0.033997	Bacteria(100	Candidate_d	unclassified(	unclassified(	unclassified(	unclassified(100)
Otu00170	8	1	5	9	37			0.033106		Bacteria(100	Proteobacter	Alphaproteo	Sphingomon	Ellin6055(53)	unclassified(53)
Otu00267	0	3	15	0	36	0.029841			0.013206	Bacteria(100	Cyanobacteri	unclassified(	unclassified(	unclassified(	unclassified(100)
Otu00361	3	4	3	16	36		0.042333		0.03079	Bacteria(100	Gemmatimo	Gemmatimo	Gemmatimo	Gemmatimo	Gemmatimonas(100)
Otu00682	0	7	11	0	36				0.032649	Bacteria(100	Cyanobacteri	unclassified(8	unclassified(8	unclassified(8	unclassified(89)
Otu00230	2	4	12	0	36				0.044327	Bacteria(100	Proteobacter	Alphaproteo	Rickettsiales(	mitochondria	unclassified(100)
Otu01985	4	0	7	14	36	0.003819		0.016515		Bacteria(100	Bacteroidete	Cytophagia(1	Cytophagales	Cytophagace	Emticicia(100)
Otu00290	9	0	5	8	36	0.006405		0.034604		Bacteria(100	Chloroflexi(1	Anaerolineae	Anaerolineal	Anaerolineac	unclassified(100)
Otu00366	1	9	7	2	36			0.047296		Bacteria(100	Proteobacter	Gammaprote	Pseudomona	Pseudomona	Pseudomonas(100)
Otu00182	3	13	1	2	36	0.031701				Bacteria(100	Proteobacter	Gammaprote	Pseudomona	Pseudomona	Pseudomonas(100)
Otu00400	1	4	12	1	35	0.044375			0.007228	Bacteria(100	Actinobacter	Actinobacter	Streptomyce	Streptomyce	Streptomyces(95)
Otu00364	8	2	1	13	35				0.007613	Bacteria(100	Gemmatimo	Gemmatimo	Gemmatimo	Gemmatimo	Gemmatimonas(100)
Otu00838	9	1	1	13	35			0.015907	0.033754	Bacteria(100	Candidate_d	unclassified(	unclassified(	unclassified(	unclassified(100)

Otu00190	7	5	5	1	35				0.033997	Bacteria(100	Proteobacter	Alphaproteol	Sphingomona	unclassified(	unclassified(67)
Otu00191	2	8	7	1	35				0.04092	Bacteria(100	Actinobacter	Actinobacter	Micromonos	Micromonos	unclassified(89)
Otu00171	2	8	5	5	35			0.013656		Bacteria(100	Actinobacter	Actinobacter	Micromonos	Micromonos	unclassified(100)
Otu00205	6	7	3	3	35		0			Bacteria(100	Proteobacter	Alphaproteol	Rhizobiales(1	Rhizobiaceae	Rhizobium(74)
Otu00386	7	6	1	7	35	0.00557				Bacteria(100	Bacteroidete	Sphingobact	Sphingobact	Sphingobact	Pedobacter(100)
Otu00312	3	9	5	0	34		0		0.009016	Bacteria(100	Actinobacter	Actinobacter	Micromonos	Micromonos	unclassified(100)
Otu00357	7	2	3	10	34				0.026463	Bacteria(100	Proteobacter	Alphaproteol	Rhizobiales(1	Xanthobacte	unclassified(78)
Otu00401	5	3	2	14	34				0.03094	Bacteria(100	Proteobacter	Gammaprote	Xanthomona	Xanthomona	Rhodanobacter(71)
Otu00172	12	1	1	6	34			0.006734		Bacteria(100	Proteobacter	Alphaproteol	Sphingomona	unclassified(	unclassified(70)
Otu00222	10	2	3	4	34		0.043143	0.035376		Bacteria(100	Proteobacter	Alphaproteol	Sphingomona	Sphingomona	Sphingomonas(100)
Otu00271	9	1	1	12	34			0.047296		Bacteria(100	Chloroflexi(1	KD4-96(100)	unclassified(	unclassified(	unclassified(100)
Otu00253	8	3	2	8	34			0.050556		Bacteria(100	Proteobacter	Betaproteob	Nitrosomona	Nitrosomona	unclassified(77)
Otu00246	8	4	4	2	34		0.043143			Bacteria(100	Proteobacter	Alphaproteol	Rhizobiales(1	unclassified(	unclassified(89)
Otu00891	8	3	3	6	34	0				Bacteria(100	Proteobacter	Alphaproteol	Caulobactera	Caulobactera	Asticcacaulis(100)
Otu00298	4	1	8	8	34	0.009897				Bacteria(100	Chloroflexi(1	Chloroflexia(	Chloroflexale	Roseiflexace	Roseiflexus(100)
Otu00206	2	7	7	1	33			0.026652	0.013275	Bacteria(100	Actinobacter	Actinobacter	Streptomyce	Streptomyce	Streptomyces(59)
Otu00515	8	3	2	7	33				0.026051	Bacteria(100	Chloroflexi(1	JG30-KF-CM6	unclassified(	unclassified(	unclassified(100)
Otu00197	2	8	6	1	33				0.045598	Bacteria(100	Actinobacter	Actinobacter	Streptomyce	Streptomyce	Streptomyces(53)
Otu00340	13	1	1	3	33		0.026138	0.007414		Bacteria(100	Acidobacteri	Acidobacteri	Subgroup_3(	Unknown_Fa	Bryobacter(100)
Otu00307	13	1	1	3	33		0.026459	0.007509		Bacteria(100	Acidobacteri	Holophagae(	Subgroup_10	ABS-19(100)	unclassified(100)
Otu00203	0	9	7	1	33			0.007703		Bacteria(100	Actinobacter	Actinobacter	Streptomyce	Streptomyce	Streptomyces(77)
Otu00449	12	2	1	3	33		0.047013	0.008355		Bacteria(100	Candidate_d	unclassified(	unclassified(	unclassified(	unclassified(100)
Otu01059	5	1	6	9	33			0.035376		Bacteria(100	Candidate_d	unclassified(	unclassified(	unclassified(	unclassified(100)
Otu00408	7	0	4	11	33			0.044707		Bacteria(100	Chloroflexi(1	JG30-KF-CM6	unclassified(	unclassified(	unclassified(100)
Otu00350	0	5	11	0	32				0.014676	Bacteria(100	Actinobacter	Actinobacter	Micromonos	Micromonos	unclassified(75)
Otu00300	1	8	7	0	32			0.012413	0.043375	Bacteria(100	Actinobacter	Actinobacter	Streptomyce	Streptomyce	unclassified
Otu00239	9	2	2	6	32			0.027867		Bacteria(100	Proteobacter	Alphaproteol	Sphingomona	Sphingomona	Sphingomonas(100)
Otu00342	7	1	2	12	32			0.042742		Bacteria(100	Acidobacteri	Acidobacteri	Subgroup_3(	Unknown_Fa	Bryobacter(96)
Otu00333	9	1	3	6	32			0.047296		Bacteria(100	Acidobacteri	Acidobacteri	Subgroup_6(	unclassified(	unclassified(100)
Otu00605	9	1	2	7	31				0.026051	Bacteria(100	Bacteroidete	Sphingobact	Sphingobact	Chitinophaga	Flavisolibacter(100)
Otu00229	0	3	5	15	31		0.002744		0.03154	Bacteria(100	Actinobacter	Actinobacter	Propionibact	Nocardioidac	Aeromicrobium(100)
Otu00232	9	2	0	9	31			0.027867	0.033411	Bacteria(100	Proteobacter	Deltaproteot	GR-WP33-30	unclassified(	unclassified(100)
Otu00233	4	7	1	7	31				0.04092	Bacteria(100	Proteobacter	Alphaproteol	Rhizobiales(1	Phyllobacteri	Mesorhizobium(58)
Otu00180	8	2	2	7	31			0.013656		Bacteria(100	Chloroflexi(1	TK10(100)	unclassified(	unclassified(	unclassified(100)
Otu00581	6	3	2	8	30				0.013275	Bacteria(100	Gemmatimo	Gemmatimo	Gemmatimo	Gemmatimo	unclassified(100)
Otu00472	3	1	5	12	30	0.032618	0.047013		0.026425	Bacteria(100	Chloroflexi(1	Anaerolineae	Anaerolinea	Anaerolinea	unclassified(100)
Otu00430	11	1	0	6	30				0.027639	Bacteria(100	Proteobacter	Alphaproteol	Sphingomona	Sphingomona	unclassified(84)
Otu00490	3	2	2	16	30		0.036515		0.030585	Bacteria(100	Proteobacter	Alphaproteol	unclassified(	unclassified(	unclassified(100)
Otu00305	9	1	1	8	30			0.004402	0.031652	Bacteria(100	Proteobacter	Alphaproteol	Rhodospirilla	Rhodospirilla	Reyranella(100)
Otu00922	5	4	2	8	30				0.04092	Bacteria(100	Proteobacter	Alphaproteol	Rhizobiales(1	Phyllobacteri	Mesorhizobium(85)
Otu00302	0	3	12	0	30				0.044327	Bacteria(100	unclassified(	unclassified(	unclassified(	unclassified(	unclassified(94)
Otu00283	0	4	11	0	30			0.016515	0.045542	Bacteria(100	Cyanobacteri	unclassified(	unclassified(	unclassified(	unclassified(100)
Otu00743	10	1	1	6	30				0.045598	Bacteria(100	Candidate_d	unclassified(	unclassified(	unclassified(	unclassified(100)
Otu00215	0	9	5	2	30			0.007703		Bacteria(100	Actinobacter	Actinobacter	Streptomyce	Streptomyce	Streptomyces(75)
Otu01070	11	1	2	2	30		0.034063	0.013357		Bacteria(100	Candidate_d	unclassified(	unclassified(	unclassified(	unclassified(100)
Otu00645	8	0	4	6	30			0.033903		Bacteria(100	Cyanobacteri	ML635J-21(1	unclassified(	unclassified(	unclassified(100)
Otu00358	10	2	0	6	30			0.041477		Bacteria(100	Bacteroidete	Cytophagia(1	Cytophagales	Cytophagace	Ohtaekwangia(100)
Otu00355	1	6	6	4	30			0.048221		Bacteria(100	Actinobacter	Actinobacter	Micrococcale	Intrasporang	Phycicoccus(100)
Otu01366	5	1	2	13	29		0.004252	0.035376	0.00393	Bacteria(100	Proteobacter	Alphaproteol	Rhodospirilla	AKYH478(10	unclassified(100)
Otu00732	9	1	0	9	29				0.007747	Bacteria(100	Acidobacteri	Acidobacteri	Subgroup_3(	Unknown_Fa	Bryobacter(100)
Otu00989	7	2	0	11	29			0.026652	0.032595	Bacteria(100	Chloroflexi(1	Chloroflexia(	Chloroflexale	Roseiflexace	Roseiflexus(100)
Otu00315	9	0	0	11	29			0.007703	0.0404	Bacteria(100	Proteobacter	Alphaproteol	Sphingomona	Sphingomona	Sphingomonas(100)
Otu00404	2	6	6	1	29				0.045598	Bacteria(100	Actinobacter	Actinobacter	Micromonos	Micromonos	unclassified(80)
Otu00188	7	1	3	7	29			0.013656		Bacteria(100	Proteobacter	Alphaproteol	Sphingomona	Sphingomona	Sphingomonas(100)
Otu00407	10	2	1	3	29		0.045334			Bacteria(100	Proteobacter	Betaproteob	Nitrosomona	Nitrosomona	unclassified(100)
Otu01179	4	2	7	3	29	0.024004				Bacteria(100	Proteobacter	Alphaproteol	Caulobactera	Caulobactera	Asticcacaulis(100)
Otu00288	4	9	1	1	29	0.043589				Bacteria(100	Actinobacter	Actinobacter	Micromonos	Micromonos	unclassified(94)
Otu00242	6	2	3	6	28				0	Bacteria(100	Proteobacter	Alphaproteol	Sphingomona	unclassified(	unclassified(83)
Otu00599	0	4	10	0	28				0.003226	Bacteria(100	Cyanobacteri	unclassified(	unclassified(	unclassified(	unclassified(100)
Otu00593	1	9	4	0	28			0.015907	0.01573	Bacteria(100	Actinobacter	Actinobacter	Micromonos	Micromonos	unclassified(100)
Otu00701	5	2	1	12	28				0.027528	Bacteria(100	Proteobacter	Alphaproteol	Rhizobiales(1	DUNssu371(	unclassified(100)
Otu00532	0	5	9	0	28			0.009517	0.033411	Bacteria(100	unclassified(	unclassified(	unclassified(	unclassified(	unclassified(58)
Otu00483	7	1	2	8	28				0.04092	Bacteria(100	Proteobacter	Alphaproteol	Rhodospirilla	Rhodospirilla	Reyranella(100)
Otu00301	9	1	1	6	28				0.045598	Bacteria(100	Proteobacter	Alphaproteol	Sphingomona	Sphingomona	Sphingomonas(95)
Otu00602	10	0	0	8	28			0.009517		Bacteria(100	Bacteroidete	Cytophagia(1	Cytophagales	Cytophagace	Ohtaekwangia(100)
Otu00638	9	2	0	6	28			0.027867		Bacteria(100	Proteobacter	Alphaproteol	Sphingomona	Sphingomona	Sphingomonas(89)
Otu00735	8	0	2	8	28			0.033903		Bacteria(100	Candidate_d	unclassified(	unclassified(	unclassified(	unclassified(100)
Otu00881	0	4	8	4	28		0.015426			Bacteria(100	Chloroflexi(1	Thermomicro	JG30-KF-CM4	unclassified(	unclassified(100)
Otu03157	8	2	0	7	27				0.004504	Bacteria(100	Acidobacteri	Acidobacteri	Subgroup_4(	Unknown_Fa	Blastocatella(100)
Otu00422	6	2	1	9	27				0.015228	Bacteria(100	Gemmatimo	Gemmatimo	Gemmatimo	Gemmatimo	unclassified(100)
Otu00818	7	1	2	7	27				0.026051	Bacteria(100	Bacteroidete	Sphingobact	Sphingobact	env.OPS_17(	unclassified(100)
Otu00418	4	1	2	13	27				0.033698	Bacteria(100	Acidobacteri	Acidobacteri	Subgroup_6(	unclassified(	unclassified(100)
Otu00344	1	7	5	1	27				0.033997	Bacteria(100	Actinobacter	Actinobacter	Micromonos	Micromonos	unclassified(100)
Otu00224	9	2	0	5	27			0.027867		Bacteria(100	Proteobacter	Alphaproteol	Sphingomona	Sphingomona	unclassified(63)
Otu00520	6	1	2	8	26				0.013275	Bacteria(100	Chloroflexi(1	Chloroflexia(	Chloroflexale	Roseiflexace	Roseiflexus(100)
Otu00142	3	1	1	16	26		0.042333		0.015117	Bacteria(100	Acidobacteri	Acidobacteri	Subgroup_6(	unclassified(	unclassified(100)
Otu00265	5	3	2	6	26				0.02391	Bacteria(100	Proteobacter	Alphaproteol	Sphingomona	unclassified	unclassified
Otu00585	8	0	1	8	26				0.031652	Bacteria(100	unclassified(	unclassified(	unclassified(	unclassified(	unclassified(100)
Otu00596	10	0	1	4	26			0.003738		Bacteria(100	Proteobacter	Alphaproteol	Sphingomona	Sphingomona	Sphingomonas(94)
Otu00292	0	9	3	2	26			0.007703		Bacteria(100	Actinobacter	Actinobacter	Streptomyce	Streptomyce	Streptomyces(65)
Otu00766	7	2	1	6	26			0.026652		Bacteria(100	Proteobacter	Alphaproteol	Sphingomona	Erythrobacte	Altererythrobacter(94)
Otu00513	6	0	4	6	26	0.013185		0.029292		Bacteria(100	Proteobacter	Alphaproteol	Sphingomona	unclassified(	unclassified(94)
Otu00311	8	1	1	6	26			0.033106		Bacteria(100	Proteobacter	Alphaproteol	Sphingomona	Sphingomona	Sphingomonas(94)
Otu00831	6	1	2	8	26			0.048221		Bacteria(100	Candidate_d	unclassified(	unclassified(	unclassified(	unclassified(100)
Otu01127	7	4	2	0	26		0.029492			Bacteria(100	Proteobacter	Alphaproteol	Sphingomona	Sphingomona	Sphingobium(93)
Otu00806	0	0	11	4	26	0.004926				Bacteria(100	Proteobacter	Betaproteob	Methylophila	Methylophila	Methylophilus(100)
Otu01097	6	5	1	2	26	0.032618				Bacteria(100	Bacteroidete	Flavobacterii	Flavobacteria	Flavobacteria	Flavobacterium(100)
Otu00392	4	2	2	9	25				0.004504	Bacteria(100	Proteobacter	Gammaprote	Xanthomona	Xanthomona	unclassified(89)
Otu00337	0	4	8	1	25				0.012279	Bacteria(100	Actinobacter	Actinobacter	Streptomyce	Streptomyce	Streptomyces(77)
Otu00237	7	1	1	7	25			0.013656	0.013275	Bacteria(100	Proteobacter	Alphaproteol	Sphingomona	Sphingomona	Sphingobium(82)
Otu00830	6	1	0	11	25			0.008355	0.032759	Bacteria(100	Gemmatimo	Gemmatimo	Gemmatimo	Gemmatimo	Gemmatimonas(100)
Otu00561	4	2	6	1	25				0.045598	Bacteria(100	Chloroflexi(1	Anaerolineae	Anaerolinea	Anaerolinea	unclassified(100)
Otu00223	7	0	2	7	25			0.004565		Bacteria(100	Proteobacter	Alphaproteol	Sphingomona	Sphingomona	Sphingomonas(94)
Otu00969	8	1	2	3	25			0.012413		Bacteria(100	Bacteroidete	Sphingobact	Sphingobact	Sphingobact	Arcticibacter(100)
Otu00373	6	0	2	9	25			0.029292		Bacteria(100	Proteobacter	Betaproteob	Burkholderia	Comamonad	Ramlibacter(65)
Otu00763	7	0	3	5	25			0.044707		Bacteria(100	Proteobacter	Betaproteob	unclassified(	unclassified(	unclassified(80)
Otu00583	6	1	4	3	25			0.048221		Bacteria(100	Proteobacter	Alphaproteol	Rhizobiales(1	Hyphomicrob	Devosia(93)
Otu01416	7	4	1	1	25		0.043143			Bacteria(100	Proteobacter	Alphaproteol	Rhizobiales(1	Phyllobacteri	unclassified(85)
Otu00431	2	0	7	7	25	0.003819				Bacteria(100	Proteobacter	Betaproteob	Burkholderia	Comamonad	Acidovorax(82)
Otu00887	6	0	5	3	25	0.048612				Bacteria(100	Actinobacter	Acidimicrobii	Acidimicrobi	OM1_clade(1	unclassified(100)
Otu00394	5	1	0	12	24		0.0454		0.005555	Bacteria(100	Proteobacter	Alphaproteol	Sphingomona	Sphingomona	Sphingomonas(100)
Otu00335	6	0	1	10	24				0.006271	Bacteria(100	Proteobacter	Deltaproteot	Myxococcale	Phaselicystid	Phaselicystis(89)

Otu00721	5	3	1	6	24				0.00797	Bacteria(100	Actinobacter	Acidimicrobii	Acidimicrobi	OM1_clade(1	unclassified(100)
Otu00582	0	8	4	0	24				0.01573	Bacteria(100	Actinobacter	Actinobacter	Streptomyce	Streptomyce	unclassified(59)
Otu00498	0	6	6	0	24				0.027639	Bacteria(100	Cyanobacter	unclassified(9	unclassified(9	unclassified(9	unclassified(92)
Otu00674	5	1	0	12	24				0.044223	Bacteria(100	Chloroflexi(1	Ktedonobact	JG30-KF-AS9	unclassified(1	unclassified(100)
Otu00613	5	0	4	6	24			0.009517		Bacteria(100	SHA-109(100	unclassified(1	unclassified(1	unclassified(1	unclassified(100)
Otu00954	9	1	0	4	24			0.015907		Bacteria(100	Proteobacter	Alphaproteol	Rhodospirilla	unclassified(8	unclassified(86)
Otu00759	6	2	2	4	24			0.024742		Bacteria(100	Proteobacter	Alphaproteol	Rhizobiales(1	unclassified(1	unclassified(100)
Otu00374	7	2	1	4	24			0.026652		Bacteria(100	Proteobacter	Alphaproteol	Sphingomona	Sphingomona	Novosphingobium(79)
Otu00530	1	8	3	0	24			0.033106		Bacteria(100	Actinobacter	Actinobacter	Streptomyce	Streptomyce	Streptomyces(100)
Otu00348	0	3	6	6	24		0.030672			Bacteria(100	Chloroflexi(1	Chloroflexia(	Herpetosipho	Herpetosipho	Herpetosiphon(100)
Otu00978	3	1	6	4	24	0.045288				Bacteria(100	Actinobacter	Actinobacter	Propionibact	Nocardioidac	Nocardioides(100)
Otu00445	5	2	1	7	23				0.013275	Bacteria(100	Proteobacter	Alphaproteol	Sphingomona	Sphingomona	Novosphingobium(67)
Otu00554	5	1	0	11	23				0.014676	Bacteria(100	Proteobacter	Deltaproteob	Myxococcale	Haliangiacea	Haliangium(100)
Otu00317	6	0	1	9	23			0.029292	0.015228	Bacteria(100	Chloroflexi(1	KD4-96(100)	unclassified(1	unclassified(1	unclassified(100)
Otu00837	0	1	10	1	23	0.028369			0.031174	Bacteria(100	Proteobacter	Betaproteob	Methylophila	Methylophila	Methylotenera(100)
Otu00346	7	1	1	5	23				0.033997	Bacteria(100	Chloroflexi(1	Ktedonobact	JG30-KF-AS9	unclassified(1	unclassified(100)
Otu00275	7	1	1	5	23				0.033997	Bacteria(100	Proteobacter	Alphaproteol	Sphingomona	Sphingomona	Sphingomonas(93)
Otu00409	7	1	1	5	23			0.013656		Bacteria(100	Gemmatimo	Gemmatimo	Gemmatimo	Gemmatimo	unclassified(100)
Otu00706	5	1	3	5	23			0.035376		Bacteria(100	Acidobacteri	Acidobacteri	Subgroup_4(	RB41(100)	unclassified(100)
Otu00473	6	1	1	7	23			0.048221		Bacteria(100	Acidobacteri	Acidobacteri	Subgroup_4(	Unknown_Fa	Blastocatella(100)
Otu00726	0	5	5	3	23		0	0.050556		Bacteria(100	Actinobacter	Actinobacter	Micrococcale	Microbacteri	Leifsonia(100)
Otu01033	5	0	1	11	23			0.050556		Bacteria(100	Chloroflexi(1	TK10(100)	unclassified(1	unclassified(1	unclassified(100)
Otu01430	5	1	1	8	22				0.012279	Bacteria(100	Gemmatimo	Gemmatimo	Gemmatimo	Gemmatimo	unclassified(100)
Otu00417	5	1	0	10	22				0.025297	Bacteria(100	Bacteroidete	Sphingobact	Sphingobact	Chitinophaga	Flavisolibacter(100)
Otu01091	8	0	0	6	22				0.027639	Bacteria(100	Gemmatimo	Gemmatimo	Gemmatimo	Gemmatimo	Gemmatimonas(100)
Otu00343	6	0	1	8	22			0.029292	0.031652	Bacteria(100	Acidobacteri	Acidobacteri	Subgroup_6(	unclassified(1	unclassified(100)
Otu00619	5	1	1	8	22				0.031652	Bacteria(100	Candidate_d	unclassified(1	unclassified(1	unclassified(1	unclassified(100)
Otu00372	7	1	1	4	22			0.013656		Bacteria(100	Proteobacter	Alphaproteol	Sphingomona	Sphingomona	Sphingomonas(100)
Otu01050	9	1	0	2	22		0.029492	0.015907		Bacteria(100	Candidate_d	unclassified(1	unclassified(1	unclassified(1	unclassified(100)
Otu00909	8	1	1	2	22			0.033106		Bacteria(100	Candidate_d	unclassified(1	unclassified(1	unclassified(1	unclassified(100)
Otu00414	0	8	1	4	22			0.033903		Bacteria(100	Actinobacter	Actinobacter	Micromonos	Micromonos	unclassified(70)
Otu00696	9	0	2	0	22		0.036183	0.034531		Bacteria(100	Bacteroidete	Cytophagia(1	Cytophagales	Cytophagace	Pontibacter(100)
Otu00843	7	1	1	4	22			0.042742		Bacteria(100	Gemmatimo	Gemmatimo	Gemmatimo	Gemmatimo	unclassified(100)
Otu01066	7	0	1	6	22			0.044707		Bacteria(100	Proteobacter	Alphaproteol	Rhizobiales(1	Methylobact	Microvirga(100)
Otu01212	6	1	2	4	22			0.048221		Bacteria(100	Proteobacter	Deltaproteol	Bdellovibrion	Bdellovibrion	OM27_clade(100)
Otu00755	0	5	5	2	22			0.050556		Bacteria(100	Actinobacter	Actinobacter	Propionibact	Nocardioidac	Nocardioides(100)
Otu00559	3	1	5	4	22	0.032618				Bacteria(100	Actinobacter	Actinobacter	unclassified(7	unclassified(7	unclassified(77)
Otu00753	6	2	0	5	21			0.024742	0.009016	Bacteria(100	Proteobacter	Betaproteob	Methylophila	Methylophila	Methylobacillus(62)
Otu00916	7	1	0	5	21				0.009016	Bacteria(100	Proteobacter	Alphaproteol	Rhizobiales(1	Rhizobiales_I	Rhizomicrobium(100)
Otu00642	1	1	8	1	21	0.009897			0.012279	Bacteria(100	Actinobacter	Actinobacter	Micromonos	Micromonos	unclassified(55)
Otu00767	5	1	1	7	21			0.035376	0.013275	Bacteria(100	Bacteroidete	Sphingobact	Sphingobact	env.OPS_17(	unclassified(100)
Otu00338	4	1	0	11	21				0.014676	Bacteria(100	Proteobacter	Alphaproteol	Rhodospirilla	Rhodospirilla	Reyranella(100)
Otu00244	5	0	1	9	21				0.015228	Bacteria(100	Acidobacteri	Acidobacteri	Subgroup_6(	unclassified(1	unclassified(100)
Otu00608	4	1	5	1	21	0.032618			0.033997	Bacteria(100	Proteobacter	Betaproteob	Burkholderia	Comamonad	Acidovorax(100)
Otu00349	4	3	1	5	21				0.033997	Bacteria(100	Gemmatimo	Gemmatimo	Gemmatimo	Gemmatimo	unclassified(100)
Otu00833	2	3	5	1	21				0.033997	Bacteria(100	Proteobacter	Alphaproteol	Rhizobiales(1	Phyllobacteri	Mesorhizobium(100)
Otu00547	1	2	7	1	21				0.04092	Bacteria(100	Actinobacter	Actinobacter	Streptomyce	Streptomyce	unclassified(91)
Otu00425	0	4	6	1	21			0.016515		Bacteria(100	Proteobacter	Alphaproteol	Rhizobiales(1	Rhizobiaceae	unclassified(82)
Otu00999	6	2	1	3	21			0.024742		Bacteria(100	Candidate_d	unclassified(1	unclassified(1	unclassified(1	unclassified(100)
Otu00321	5	1	2	5	21			0.035376		Bacteria(100	Proteobacter	Alphaproteol	Rhizobiales(1	Hyphomicrol	Rhodoplanes(100)
Otu00728	5	1	2	5	21			0.035376		Bacteria(100	Gemmatimo	Gemmatimo	Gemmatimo	Gemmatimo	Gemmatimonas(54)
Otu01166	6	4	0	1	21		0.048425			Bacteria(100	Bacteroidete	Flavobacterii	Flavobacteria	Flavobacteria	Flavobacterium(100)
Otu00351	2	6	1	3	21	0.045288				Bacteria(100	Proteobacter	Gammaprote	Pseudomona	Pseudomona	Pseudomonas(100)
Otu00603	0	0	10	0	20	0.003011			0.003194	Bacteria(100	unclassified	unclassified	unclassified	unclassified	unclassified
Otu00270	3	3	0	8	20				0.003697	Bacteria(100	Proteobacter	Alphaproteol	Sphingomona	Sphingomona	Sphingomonas(93)
Otu00616	0	1	9	0	20	0.012965			0.007747	Bacteria(100	Cyanobacter	unclassified(1	unclassified(1	unclassified(1	unclassified(100)
Otu00622	0	6	4	0	20			0.029292	0.01573	Bacteria(100	Actinobacter	Actinobacter	unclassified(9	unclassified(9	unclassified(90)
Otu00546	4	2	0	8	20				0.01573	Bacteria(100	Gemmatimo	Gemmatimo	Gemmatimo	Gemmatimo	unclassified(100)
Otu00495	2	4	4	0	20				0.01573	Bacteria(100	Actinobacter	Actinobacter	Micrococcale	Microbacteri	unclassified(100)
Otu00412	7	0	0	6	20			0.004565	0.027639	Bacteria(100	Proteobacter	Alphaproteol	Sphingomona	Ellin6055(77)	unclassified(77)
Otu00413	4	3	0	6	20				0.027639	Bacteria(100	Bacteroidete	Flavobacterii	Flavobacteria	Flavobacteria	Flavobacterium(100)
Otu02186	4	0	1	10	20				0.031309	Bacteria(100	Acidobacteri	Acidobacteri	Subgroup_6(	unclassified(1	unclassified(100)
Otu00780	0	1	9	0	20	0.043589			0.033411	Bacteria(100	Cyanobacter	unclassified(1	unclassified(1	unclassified(1	unclassified(100)
Otu00303	2	1	2	10	20				0.039787	Bacteria(100	Chloroflexi(1	S085(100)	unclassified(1	unclassified(1	unclassified(100)
Otu01406	0	0	10	0	20	0.03881			0.040179	Bacteria(100	Cyanobacter	unclassified(1	unclassified(1	unclassified(1	unclassified(100)
Otu00621	0	1	9	0	20				0.044726	Bacteria(100	unclassified(7	unclassified(7	unclassified(7	unclassified(7	unclassified(70)
Otu00478	2	4	1	6	20				0.045598	Bacteria(100	Proteobacter	Alphaproteol	Rhizobiales(1	Rhizobiaceae	unclassified(70)
Otu00326	6	0	1	6	20			0		Bacteria(100	Chloroflexi(1	TK10(100)	unclassified(1	unclassified(1	unclassified(100)
Otu00656	4	0	3	6	20			0.016515		Bacteria(100	Chloroflexi(1	JG30-KF-CM6	unclassified(1	unclassified(1	unclassified(100)
Otu02509	7	0	1	4	20			0.044707		Bacteria(100	Planctomyce	unclassified(8	unclassified(8	unclassified(8	unclassified(84)
Otu00734	5	0	3	4	20	0		0.050556		Bacteria(100	Chloroflexi(1	JG30-KF-CM6	unclassified(1	unclassified(1	unclassified(100)
Otu00406	6	2	1	2	20		0.023907			Bacteria(100	Proteobacter	Alphaproteol	Sphingomona	Sphingomona	Sphingobium(100)
Otu01374	5	1	0	7	19				0.004504	Bacteria(100	Bacteroidete	Sphingobact	Sphingobact	AKYH767(10	unclassified(100)
Otu01052	2	1	0	13	19		0.011245		0.005969	Bacteria(100	Chloroflexi(1	Ktedonobact	C0119(100)	unclassified(1	unclassified(100)
Otu01695	4	0	1	9	19				0.015228	Bacteria(100	Chloroflexi(1	Anaerolineae	Anaerolineal	Anaerolineac	unclassified(100)
Otu00494	6	0	1	5	19				0.033997	Bacteria(100	Proteobacter	Alphaproteol	Sphingomona	Sphingomona	Sphingomonas(100)
Otu00986	6	0	1	5	19				0.033997	Bacteria(100	Chloroflexi(1	Thermomicro	AKYG1722(1	unclassified(1	unclassified(100)
Otu00477	0	3	6	1	19			0	0.045598	Bacteria(100	Proteobacter	Alphaproteol	Rhizobiales(1	Rhizobiaceae	unclassified(90)
Otu02896	7	0	0	5	19			0.044707	0.048812	Bacteria(100	Bacteroidete	Sphingobact	Sphingobact	env.OPS_17(	unclassified(100)
Otu00420	4	0	3	5	19			0.016515		Bacteria(100	Proteobacter	Betaproteob	Nitrosomona	Nitrosomona	Nitrosospira(100)
Otu01697	1	1	3	9	19		0.01433			Bacteria(100	unclassified(1	unclassified(1	unclassified(1	unclassified(1	unclassified(100)
Otu00499	2	0	3	9	19	0				Bacteria(100	Chloroflexi(1	Chloroflexia(	Chloroflexale	Roseiflexace	Roseiflexus(100)
Otu00453	3	0	3	7	19	0				Bacteria(100	Acidobacteri	Acidobacteri	Subgroup_4(	Unknown_Fa	Blastocatella(100)
Otu00673	0	0	9	0	18	0			0	Bacteria(100	Cyanobacter	unclassified(1	unclassified(1	unclassified(1	unclassified(100)
Otu00671	0	1	8	0	18	0.009897			0.003697	Bacteria(100	Cyanobacter	unclassified(7	unclassified(7	unclassified(7	unclassified(78)
Otu00579	0	5	4	0	18			0.009517	0.01573	Bacteria(100	Actinobacter	Actinobacter	unclassified(8	unclassified(8	unclassified(89)
Otu01694	4	0	2	6	18			0.016515	0.02391	Bacteria(100	Acidobacteri	Acidobacteri	Subgroup_3(	Unknown_Fa	Bryobacter(100)
Otu00398	4	2	0	6	18				0.027639	Bacteria(100	Proteobacter	Alphaproteol	Sphingomona	Sphingomona	Sphingomonas(92)
Otu00705	0	3	6	0	18				0.027639	Bacteria(100	Cyanobacter	unclassified(1	unclassified(1	unclassified(1	unclassified(100)
Otu00590	2	3	0	8	18				0.032875	Bacteria(100	Acidobacteri	Acidobacteri	Acidobacteri	Acidobacteri	Granulicella(100)
Otu00459	4	1	0	8	18				0.032875	Bacteria(100	Chloroflexi(1	Chloroflexia(	Chloroflexale	Roseiflexace	Roseiflexus(100)
Otu00681	0	2	7	0	18				0.043375	Bacteria(100	Actinobacter	Actinobacter	Micromonos	Micromonos	Actinoplanes(67)
Otu00487	0	1	1	14	18		0.04178		0.045093	Bacteria(100	Acidobacteri	Acidobacteri	Subgroup_2(	unclassified(1	unclassified(100)
Otu01356	5	0	1	6	18				0.045598	Bacteria(100	Bacteroidete	Sphingobact	Sphingobact	Chitinophaga	Terrimonas(100)
Otu00722	2	2	5	0	18				0.048812	Bacteria(100	Proteobacter	Alphaproteol	Rhizobiales(1	Rhizobiaceae	unclassified(56)
Otu01990	0	3	6	0	18			0		Bacteria(100	Actinobacter	Actinobacter	Streptomyce	Streptomyce	Streptomyces(100)
Otu00958	6	2	0	2	18		0.023907	0.024742		Bacteria(100	Cyanobacter	Melainabact	Vampirovibri	unclassified(1	unclassified(100)
Otu01158	6	0	0	6	18			0.029292		Bacteria(100	Gemmatimo	Gemmatimo	Gemmatimo	Gemmatimo	unclassified(100)
Otu00537	8	0	0	2	18			0.033903		Bacteria(100	Proteobacter	Alphaproteol	Sphingomona	Sphingomona	Sphingomonas(100)
Otu00577	5	1	2	2	18			0.035376		Bacteria(100	Actinobacter	Actinobacter	Micrococcale	Intrasporang	Intrasporangium(80)
Otu02861	0	1	6	4	18		0.015426			Bacteria(100	Bacteroidete	Sphingobact	Sphingobact	env.OPS_17(	unclassified(100)
Otu00655	3	0	3	6	18	0				Bacteria(100	Candidate_d	unclassified(1	unclassified(1	unclassified(1	unclassified(100)

Otu00668	3	2	0	7	17				0.004504	Bacteria(100	Bacteroidete	Cytophagia(1	Cytophagales	Cytophagace	unclassified(100)
Otu01692	2	2	1	7	17		0.027362		0.013275	Bacteria(100	Acidobacteri	Acidobacteri	Subgroup_3(	Unknown_Fa	Bryobacter(100)
Otu01526	1	0	7	1	17	0.003819			0.013275	Bacteria(100	Proteobacter	Alphaproteol	Caulobactera	Caulobactera	unclassified(78)
Otu00403	4	1	1	5	17				0.033997	Bacteria(100	Proteobacter	Betaproteob	Burkholderia	Comamonad	unclassified(91)
Otu00584	1	1	6	1	17	0.045288			0.045598	Bacteria(100	Proteobacter	Alphaproteol	Rhizobiales(1	Hyphomicrol	Devosia(56)
Otu00612	4	2	0	5	17				0.048812	Bacteria(100	Gemmatimo	Gemmatimo	Gemmatimo	Gemmatimo	unclassified(100)
Otu01194	5	0	2	3	17			0.009517		Bacteria(100	Proteobacter	Gammaprote	unclassified(8	unclassified(8	unclassified(80)
Otu00715	4	0	3	3	17			0.016515		Bacteria(100	Proteobacter	Gammaprote	Xanthomona	Xanthomona	Rhodanobacter(100)
Otu00604	4	0	3	3	17			0.016515		Bacteria(100	Proteobacter	Alphaproteol	Rhizobiales(1	Bradyrhizobi	Bradyrhizobium(90)
Otu01961	6	1	0	3	17			0.048221		Bacteria(100	Chloroflexi(1	KD4-96(100)	unclassified(	unclassified(	unclassified(100)
Otu01305	5	0	2	3	17			0.050556		Bacteria(100	Gemmatimo	Gemmatimo	S0134_terres	unclassified(	unclassified(100)
Otu00924	0	1	2	11	17		0.035829			Bacteria(100	Proteobacter	Deltaproteob	Myxococcale	unclassified(	unclassified(100)
Otu00727	0	1	6	3	17	0.00557				Bacteria(100	Acidobacteri	Acidobacteri	Subgroup_4(	RB41(100)	unclassified(100)
Otu00694	4	4	0	1	17	0.013185				Bacteria(100	Proteobacter	Alphaproteol	Rhizobiales(1	Hyphomicrol	unclassified(78)
Otu00744	3	0	4	3	17	0.013185				Bacteria(100	Chloroflexi(1	Ktedonobact	C0119(100)	unclassified(	unclassified(100)
Otu00798	0	3	5	0	16				0.009016	Bacteria(100	unclassified(	unclassified(	unclassified(	unclassified(	unclassified(100)
Otu00684	0	3	5	0	16				0.009016	Bacteria(100	unclassified(	unclassified(	unclassified(	unclassified(	unclassified(100)
Otu01541	0	0	8	0	16	0.013185			0.01573	Bacteria(100	Cyanobacteri	unclassified(7	unclassified(7	unclassified(7	unclassified(75)
Otu00466	3	3	0	4	16				0.01573	Bacteria(100	Proteobacter	Alphaproteol	Sphingomon	Sphingomon	Sphingomonas(100)
Otu00529	4	2	0	4	16				0.01573	Bacteria(100	Proteobacter	Alphaproteol	Rhizobiales(1	Hyphomicrol	Pedomicrobium(100)
Otu00903	3	0	1	8	16				0.031652	Bacteria(100	Bacteroidete	Sphingobact	Sphingobact	Saprospirace	unclassified(100)
Otu01111	4	0	0	8	16				0.032875	Bacteria(100	Gemmatimo	Gemmatimo	Gemmatimo	Gemmatimo	Gemmatimonas(92)
Otu00923	3	1	1	6	16				0.045598	Bacteria(100	Acidobacteri	Acidobacteri	Acidobacteri	Acidobacteri	Acidobacterium(100)
Otu01391	3	1	1	6	16				0.045598	Bacteria(100	Acidobacteri	Acidobacteri	Acidobacteri	Acidobacteri	unclassified(100)
Otu00723	3	0	3	4	16			0		Bacteria(100	Acidobacteri	Acidobacteri	Subgroup_6(	unclassified(	unclassified(100)
Otu00689	7	0	0	2	16			0.004565		Bacteria(100	Acidobacteri	Acidobacteri	Subgroup_6(	unclassified(	unclassified(100)
Otu00550	6	1	0	2	16			0.008355		Bacteria(100	Acidobacteri	Acidobacteri	Subgroup_4(	Unknown_Fa	Blastocatella(100)
Otu00557	6	1	0	2	16			0.008355		Bacteria(100	Proteobacter	Alphaproteol	Rhizobiales(1	Rhizobiaceae	unclassified(78)
Otu00541	4	0	3	2	16			0.016515		Bacteria(100	Acidobacteri	Acidobacteri	Subgroup_4(	Unknown_Fa	Blastocatella(100)
Otu01424	4	0	3	2	16			0.016515		Bacteria(100	Proteobacter	Gammaprote	Xanthomona	Xanthomona	Dokdonella(100)
Otu00945	4	0	1	6	16			0.016515		Bacteria(100	Proteobacter	Betaproteob	Methylophila	Methylophila	unclassified(100)
Otu00982	4	0	2	4	16			0.016515		Bacteria(100	Chloroflexi(1	Ktedonobact	C0119(100)	unclassified(	unclassified(100)
Otu01124	6	0	2	0	16		0.030672	0.029292		Bacteria(100	Cyanobacteri	ML635J-21(1	unclassified(	unclassified(	unclassified(100)
Otu00601	5	1	0	4	16			0.035376		Bacteria(100	Proteobacter	Alphaproteol	Sphingomon	Erythrobacte	Altererythrobacter(100)
Otu01456	5	0	0	6	16			0.050556		Bacteria(100	Candidate_d	unclassified(	unclassified(	unclassified(	unclassified(100)
Otu00884	0	1	4	6	16		0.030672			Bacteria(100	Proteobacter	Betaproteob	Methylophila	Methylophila	Methylobacillus(100)
Otu00573	4	3	0	2	16	0				Bacteria(100	Proteobacter	Gammaprote	Pseudomona	Pseudomona	Pseudomonas(100)
Otu00770	4	3	0	2	16	0				Bacteria(100	Bacteroidete	Sphingobact	Sphingobact	Chitinophaga	Flavisolibacter(100)
Otu00854	3	4	0	2	16	0.013185				Bacteria(100	Proteobacter	Gammaprote	Pseudomona	Pseudomona	Pseudomonas(100)
Otu01966	2	6	0	0	16	0.026001				Bacteria(100	Proteobacter	Gammaprote	Xanthomona	Xanthomona	Pseudoxanthomonas(100)
Otu01938	4	2	0	3	15				0	Bacteria(100	Proteobacter	Alphaproteol	Rhodospirilla	Rhodospirilla	unclassified(100)
Otu01013	3	3	0	3	15				0	Bacteria(100	Acidobacteri	Holophagae(	Subgroup_1	ABS-19(100)	unclassified(100)
Otu00567	2	2	0	7	15		0.027362		0.004504	Bacteria(100	Proteobacter	Betaproteob	Burkholderia	Comamonad	Ramlibacter(100)
Otu00436	4	0	0	7	15				0.004504	Bacteria(100	Proteobacter	Alphaproteol	Sphingomon	Sphingomon	Sphingomonas(82)
Otu01626	5	0	0	5	15			0.009517	0.009016	Bacteria(100	Chloroflexi(1	TK10(100)	unclassified(	unclassified(	unclassified(100)
Otu01426	3	2	0	5	15				0.009016	Bacteria(100	Bacteroidete	Sphingobact	Sphingobact	Chitinophaga	Flavisolibacter(100)
Otu02885	2	1	1	7	15				0.04092	Bacteria(100	Chloroflexi(1	S085(100)	unclassified(	unclassified(	unclassified(100)
Otu00927	4	0	0	7	15				0.043375	Bacteria(100	Gemmatimo	Gemmatimo	Gemmatimo	Gemmatimo	Gemmatimonas(100)
Otu00868	3	2	0	5	15				0.048812	Bacteria(100	Proteobacter	Alphaproteol	Sphingomon	Sphingomon	Sphingomonas(80)
Otu01170	4	1	0	5	15				0.048812	Bacteria(100	Chloroflexi(1	TK10(100)	unclassified(	unclassified(	unclassified(100)
Otu00704	3	0	1	7	15			0		Bacteria(100	Proteobacter	Betaproteob	SC-I-84(100)	unclassified(	unclassified(100)
Otu00703	5	0	1	3	15			0.009517		Bacteria(100	Proteobacter	Gammaprote	Xanthomona	Xanthomona	Rhodanobacter(78)
Otu00623	4	0	2	3	15			0.016515		Bacteria(100	Chloroflexi(1	Thermomicr	JG30-KF-CM	unclassified(	unclassified(100)
Otu00812	6	0	0	3	15			0.029292		Bacteria(100	Actinobacter	unclassified(	unclassified(	unclassified(	unclassified(100)
Otu00807	5	1	0	3	15			0.035376		Bacteria(100	Candidate_d	unclassified(	unclassified(	unclassified(	unclassified(100)
Otu00997	5	0	0	5	15			0.050556		Bacteria(100	Proteobacter	Gammaprote	unclassified(	unclassified(	unclassified(100)
Otu00626	0	1	3	7	15		0.003247			Bacteria(100	Proteobacter	Betaproteob	Burkholderia	Comamonad	unclassified(100)
Otu00628	2	0	3	5	15	0				Bacteria(100	Chloroflexi(1	S085(100)	unclassified(	unclassified(	unclassified(100)
Otu00691	3	0	3	3	15	0				Bacteria(100	Proteobacter	Betaproteob	Burkholderia	Comamonad	unclassified(67)
Otu00687	2	5	0	1	15	0.006405				Bacteria(100	Proteobacter	Alphaproteol	Rhizobiales(1	Rhizobiaceae	unclassified(88)
Otu00686	3	4	0	1	15	0.013185				Bacteria(100	Proteobacter	Alphaproteol	Rhizobiales(1	unclassified(6	unclassified(63)
Otu01531	3	4	0	1	15	0.013185				Bacteria(100	Proteobacter	Betaproteob	Burkholderia	Comamonad	Acidovorax(75)
Otu00847	3	1	3	0	14				0	Bacteria(100	Candidate_d	unclassified(	unclassified(	unclassified(	unclassified(100)
Otu01129	1	3	3	0	14				0	Bacteria(100	Proteobacter	Alphaproteol	Rhizobiales(1	Hyphomicrol	unclassified(72)
Otu00824	0	0	7	0	14	0.003819			0.004504	Bacteria(100	unclassified(	unclassified(	unclassified(	unclassified(	unclassified(100)
Otu00663	3	0	0	8	14			0	0.01573	Bacteria(100	Acidobacteri	Acidobacteri	Acidobacteri	Acidobacteri	unclassified(100)
Otu01221	5	0	0	4	14			0.009517	0.01573	Bacteria(100	Acidobacteri	Acidobacteri	Subgroup_3(	Unknown_Fa	Bryobacter(100)
Otu00886	3	0	4	0	14	0.013185			0.01573	Bacteria(100	Proteobacter	Alphaproteol	Sphingomon	Sphingomon	Sphingobium(86)
Otu01248	4	1	0	4	14				0.01573	Bacteria(100	Chloroflexi(8	unclassified(7	unclassified(7	unclassified(7	unclassified(78)
Otu00822	0	3	4	0	14				0.01573	Bacteria(100	unclassified(	unclassified(	unclassified(	unclassified(	unclassified(100)
Otu01090	0	3	4	0	14				0.01573	Bacteria(100	Actinobacter	Actinobacter	Micrococcale	Microbacteri	unclassified(58)
Otu00904	1	2	4	0	14				0.01573	Bacteria(100	Candidate_d	unclassified(	unclassified(	unclassified(	unclassified(100)
Otu00783	0	0	7	0	14	0.042082			0.043375	Bacteria(100	Cyanobacteri	unclassified(5	unclassified(5	unclassified(5	unclassified(58)
Otu01163	0	0	7	0	14	0.042082			0.043375	Bacteria(100	Cyanobacteri	unclassified(	unclassified(	unclassified(	unclassified(100)
Otu00564	2	1	1	6	14				0.045598	Bacteria(100	Proteobacter	Betaproteob	Burkholderia	Oxalobactera	Massilia(80)
Otu00713	2	1	1	6	14				0.045598	Bacteria(100	Proteobacter	Deltaproteob	GR-WP33-30	unclassified(	unclassified(100)
Otu01191	2	0	0	10	14				0.048812	Bacteria(100	Proteobacter	Alphaproteol	Rhizobiales(1	KF-JG30-B3(1	unclassified(100)
Otu01488	3	3	0	2	14	0		0		Bacteria(100	Chloroflexi(1	S085(100)	unclassified(	unclassified(	unclassified(100)
Otu02319	0	3	4	0	14			0		Bacteria(100	Cyanobacteri	unclassified(	unclassified(	unclassified(	unclassified(100)
Otu00844	5	0	1	2	14			0.009517		Bacteria(100	Proteobacter	Alphaproteol	Sphingomon	Sphingomon	Sphingomonas(100)
Otu00708	5	0	0	4	14			0.009517		Bacteria(100	Bacteroidete	Sphingobact	Sphingobact	Chitinophaga	Terrimonas(100)
Otu00779	5	0	0	4	14			0.009517		Bacteria(100	Chloroflexi(1	Chloroflexia(	Chloroflexale	Roseiflexacea	Roseiflexus(100)
Otu01321	4	0	1	4	14			0.016515		Bacteria(100	Acidobacteri	Acidobacteri	Subgroup_3(	Unknown_Fa	Candidatus_Solibacter(100)
Otu01068	0	4	3	0	14			0.016515		Bacteria(100	Gemmatimo	Gemmatimo	Gemmatimo	Gemmatimo	unclassified(72)
Otu00902	0	6	1	0	14	0.045288		0.029292		Bacteria(100	Actinobacter	Actinobacter	Streptomyce	Streptomyce	Streptomyces(58)
Otu01062	1	5	1	0	14	0.032618		0.035376		Bacteria(100	Proteobacter	Gammaprote	Xanthomona	Xanthomona	Steroidobacter(100)
Otu00900	5	0	0	4	14			0.050556		Bacteria(100	Gemmatimo	Gemmatimo	Gemmatimo	Gemmatimo	unclassified(100)
Otu01355	3	4	0	0	14		0			Bacteria(100	Proteobacter	Alphaproteol	Sphingomon	unclassified(	unclassified(100)
Otu00795	4	1	2	0	14		0.015426			Bacteria(100	Proteobacter	Alphaproteol	Rhodospirilla	Rhodospirilla	Dongia(100)
Otu00889	4	1	2	0	14		0.015426			Bacteria(100	Proteobacter	Alphaproteol	Sphingomon	unclassified(8	unclassified(86)
Otu02379	2	0	3	4	14	0				Bacteria(100	Acidobacteri	Acidobacteri	Subgroup_4(	RB41(100)	unclassified(100)
Otu00747	1	5	0	2	14	0.048612				Bacteria(100	Proteobacter	Gammaprote	Pseudomona	Pseudomona	Pseudomonas(100)
Otu00627	2	1	0	7	13		0.027362		0.004504	Bacteria(100	Gemmatimo	Gemmatimo	Gemmatimo	Gemmatimo	unclassified(100)
Otu00634	3	0	0	7	13				0.004504	Bacteria(100	Verrucomicr	Spartobacter	Chthoniobac	Xiphinemato	Candidatus_Xiphinematobacter(100)
Otu01105	3	0	0	7	13				0.004504	Bacteria(100	Acidobacteri	Acidobacteri	Subgroup_3(	Unknown_Fa	Candidatus_Solibacter(100)
Otu02742	2	2	0	5	13				0.009016	Bacteria(100	Candidate_d	unclassified(	unclassified(	unclassified(	unclassified(100)
Otu00771	1	1	1	7	13		0.011794		0.013275	Bacteria(100	Gemmatimo	Gemmatimo	Gemmatimo	Gemmatimo	unclassified(100)
Otu00649	0	3	1	5	13		0.006324		0.033997	Bacteria(100	Proteobacter	Betaproteob	Nitrosomona	Nitrosomona	unclassified(100)
Otu00852	3	0	1	5	13				0.033997	Bacteria(100	Gemmatimo	Gemmatimo	Gemmatimo	Gemmatimo	Gemmatimonas(100)
Otu00947	3	0	1	5	13				0.033997	Bacteria(100	Proteobacter	Alphaproteol	Rhizobiales(1	Xanthobacte	Pseudolabrys(56)
Otu00534	2	0	1	7	13				0.04092	Bacteria(100	Gemmatimo	Gemmatimo	Gemmatimo	Gemmatimo	unclassified(100)
Otu00930	2	0	1	7	13				0.04092	Bacteria(100	Gemmatimo	Gemmatimo	Gemmatimo	Gemmatimo	unclassified(100)

Otu00791	3	3	0	1	13	0		0		Bacteria(100	Proteobacter	Alphaproteol	Sphingomon	unclassified(	unclassified(72)
Otu01275	0	3	3	1	13	0		0		Bacteria(100	Actinobacter	Actinobacter	Propionibact	Nocardioidac	Aeromicrobium(86)
Otu00888	3	0	3	1	13			0		Bacteria(100	Proteobacter	Betaproteob	Burkholderia	Comamonad	Paucibacter(58)
Otu01952	3	0	3	1	13			0		Bacteria(100	Chloroflexi(1	Chloroflexia(	Herpetosipho	Herpetosipho	Herpetosiphon(100)
Otu01283	5	0	1	1	13		0.03709	0.009517		Bacteria(100	Chloroflexi(1	S085(100)	unclassified(	unclassified(	unclassified(100)
Otu01332	4	0	1	3	13			0.016515		Bacteria(100	Acidobacteri	Acidobacteri	Subgroup_6(	unclassified(	unclassified(100)
Otu01827	4	0	1	3	13			0.016515		Bacteria(100	Bacteroidete	Sphingobact	Sphingobact	unclassified(	unclassified(100)
Otu00680	4	0	0	5	13			0.016515		Bacteria(100	Proteobacter	Alphaproteol	Sphingomon	Sphingomon	Sphingomonas(89)
Otu00919	6	0	0	1	13		0.048425	0.029292		Bacteria(100	Proteobacter	Betaproteob	Burkholderia	Oxalobactera	Massilia(86)
Otu01465	5	0	1	1	13			0.050556		Bacteria(100	Bacteroidete	Flavobacterii	Flavobacteria	Flavobacteria	Flavobacterium(100)
Otu00915	2	4	0	1	13	0.013185				Bacteria(100	Bacteroidete	Sphingobact	Sphingobact	Chitinophaga	Flavisolibacter(100)
Otu01028	0	0	5	3	13	0.048612				Bacteria(100	Chloroflexi(1	Anaerolineae	Anaerolineal	Anaerolineac	unclassified(100)
Otu01225	0	3	3	0	12				0	Bacteria(100	unclassified(	unclassified(	unclassified(	unclassified(	unclassified(84)
Otu00485	1	1	0	8	12		0.010589		0.003697	Bacteria(100	Proteobacter	Alphaproteol	Sphingomon	Sphingomon	Sphingomonas(100)
Otu00637	0	0	1	10	12		0.002656		0.006271	Bacteria(100	Chloroflexi(1	JG30-KF-CM	unclassified(	unclassified(	unclassified(100)
Otu00751	1	1	1	6	12		0.005249		0.00797	Bacteria(100	Chloroflexi(1	Chloroflexia(	Chloroflexale	Roseiflexacea	Roseiflexus(100)
Otu01132	0	1	5	0	12	0.032618			0.009016	Bacteria(100	Cyanobacteri	unclassified(	unclassified(	unclassified(	unclassified(100)
Otu01273	0	1	5	0	12	0.032618			0.009016	Bacteria(100	Cyanobacteri	unclassified(	unclassified(	unclassified(	unclassified(100)
Otu01499	0	1	5	0	12	0.032618			0.009016	Bacteria(100	Cyanobacteri	unclassified(	unclassified(	unclassified(	unclassified(67)
Otu01231	3	1	0	4	12				0.01573	Bacteria(100	Proteobacter	Alphaproteol	Rhodospirilla	DA111(88)	unclassified(88)
Otu01048	4	0	0	4	12				0.01573	Bacteria(100	Acidobacteri	Acidobacteri	Subgroup_6(	unclassified(	unclassified(100)
Otu01002	0	2	4	0	12				0.01573	Bacteria(100	Proteobacter	Gammaprote	unclassified(	unclassified(	unclassified(67)
Otu01095	0	2	4	0	12				0.01573	Bacteria(100	unclassified(	unclassified(	unclassified(	unclassified(	unclassified(100)
Otu01224	1	1	4	0	12				0.01573	Bacteria(100	Proteobacter	Betaproteob	Burkholderia	Oxalobactera	unclassified(84)
Otu01590	0	0	6	0	12	0.026001			0.027639	Bacteria(100	Chloroflexi(1	Chloroflexia(	Chloroflexale	Roseiflexacea	Roseiflexus(100)
Otu01937	0	0	6	0	12	0.026001			0.027639	Bacteria(100	Cyanobacteri	unclassified(	unclassified(	unclassified(	unclassified(100)
Otu00636	3	0	0	6	12				0.027639	Bacteria(100	Proteobacter	Alphaproteol	unclassified(	unclassified(	unclassified(56)
Otu00921	3	0	0	6	12				0.027639	Bacteria(100	Chloroflexi(1	Chloroflexia(	Herpetosipho	Herpetosipho	Herpetosiphon(100)
Otu01161	0	1	5	0	12				0.048812	Bacteria(100	unclassified(	unclassified(	unclassified(	unclassified(	unclassified(84)
Otu01684	0	1	5	0	12				0.048812	Bacteria(100	Gemmatimo	Gemmatimo	Gemmatimo	Gemmatimo	Gemmatimonas(100)
Otu00878	3	0	2	2	12			0		Bacteria(100	Proteobacter	Alphaproteol	Sphingomon	Sphingomon	Sphingomonas(86)
Otu01039	0	3	2	2	12			0		Bacteria(100	Actinobacter	Actinobacter	Micrococcale	Microbacteri	unclassified(58)
Otu00981	3	0	1	4	12			0		Bacteria(100	Bacteroidete	Sphingobact	Sphingobact	Chitinophaga	unclassified(100)
Otu01388	3	0	1	4	12			0		Bacteria(100	Acidobacteri	Holophagae(	Subgroup_7(	unclassified(	unclassified(100)
Otu01071	0	3	3	0	12			0		Bacteria(100	Actinobacter	Actinobacter	Micrococcale	unclassified	unclassified
Otu01014	4	0	2	0	12		0.015426	0.016515		Bacteria(100	Actinobacter	Actinobacter	Micrococcale	Micrococcac	unclassified
Otu00777	4	0	1	2	12			0.016515		Bacteria(100	Proteobacter	Alphaproteol	Sphingomon	Sphingomon	Sphingomonas(58)
Otu00768	4	0	0	4	12			0.016515		Bacteria(100	Proteobacter	Alphaproteol	Rhizobiales(1	Hyphomicrob	Pedomicrobium(100)
Otu01433	4	0	0	4	12			0.016515		Bacteria(100	Nitrospirae(1	Nitrospira(10	Nitrospirales	Nitrospiracea	Nitrospira(100)
Otu01470	0	4	2	0	12			0.016515		Bacteria(100	Actinobacter	Actinobacter	Streptomyce	Streptomyce	Streptomyces(100)
Otu01246	5	0	1	0	12			0.050556		Bacteria(100	Actinobacter	Actinobacter	Corynebacte	Mycobacteri	Mycobacterium(100)
Otu01497	5	0	1	0	12			0.050556		Bacteria(100	Chloroflexi(1	Caldilineae(1	Caldilineales	Caldilineacea	unclassified(100)
Otu00937	5	0	0	2	12			0.050556		Bacteria(100	Proteobacter	Alphaproteol	Rhizobiales(1	unclassified(	unclassified(72)
Otu01934	0	5	1	0	12			0.050556		Bacteria(100	Actinobacter	Actinobacter	unclassified(	unclassified(	unclassified(100)
Otu01055	4	1	1	0	12		0.015426			Bacteria(100	Gemmatimo	Gemmatimo	S0134_terres	unclassified(	unclassified(100)
Otu01010	4	2	0	0	12		0.015426			Bacteria(100	Proteobacter	Alphaproteol	Sphingomon	Sphingomon	Sphingomonas(84)
Otu01530	3	3	0	0	12	0				Bacteria(100	Chloroflexi(1	Thermomicro	JG30-KF-CM	unclassified(	unclassified(100)
Otu01708	2	0	3	2	12	0				Bacteria(100	unclassified(	unclassified(	unclassified(	unclassified(	unclassified(100)
Otu01143	1	0	4	2	12	0.013185				Bacteria(100	Armatimona	unclassified(	unclassified(	unclassified(	unclassified(58)
Otu00855	4	0	0	3	11			0.016515	0	Bacteria(100	Acidobacteri	Acidobacteri	Acidobacteri	Acidobacteri	unclassified(100)
Otu02274	2	0	0	7	11		0.027362		0.004504	Bacteria(100	Bacteroidete	Sphingobact	Sphingobact	env.OPS_17(	unclassified(100)
Otu01410	2	1	0	5	11				0.009016	Bacteria(100	Gemmatimo	Gemmatimo	Gemmatimo	Gemmatimo	unclassified(100)
Otu00789	3	0	0	5	11				0.009016	Bacteria(100	Gemmatimo	Gemmatimo	Gemmatimo	Gemmatimo	unclassified(100)
Otu01067	0	2	1	5	11		0.006324		0.033997	Bacteria(100	Proteobacter	Alphaproteol	Rhodospirilla	DA111(100)	unclassified(100)
Otu00992	0	0	5	1	11	0.006405			0.033997	Bacteria(100	Proteobacter	Deltaproteob	Bdellovibrior	Bdellovibrior	Bdellovibrio(100)
Otu01083	2	0	1	5	11				0.033997	Bacteria(100	Proteobacter	Betaproteob	Burkholderia	Comamonad	unclassified(63)
Otu01188	0	0	1	9	11		0.036244		0.045287	Bacteria(100	Proteobacter	Alphaproteol	Rhizobiales(1	KF-JG30-B3(1	unclassified(100)
Otu01007	1	2	0	5	11				0.048812	Bacteria(100	Bacteroidete	Sphingobact	Sphingobact	Chitinophaga	Terrimonas(100)
Otu01734	3	0	0	5	11				0.048812	Bacteria(100	BD1-5(88)	unclassified(	unclassified(	unclassified(	unclassified(88)
Otu01421	3	0	1	3	11			0		Bacteria(100	Proteobacter	Alphaproteol	Rhizobiales(1	Rhodobiacea	Rhodobium(100)
Otu00984	4	0	1	1	11			0.016515		Bacteria(100	Proteobacter	Alphaproteol	Sphingomon	Sphingomon	Sphingomonas(100)
Otu01063	4	0	1	1	11			0.016515		Bacteria(100	Proteobacter	Alphaproteol	Sphingomon	unclassified	unclassified
Otu01198	4	0	1	1	11			0.016515		Bacteria(100	Chloroflexi(1	Chloroflexia(	Chloroflexale	Roseiflexacea	Roseiflexus(100)
Otu02311	4	0	0	3	11			0.016515		Bacteria(100	Acidobacteri	Acidobacteri	Subgroup_3(	PAUC26f(10	unclassified(100)
Otu00976	0	4	1	1	11			0.016515		Bacteria(100	Proteobacter	Betaproteob	Burkholderia	Comamonad	Rhizobacter(100)
Otu01089	0	5	0	1	11	0.048612		0.050556		Bacteria(100	Actinobacter	Actinobacter	unclassified	unclassified	unclassified
Otu00898	0	0	4	3	11		0			Bacteria(100	Proteobacter	Betaproteob	Burkholderia	Comamonad	unclassified(58)
Otu01130	1	3	0	3	11	0				Bacteria(100	Acidobacteri	Acidobacteri	Subgroup_3(	Unknown_Fa	Bryobacter(100)
Otu01813	0	2	3	0	10				0	Bacteria(100	unclassified(	unclassified(	unclassified(	unclassified(	unclassified(80)
Otu01542	0	2	3	0	10				0	Bacteria(100	Cyanobacteri	unclassified(	unclassified(	unclassified(	unclassified(100)
Otu01393	0	0	5	0	10	0.006405			0.009016	Bacteria(100	Acidobacteri	Acidobacteri	Subgroup_6(	unclassified(	unclassified(100)
Otu01444	0	0	5	0	10	0.006405			0.009016	Bacteria(100	Chloroflexi(1	Ktedonobact	JG30-KF-AS9	unclassified(	unclassified(100)
Otu01453	0	0	5	0	10	0.006405			0.009016	Bacteria(100	Cyanobacteri	unclassified(	unclassified(	unclassified(	unclassified(100)
Otu01514	0	0	5	0	10	0.006405			0.009016	Bacteria(100	Cyanobacteri	unclassified(	unclassified(	unclassified(	unclassified(61)
Otu01625	0	0	5	0	10	0.006405			0.009016	Bacteria(100	Proteobacter	Alphaproteol	Rickettsiales(	mitochondri	unclassified(100)
Otu01933	0	0	5	0	10	0.006405			0.009016	Bacteria(100	unclassified(	unclassified(	unclassified(	unclassified(	unclassified(80)
Otu02629	1	0	4	0	10	0.013185			0.01573	Bacteria(100	Proteobacter	Gammaprote	Xanthomona	Xanthomona	Steroidobacter(100)
Otu01057	2	1	0	4	10				0.01573	Bacteria(100	Proteobacter	Alphaproteol	Sphingomon	unclassified(	unclassified(72)
Otu01925	2	1	0	4	10				0.01573	Bacteria(100	Candidate_d	unclassified(	unclassified(	unclassified(	unclassified(100)
Otu01243	3	0	0	4	10				0.01573	Bacteria(100	Chloroflexi(1	Anaerolineae	Anaerolineal	Anaerolineac	unclassified(100)
Otu01199	0	1	4	0	10				0.01573	Bacteria(100	unclassified(	unclassified(	unclassified(	unclassified(	unclassified(100)
Otu01336	0	1	4	0	10				0.01573	Bacteria(100	unclassified(	unclassified(	unclassified(	unclassified(	unclassified(80)
Otu01353	0	1	4	0	10				0.01573	Bacteria(100	unclassified(	unclassified(	unclassified(	unclassified(	unclassified(61)
Otu01540	0	1	4	0	10				0.01573	Bacteria(100	unclassified(	unclassified(	unclassified(	unclassified(	unclassified(80)
Otu02408	0	1	4	0	10				0.01573	Bacteria(100	Cyanobacteri	unclassified(	unclassified(	unclassified(	unclassified(61)
Otu01141	1	1	0	6	10		0.048425		0.027639	Bacteria(100	Chloroflexi(1	Chloroflexia(	Chloroflexale	Roseiflexacea	Roseiflexus(100)
Otu00913	2	0	0	6	10				0.027639	Bacteria(100	Gemmatimo	Gemmatimo	Gemmatimo	Gemmatimo	unclassified(100)
Otu01006	2	0	0	6	10				0.027639	Bacteria(100	Proteobacter	Alphaproteol	Rhizobiales(1	Bradyrhizobi	Rhodopseudomonas(75)
Otu01898	1	0	1	6	10		0.048425		0.045598	Bacteria(100	Acidobacteri	Acidobacteri	Subgroup_6(	unclassified(	unclassified(100)
Otu01477	0	0	5	0	10	0.048612			0.048812	Bacteria(100	Acidobacteri	Acidobacteri	Subgroup_6(	unclassified(	unclassified(100)
Otu01918	0	0	5	0	10	0.048612			0.048812	Bacteria(100	unclassified(	unclassified(	unclassified(	unclassified(	unclassified(100)
Otu02121	0	0	5	0	10	0.048612			0.048812	Bacteria(100	Cyanobacteri	unclassified(	unclassified(	unclassified(	unclassified(100)
Otu01372	0	5	0	0	10	0.006405		0.009517		Bacteria(100	Bacteroidete	Sphingobact	Sphingobact	Chitinophaga	Niastella(100)
Otu01490	4	0	1	0	10		0.015426	0.016515		Bacteria(100	Proteobacter	Alphaproteol	Sphingomon	Sphingomon	Sphingomonas(100)
Otu01509	4	0	1	0	10		0.015426	0.016515		Bacteria(100	SHA-109(100	unclassified(	unclassified(	unclassified(	unclassified(100)
Otu01182	0	4	1	0	10			0.016515		Bacteria(100	Actinobacter	Actinobacter	Propionibact	Nocardioidac	Marmoricola(100)
Otu03796	1	0	3	2	10	0				Bacteria(100	Planctomyce	Planctomyce	Planctomyce	Planctomyce	unclassified(100)
Otu01413	1	4	0	0	10	0.013185				Bacteria(100	Candidate_d	unclassified(	unclassified(	unclassified(	unclassified(100)
Otu01116	2	1	0	3	9				0	Bacteria(100	Proteobacter	Deltaproteob	Bdellovibrior	Bacteriovora	Peredibacter(100)
Otu01208	2	1	0	3	9				0	Bacteria(100	Proteobacter	Betaproteob	SC-I-84(100)	unclassified(	unclassified(100)
Otu00985	3	0	0	3	9				0	Bacteria(100	Proteobacter	Betaproteob	Nitrosomona	Nitrosomona	unclassified(100)
Otu01036	3	0	0	3	9				0	Bacteria(100	Armatimona	unclassified(	unclassified(	unclassified(	unclassified(100)

Otu01046	3	0	0	3	9				0	Bacteria(100	Gemmatimo	Gemmatimo	Gemmatimo	Gemmatimo	unclassified(100)
Otu01289	1	0	0	7	9		0.011794		0.004504	Bacteria(100	Acidobacteri	Acidobacteri	Subgroup_6(	unclassified(	unclassified(100)
Otu01495	0	2	0	5	9		0.006324		0.009016	Bacteria(100	Chloroflexi(1	Anaerolineae	Anaerolineal	Anaerolineac	unclassified(100)
Otu01115	2	0	0	5	9				0.009016	Bacteria(100	Proteobacter	Alphaproteol	Sphingomon	Erythrobacte	unclassified(58)
Otu01310	2	0	0	5	9				0.009016	Bacteria(100	Gemmatimo	Gemmatimo	Gemmatimo	Gemmatimo	unclassified(100)
Otu01425	3	0	1	1	9			0		Bacteria(100	Proteobacter	Alphaproteol	Sphingomon	Sphingomon	Sphingomonas(100)
Otu01472	3	0	1	1	9			0		Bacteria(100	Proteobacter	Alphaproteol	unclassified(	unclassified(	unclassified(61)
Otu02628	3	0	1	1	9			0		Bacteria(100	Proteobacter	Betaproteob	unclassified(	unclassified(	unclassified(100)
Otu01432	3	0	0	3	9			0		Bacteria(100	Proteobacter	Alphaproteol	Sphingomon	unclassified(	unclassified(100)
Otu01445	0	3	1	1	9			0		Bacteria(100	Proteobacter	Betaproteob	SC-I-84(100)	unclassified(	unclassified(100)
Otu01102	0	2	1	3	9		0			Bacteria(100	Proteobacter	Alphaproteol	Rhizobiales(1	Hyphomicrol	unclassified(84)
Otu01912	0	1	2	3	9		0			Bacteria(100	Proteobacter	Deltaproteol	Bdellovibrior	Bdellovibrior	Bdellovibrio(100)
Otu01915	1	0	3	0	8	0			0	Bacteria(100	Proteobacter	Alphaproteol	Rhizobiales(1	Xanthobacte	Pseudolabrys(75)
Otu01719	0	1	3	0	8				0	Bacteria(100	Cyanobacteri	unclassified(	unclassified(	unclassified(	unclassified(100)
Otu01892	0	1	3	0	8				0	Bacteria(100	Cyanobacteri	unclassified(	unclassified(	unclassified(	unclassified(100)
Otu02549	0	1	3	0	8				0	Bacteria(100	Cyanobacteri	unclassified(	unclassified(	unclassified(	unclassified(100)
Otu01093	0	2	0	4	8		0.015426		0.01573	Bacteria(100	Gemmatimo	Gemmatimo	Gemmatimo	Gemmatimo	Gemmatimonas(100)
Otu01599	0	0	4	0	8	0.013185			0.01573	Bacteria(100	Cyanobacteri	unclassified(	unclassified(	unclassified(	unclassified(75)
Otu01698	0	0	4	0	8	0.013185			0.01573	Bacteria(100	Gemmatimo	Gemmatimo	Gemmatimo	Gemmatimo	unclassified(100)
Otu01793	0	0	4	0	8	0.013185			0.01573	Bacteria(100	Cyanobacteri	unclassified(	unclassified(	unclassified(	unclassified(75)
Otu01832	0	0	4	0	8	0.013185			0.01573	Bacteria(100	unclassified	unclassified	unclassified	unclassified	unclassified
Otu01976	0	0	4	0	8	0.013185			0.01573	Bacteria(100	Actinobacter	Actinobacter	Micromonos	Micromonos	unclassified(100)
Otu02233	0	0	4	0	8	0.013185			0.01573	Bacteria(100	unclassified	unclassified	unclassified	unclassified	unclassified
Otu02897	0	0	4	0	8	0.013185			0.01573	Bacteria(100	Cyanobacteri	unclassified(	unclassified(	unclassified(	unclassified(100)
Otu00979	1	1	0	4	8				0.01573	Bacteria(100	Proteobacter	Alphaproteol	Sphingomon	Erythrobacte	unclassified(84)
Otu01219	1	1	0	4	8				0.01573	Bacteria(100	Gemmatimo	Gemmatimo	Gemmatimo	Gemmatimo	Gemmatimonas(100)
Otu01972	1	1	0	4	8				0.01573	Bacteria(100	Gemmatimo	Gemmatimo	Gemmatimo	Gemmatimo	unclassified(67)
Otu01140	2	0	0	4	8				0.01573	Bacteria(100	Acidobacteri	Acidobacteri	Acidobacteri	Acidobacteri	unclassified(67)
Otu01511	2	0	0	4	8				0.01573	Bacteria(100	Verrucomicr	Opitutae(10	Opitutales(1	Opitutaceae(	Opitutus(100)
Otu02644	3	0	1	0	8		0	0		Bacteria(100	Acidobacteri	Acidobacteri	Subgroup_6(	unclassified(	unclassified(100)
Otu01607	3	0	0	2	8			0		Bacteria(100	Acidobacteri	Acidobacteri	Subgroup_3(	Unknown_Fa	Bryobacter(100)
Otu01846	0	3	1	0	8			0		Bacteria(100	Actinobacter	Actinobacter	Micromonos	Micromonos	unclassified(75)
Otu01776	4	0	0	0	8		0.015426	0.016515		Bacteria(100	Proteobacter	Alphaproteol	Rhizobiales(1	Bradyrhizobi	unclassified(100)
Otu01842	4	0	0	0	8		0.015426	0.016515		Bacteria(100	Proteobacter	Alphaproteol	Sphingomon	unclassified(	unclassified(75)
Otu01899	3	1	0	0	8		0			Bacteria(100	Proteobacter	Alphaproteol	Rhizobiales(1	Rhizobiaceae	unclassified(75)
Otu02567	3	1	0	0	8		0			Bacteria(100	Firmicutes(1	Clostridia(10	Clostridiales(	Peptostrepto	Incertae_Sedis(100)
Otu01440	0	1	1	4	8		0.015426			Bacteria(100	Chloroflexi(1	Gitt-GS-136(	unclassified(	unclassified(	unclassified(100)
Otu01404	0	0	2	4	8		0.015426			Bacteria(100	Proteobacter	Alphaproteol	Rhizobiales(1	unclassified(	unclassified(100)
Otu01437	0	0	2	4	8		0.015426			Bacteria(100	Proteobacter	Betaproteob	Burkholderia	Comamonad	Ramlibacter(100)
Otu01921	1	3	0	0	8	0				Bacteria(100	Proteobacter	Gammaprote	Pseudomona	Pseudomona	Pseudomonas(75)
Otu01272	1	1	0	3	7				0	Bacteria(100	Proteobacter	Alphaproteol	Rhizobiales(1	Hyphomicrol	unclassified(61)
Otu01319	1	1	0	3	7				0	Bacteria(100	Planctomyce	Planctomyce	Planctomyce	Planctomyce	Singulisphaera(61)
Otu02075	1	1	0	3	7				0	Bacteria(100	JL-ETNP-Z39(	unclassified(	unclassified(	unclassified(	unclassified(100)
Otu01385	2	0	0	3	7				0	Bacteria(100	Proteobacter	Alphaproteol	Sphingomon	Sphingomon	Sphingomonas(80)
Otu01487	2	0	0	3	7				0	Bacteria(100	Nitrospirae(1	Nitrospira(10	Nitrospirales	0319-6A21(1	unclassified(100)
Otu01299	0	1	0	5	7		0.006324		0.009016	Bacteria(100	Actinobacter	Acidimicrobii	Acidimicrobi	unclassified(	unclassified(100)
Otu01077	1	0	0	5	7		0.03709		0.009016	Bacteria(100	Proteobacter	Alphaproteol	Rhizobiales(1	Xanthobacte	Pseudolabrys(67)
Otu01779	1	0	0	5	7		0.03709		0.009016	Bacteria(100	Gemmatimo	Gemmatimo	Gemmatimo	Gemmatimo	unclassified(100)
Otu01476	0	0	1	5	7		0.006324		0.033997	Bacteria(100	Chloroflexi(1	Anaerolineae	Anaerolineal	Anaerolineac	unclassified(100)
Otu01691	0	1	0	5	7				0.048812	Bacteria(100	Acidobacteri	Acidobacteri	Subgroup_3(	Unknown_Fa	Bryobacter(100)
Otu01207	1	0	0	5	7				0.048812	Bacteria(100	Proteobacter	Alphaproteol	Rhizobiales(1	Xanthobacte	unclassified(84)
Otu01812	1	0	0	5	7				0.048812	Bacteria(100	Proteobacter	Betaproteob	unclassified(	unclassified(	unclassified(100)
Otu02257	1	0	0	5	7				0.048812	Bacteria(100	Gemmatimo	Gemmatimo	Gemmatimo	Gemmatimo	Gemmatimonas(100)
Otu01600	0	3	0	1	7	0		0		Bacteria(100	Actinobacter	Actinobacter	Frankiales(1	Acidotherma	Acidothermus(100)
Otu01568	3	0	0	1	7			0		Bacteria(100	Acidobacteri	Acidobacteri	Acidobacteri	Acidobacteri	unclassified(100)
Otu01844	3	0	0	1	7			0		Bacteria(100	Proteobacter	Alphaproteol	Rhizobiales(1	Rhizobiales_I	Bauldia(100)
Otu01942	3	0	0	1	7			0		Bacteria(100	Proteobacter	Alphaproteol	Sphingomon	Sphingomon	unclassified
Otu02417	3	0	0	1	7			0		Bacteria(100	Acidobacteri	Acidobacteri	Subgroup_6(	unclassified(	unclassified(100)
Otu02720	3	0	0	1	7			0		Bacteria(100	Proteobacter	Alphaproteol	Sphingomon	Sphingomon	Sphingomonas(75)
Otu01271	0	1	1	3	7		0			Bacteria(100	Proteobacter	Alphaproteol	Rhizobiales(1	Xanthobacte	Pseudolabrys(100)
Otu01491	0	1	1	3	7		0			Bacteria(100	Proteobacter	Betaproteob	Nitrosomona	Nitrosomona	unclassified(100)
Otu03102	0	1	1	3	7		0			Bacteria(100	Chloroflexi(1	S085(100)	unclassified(	unclassified(	unclassified(100)
Otu02112	0	0	2	3	7		0			Bacteria(100	Chloroflexi(1	Anaerolineae	Anaerolineal	Anaerolineac	unclassified(100)
Otu02247	0	0	3	0	6	0			0	Bacteria(100	Proteobacter	Gammaprote	unclassified(	unclassified(	unclassified(100)
Otu02328	0	0	3	0	6	0			0	Bacteria(100	unclassified(	unclassified(	unclassified(	unclassified(	unclassified(67)
Otu02376	0	0	3	0	6	0			0	Bacteria(100	Proteobacter	Alphaproteol	Rhizobiales(1	unclassified(	unclassified(67)
Otu02515	0	0	3	0	6	0			0	Bacteria(100	Proteobacter	Gammaprote	Pseudomona	Pseudomona	unclassified(67)
Otu02547	0	0	3	0	6	0			0	Bacteria(100	Cyanobacteri	unclassified(	unclassified(	unclassified(	unclassified(67)
Otu02564	0	0	3	0	6	0			0	Bacteria(100	Proteobacter	Betaproteob	Burkholderia	Comamonad	unclassified(100)
Otu02773	0	0	3	0	6	0			0	Bacteria(100	Cyanobacteri	unclassified(	unclassified(	unclassified(	unclassified(67)
Otu02900	0	0	3	0	6	0			0	Bacteria(100	unclassified(	unclassified(	unclassified(	unclassified(	unclassified(100)
Otu01380	1	0	0	4	6				0.01573	Bacteria(100	Chloroflexi(1	KD4-96(100)	unclassified(	unclassified(	unclassified(100)
Otu01394	1	0	0	4	6				0.01573	Bacteria(100	Chloroflexi(1	S085(100)	unclassified(	unclassified(	unclassified(100)
Otu01434	1	0	0	4	6				0.01573	Bacteria(100	Proteobacter	Alphaproteol	Rhizobiales(1	Xanthobacte	unclassified(100)
Otu02115	1	0	0	4	6				0.01573	Bacteria(100	Chloroflexi(1	JG30-KF-CM6	unclassified(	unclassified(	unclassified(100)
Otu02285	1	0	0	4	6				0.01573	Bacteria(100	Gemmatimo	Gemmatimo	Gemmatimo	Gemmatimo	Gemmatimonas(100)
Otu02290	3	0	0	0	6		0	0		Bacteria(100	Proteobacter	Alphaproteol	Rhizobiales(1	Hyphomicrol	unclassified(100)
Otu02622	3	0	0	0	6		0	0		Bacteria(100	Chloroflexi(1	Thermomicr	JG30-KF-CM4	unclassified(	unclassified(100)
Otu02686	3	0	0	0	6		0	0		Bacteria(100	Chloroflexi(1	S085(100)	unclassified(	unclassified(	unclassified(100)
Otu04287	3	0	0	0	6		0	0		Bacteria(100	Candidate_d	unclassified(	unclassified(	unclassified(	unclassified(100)
Otu02166	0	3	0	0	6	0		0		Bacteria(100	Proteobacter	Gammaprote	Xanthomona	Xanthomona	unclassified(100)
Otu01392	0	0	1	4	6		0.015426			Bacteria(100	Proteobacter	Alphaproteol	Sphingomon	Sphingomon	Sphingobium(61)
Otu01439	0	0	1	4	6		0.015426			Bacteria(100	Proteobacter	Betaproteob	Burkholderia	Oxalobactera	Massilia(61)
Otu02238	0	0	1	4	6		0.015426			Bacteria(100	Gemmatimo	Gemmatimo	Gemmatimo	Gemmatimo	Gemmatimonas(100)
Otu01582	1	0	0	3	5				0	Bacteria(100	Gemmatimo	Gemmatimo	Gemmatimo	Gemmatimo	Gemmatimonas(100)
Otu01611	1	0	0	3	5				0	Bacteria(100	Proteobacter	Alphaproteol	Sphingomon	unclassified(	unclassified(75)
Otu01750	1	0	0	3	5				0	Bacteria(100	Acidobacteri	Acidobacteri	Subgroup_3(	Unknown_Fa	Candidatus_Solibacter(100)
Otu01826	1	0	0	3	5				0	Bacteria(100	Proteobacter	Alphaproteol	Rhodospirilla	unclassified(	unclassified(100)
Otu01867	1	0	0	3	5				0	Bacteria(100	Acidobacteri	Acidobacteri	Subgroup_6(	unclassified(	unclassified(100)
Otu01383	0	0	0	5	5		0.006324		0.009016	Bacteria(100	Chloroflexi(1	KD4-96(100)	unclassified(	unclassified(	unclassified(100)
Otu01931	0	0	1	3	5		0			Bacteria(100	Proteobacter	Alphaproteol	Rhizobiales(1	DUNssu371(	unclassified(100)
Otu02172	0	0	1	3	5		0			Bacteria(100	Proteobacter	Betaproteob	unclassified(	unclassified(	unclassified(100)
Otu02201	0	0	1	3	5		0			Bacteria(100	Chloroflexi(1	Anaerolineae	Anaerolineal	Anaerolineac	unclassified(100)

Supplementary Table S7 List of the cell wall-degrading enzymes annotation for root gall-associated metagenome in CAZymes database

SequenceID	CAZy Familie	Subject EC	Enzymes	Lignocellulose-degrading enzymes	Pfam domains
Sphingoonadales_contig_674344_1_221_-	AA1	1.10.3.2	Cu-oxidase_2	Lignin-degrading enzymes	Cu-oxidase_2
Cytophagales_contig_229381_18_226_+	AA1		Cu-oxidase_2	Lignin-degrading enzymes	Cu-oxidase_2
Flavobacteriales_contig_29950_1_303_-	AA1		Cu-oxidase	Lignin-degrading enzymes	n/a
Flavobacteriales_contig_48518_1_274_-	AA1		Cu-oxidase_2	Lignin-degrading enzymes	Cu-oxidase_2
actinobacteria_contig_695320_1_233_+	AA1		Cu-oxidase	Lignin-degrading enzymes	n/a
otherBacteria_contig_413357_1_288_-	AA2	1.11.1.13/14	peroxidase	Lignin-degrading enzymes	peroxidase
otherBacteria_contig_101628_1_378_+	AA2		peroxidase	Lignin-degrading enzymes	peroxidase
Rhodocyclales_contig_53219_1_469_+	AA2		peroxidase	Lignin-degrading enzymes	peroxidase
actinobacteria_contig_557496_1_236_-	AA2		peroxidase	Lignin-degrading enzymes	peroxidase
Actinobacteria_sca664 size206_1_206_-	GH1		Beta-glucosidase	Oligosaccharide-degrading enzymes	Glyco_hydro_1
Bacteroidetes_sca1626 size293_1_293_-	GH1		Beta-glucosidase	Oligosaccharide-degrading enzymes	Polysacc_synt_2
Bacteroidetes_sca3914 size209_1_209_+	GH1		Beta-glucosidase	Oligosaccharide-degrading enzymes	Polysacc_synt_2
Bdellovibrionales_sca588 size318_1_318_-	GH1		Beta-glucosidase	Oligosaccharide-degrading enzymes	Polysacc_synt_2
Burkholderiales_sca1505 size346_1_346_-	GH1		Beta-glucosidase	Oligosaccharide-degrading enzymes	Polysacc_synt_2/Bac_transf
Burkholderiales_sca6474 size238_1_238_+	GH1		Beta-glucosidase	Oligosaccharide-degrading enzymes	Polysacc_synt_2
Cytophagales_sca2921 size252_1_163_-	GH1		Beta-glucosidase	Oligosaccharide-degrading enzymes	Polysacc_synt_2
Enterobacteriales_sca1032 size342_1_342_+	GH1		Beta-glucosidase	Oligosaccharide-degrading enzymes	PMI_typeI
Enterobacteriales_sca1141 size334_36_334_	GH1		Beta-glucosidase	Oligosaccharide-degrading enzymes	PMI_typeI
Enterobacteriales_sca1474 size317_1_317_+	GH1		Beta-glucosidase	Oligosaccharide-degrading enzymes	PMI_typeI
Enterobacteriales_sca1713 size306_1_306_-	GH1		Beta-glucosidase	Oligosaccharide-degrading enzymes	Glyco_hydro_1
Enterobacteriales_sca260 size472_1_280_-	GH1		Beta-glucosidase	Oligosaccharide-degrading enzymes	Glyco_hydro_1
Enterobacteriales_sca3336 size253_1_253_+	GH1		Beta-glucosidase	Oligosaccharide-degrading enzymes	Glyco_hydro_1
Enterobacteriales_sca3452 size251_103_251	GH1		Beta-glucosidase	Oligosaccharide-degrading enzymes	Glyco_hydro_1
Enterobacteriales_sca4095 size239_32_239_	GH1		Beta-glucosidase	Oligosaccharide-degrading enzymes	Glyco_hydro_1
Enterobacteriales_sca473 size407_1_407_+	GH1		Beta-glucosidase	Oligosaccharide-degrading enzymes	Glyco_hydro_1
Enterobacteriales_sca5066 size224_1_224_+	GH1		Beta-glucosidase	Oligosaccharide-degrading enzymes	Glyco_hydro_1
Enterobacteriales_sca5069 size224_1_224_+	GH1		Beta-glucosidase	Oligosaccharide-degrading enzymes	Glyco_hydro_1
Enterobacteriales_sca5215 size222_1_222_-	GH1		Beta-glucosidase	Oligosaccharide-degrading enzymes	Polysacc_synt_2
Enterobacteriales_sca5521 size217_1_217_-	GH1		Beta-glucosidase	Oligosaccharide-degrading enzymes	PMI_typeI
Enterobacteriales_sca5720 size215_1_215_-	GH1		Beta-glucosidase	Oligosaccharide-degrading enzymes	Glyco_hydro_1
Enterobacteriales_sca6490 size205_1_205_-	GH1		Beta-glucosidase	Oligosaccharide-degrading enzymes	Polysacc_synt_2
Firicutes_sca1466 size209_1_209_+	GH1		Beta-glucosidase	Oligosaccharide-degrading enzymes	Polysacc_synt_2
Firicutes_sca1485 size208_1_208_+	GH1		Beta-glucosidase	Oligosaccharide-degrading enzymes	Polysacc_synt_2
Flavobacteriales_sca109 size973_314_973_+	GH1		Beta-glucosidase	Oligosaccharide-degrading enzymes	Polysacc_synt_2
Flavobacteriales_sca11924 size212_1_212_-	GH1		Beta-glucosidase	Oligosaccharide-degrading enzymes	Polysacc_synt_2
Flavobacteriales_sca2298 size434_1_434_-	GH1		Beta-glucosidase	Oligosaccharide-degrading enzymes	Glyco_hydro_1
Flavobacteriales_sca3605 size365_1_365_+	GH1		Beta-glucosidase	Oligosaccharide-degrading enzymes	Polysacc_synt_2/Epimerase
Flavobacteriales_sca4073 size346_1_346_-	GH1		Beta-glucosidase	Oligosaccharide-degrading enzymes	Polysacc_synt_2
Flavobacteriales_sca500 size686_1_686_-	GH1		Beta-glucosidase	Oligosaccharide-degrading enzymes	Glyco_hydro_1
Flavobacteriales_sca682 size626_1_626_+	GH1		Beta-glucosidase	Oligosaccharide-degrading enzymes	Polysacc_synt_2
Flavobacteriales_sca9034 size244_1_244_-	GH1		Beta-glucosidase	Oligosaccharide-degrading enzymes	Polysacc_synt_2
OtherBacteria_sca1238 size368_1_368_-	GH1		Beta-glucosidase	Oligosaccharide-degrading enzymes	Polysacc_synt_2/Epimerase
OtherBacteria_sca1798 size329_1_329_+	GH1		Beta-glucosidase	Oligosaccharide-degrading enzymes	Polysacc_synt_2
OtherBacteria_sca254 size607_1_607_+	GH1		Beta-glucosidase	Oligosaccharide-degrading enzymes	Polysacc_synt_2
OtherBacteria_sca259 size603_1_435_-	GH1		Beta-glucosidase	Oligosaccharide-degrading enzymes	Polysacc_synt_2/Epimerase
OtherBacteria_sca4161 size254_1_254_-	GH1		Beta-glucosidase	Oligosaccharide-degrading enzymes	Polysacc_synt_2
OtherBacteria_sca6167 size225_1_225_-	GH1		Beta-glucosidase	Oligosaccharide-degrading enzymes	Polysacc_synt_2
Pseudoonadales_sca1895 size298_1_298_+	GH1		Beta-glucosidase	Oligosaccharide-degrading enzymes	Polysacc_synt_2
Pseudoonadales_sca5408 size215_1_215_-	GH1		Beta-glucosidase	Oligosaccharide-degrading enzymes	Polysacc_synt_2
Rhizobiales_sca3394 size263_1_263_-	GH1		Beta-glucosidase	Oligosaccharide-degrading enzymes	Polysacc_synt_2
Rhizobiales_sca4414 size246_1_246_+	GH1		Beta-glucosidase	Oligosaccharide-degrading enzymes	Glyco_hydro_1
Rhodocyclales_sca447 size252_1_252_-	GH1		Beta-glucosidase	Oligosaccharide-degrading enzymes	Polysacc_synt_2
Rhodocyclales_sca658 size211_1_211_+	GH1		Beta-glucosidase	Oligosaccharide-degrading enzymes	Polysacc_synt_2
Sphingobacter_sca10086 size248_1_248_+	GH1		Beta-glucosidase	Oligosaccharide-degrading enzymes	Glyco_hydro_1
Sphingobacter_sca10209 size247_1_247_+	GH1		Beta-glucosidase	Oligosaccharide-degrading enzymes	Polysacc_synt_2
Sphingobacter_sca10243 size247_1_247_-	GH1		Beta-glucosidase	Oligosaccharide-degrading enzymes	Glyco_hydro_1
Sphingobacter_sca10895 size242_1_242_+	GH1		Beta-glucosidase	Oligosaccharide-degrading enzymes	Polysacc_synt_2
Sphingobacter_sca11531 size237_1_237_-	GH1		Beta-glucosidase	Oligosaccharide-degrading enzymes	Glyco_hydro_1
Sphingobacter_sca12263 size232_1_232_+	GH1		Beta-glucosidase	Oligosaccharide-degrading enzymes	Polysacc_synt_2
Sphingobacter_sca6265 size291_1_291_+	GH1		Beta-glucosidase	Oligosaccharide-degrading enzymes	Polysacc_synt_2
Sphingobacter_sca7214 size279_1_279_+	GH1		Beta-glucosidase	Oligosaccharide-degrading enzymes	Polysacc_synt_2
Sphingobacter_sca7455 size276_1_276_-	GH1		Beta-glucosidase	Oligosaccharide-degrading enzymes	Polysacc_synt_2
Sphingobacter_sca8054 size269_1_269_+	GH1		Beta-glucosidase	Oligosaccharide-degrading enzymes	PMI_typeI
Sphingoonadales_sca4626 size211_62_211_-	GH1		Beta-glucosidase	Oligosaccharide-degrading enzymes	Polysacc_synt_2
Xanthoonadales_sca1178 size270_1_270_+	GH1		Beta-glucosidase	Oligosaccharide-degrading enzymes	Glyco_hydro_1
Xanthoonadales_sca861 size296_1_296_-	GH1		Beta-glucosidase	Oligosaccharide-degrading enzymes	Glyco_hydro_1
Bacteroidetes_sca4003 size207_1_207_-	GH10	3.2.1.8	Endo-1,4-beta-xylanase	xylanase	Glyco_hydro_10
Cytophagales_sca185 size551_1_551_+	GH10		Endo-1,4-beta-xylanase	xylanase	Glyco_hydro_10
Cytophagales_sca2261 size274_1_274_-	GH10		Endo-1,4-beta-xylanase	xylanase	Glyco_hydro_10
Rhizobiales_sca3939 size253_1_253_+	GH10		Endo-1,4-beta-xylanase	xylanase	Glyco_hydro_10
Sphingoonadales_sca4508 size213_1_213_+	GH10		Endo-1,4-beta-xylanase	xylanase	Glyco_hydro_10
Burkholderiales_sca1147 size370_1_370_-	GH13		Alpha-amylase	Amylase	Alpha-amylase
Cytophagales_sca4840 size204_1_204_-	GH13		Alpha-amylase	Amylase	Alpha-amylase
Enterobacteriales_sca1202 size332_1_332_+	GH13		Alpha-amylase	Amylase	Alpha-amylase
Enterobacteriales_sca3000 size261_1_261_-	GH13		Alpha-amylase	Amylase	Alpha-amylase
Enterobacteriales_sca6330 size207_1_207_-	GH13		Alpha-amylase	Amylase	Alpha-amylase
Enterobacteriales_sca6590 size204_1_204_+	GH13		Alpha-amylase	Amylase	Alpha-amylase
Flavobacteriales_sca1084 size556_1_556_+	GH13		Alpha-amylase	Amylase	Alpha-amylase
Flavobacteriales_sca1101 size553_1_553_+	GH13		Alpha-amylase	Amylase	Alpha-amylase
Flavobacteriales_sca2389 size428_1_428_+	GH13		Alpha-amylase	Amylase	Alpha-amylase
Flavobacteriales_sca344 size745_1_745_+	GH13		Alpha-amylase	Amylase	Alpha-amylase
Flavobacteriales_sca594 size651_1_651_-	GH13		Alpha-amylase	Amylase	Alpha-amylase
OtherBacteria_sca271 size593_1_593_+	GH13		Alpha-amylase	Amylase	Alpha-amylase
OtherBacteria_sca3215 size276_1_276_-	GH13	3.2.1.-3.2.1.10	Alpha-amylase	Amylase	Alpha-amylase

Rhizobiales_sca4450 size245_1_245_+	GH13		Alpha-amylase	Amylase	Alpha-amylase
Rhizobiales_sca7835 size209_1_209_-	GH13		Alpha-amylase	Amylase	Alpha-amylase
Rhizobiales_sca8304 size206_1_206_-	GH13		Alpha-amylase	Amylase	Alpha-amylase
Sphingobacter_sca1928 size408_1_408_+	GH13		Alpha-amylase	Amylase	Alpha-amylase
Sphingobacter_sca4571 size321_1_321_+	GH13		Alpha-amylase	Amylase	Alpha-amylase
Sphingoonadales_sca5014 size205_1_205_+	GH13		Alpha-amylase	Amylase	Alpha-amylase
Xanthoonadales_sca1529 size246_1_246_+	GH13		Alpha-amylase	Amylase	Alpha-amylase
Bacteroidetes_sca1771 size284_1_284_-	GH15		Glucan 1,4-alpha-glucosida	Amylase	Glyco_hydro_15
Pseudoonadales_sca1192 size339_1_339_+	GH15		Glucan 1,4-alpha-glucosida	Amylase	Glyco_hydro_15
Flavobacteriales_sca12067 size210_1_210_+	GH16		glucan endo-1,3-β-D-glucosidase		Glyco_hydro_16
Flavobacteriales_sca2157 size444_1_334_+	GH16		glucan endo-1,3-β-D-glucosidase		Glyco_hydro_16
OtherBacteria_sca782 size427_1_427_-	GH16		glucan endo-1,3-β-D-glucosidase		Glyco_hydro_16
Rhizobiales_sca4106 size250_1_250_-	GH16		glucan endo-1,3-β-D-glucosidase		Glyco_hydro_16
Enterobacteriales_sca6065 size211_1_211_-	GH18		chitinase	Chitinase	ChitinaseA_N
Enterobacteriales_sca771 size367_1_367_+	GH18		chitinase	Chitinase	Glyco_hydro_18
OtherBacteria_sca4832 size243_1_243_-	GH18		chitinase	Chitinase	Glyco_hydro_18
Sphingobacter_sca4850 size315_1_315_-	GH18		chitinase	Chitinase	Glyco_hydro_18
Bacteroidetes_sca1117 size328_1_328_-	GH2		Beta-galactosidase	Oligosaccharide-degrading enzymes	Glyco_hydro_2_N
Bacteroidetes_sca1423 size305_1_305_-	GH2		Beta-galactosidase	Oligosaccharide-degrading enzymes	Glyco_hydro_2_N
Bacteroidetes_sca1594 size294_1_294_-	GH2		Beta-galactosidase	Oligosaccharide-degrading enzymes	Glyco_hydro_2_C
Bacteroidetes_sca1988 size272_1_272_+	GH2	3.2.1.23	Beta-galactosidase	Oligosaccharide-degrading enzymes	Glyco_hydro_2_N
Bacteroidetes_sca2081 size267_1_267_-	GH2		Beta-galactosidase	Oligosaccharide-degrading enzymes	Glyco_hydro_2_C
Bacteroidetes_sca2305 size258_1_258_+	GH2		Beta-galactosidase	Oligosaccharide-degrading enzymes	Glyco_hydro_2_N
Bacteroidetes_sca2917 size235_1_235_-	GH2		Beta-galactosidase	Oligosaccharide-degrading enzymes	Glyco_hydro_2_C
Bacteroidetes_sca3779 size212_1_212_+	GH2		Beta-galactosidase	Oligosaccharide-degrading enzymes	Glyco_hydro_2_N
Bacteroidetes_sca3929 size208_1_208_+	GH2		Beta-galactosidase	Oligosaccharide-degrading enzymes	Glyco_hydro_2_N
Bacteroidetes_sca398 size448_1_448_-	GH2		Beta-galactosidase	Oligosaccharide-degrading enzymes	Glyco_hydro_2_C
Burkholderiales_sca638 size430_1_430_+	GH2		Beta-galactosidase	Oligosaccharide-degrading enzymes	Glyco_hydro_2_N
Cytophagales_sca3810 size225_1_225_+	GH2		Beta-galactosidase	Oligosaccharide-degrading enzymes	Glyco_hydro_2_N
Enterobacteriales_sca2007 size291_1_291_+	GH2		Beta-galactosidase	Oligosaccharide-degrading enzymes	Glyco_hydro_2_N
Enterobacteriales_sca3424 size251_1_251_+	GH2	3.2.1.23	Beta-galactosidase	Oligosaccharide-degrading enzymes	Glyco_hydro_2_C
Enterobacteriales_sca4396 size234_1_234_+	GH2	3.2.1.23	Beta-galactosidase	Oligosaccharide-degrading enzymes	Glyco_hydro_2_N
Enterobacteriales_sca6883 size201_1_201_+	GH2	3.2.1.23	Beta-galactosidase	Oligosaccharide-degrading enzymes	Glyco_hydro_2_N
Flavobacteriales_sca1874 size465_1_465_-	GH2		Beta-galactosidase	Oligosaccharide-degrading enzymes	Glyco_hydro_2_N
Flavobacteriales_sca9300 size241_1_241_+	GH2		Beta-galactosidase	Oligosaccharide-degrading enzymes	Glyco_hydro_2_N
OtherBacteria_sca3697 size264_1_264_+	GH2		Beta-galactosidase	Oligosaccharide-degrading enzymes	Glyco_hydro_2_C
OtherBacteria_sca5000 size241_1_241_-	GH2		Beta-galactosidase	Oligosaccharide-degrading enzymes	Glyco_hydro_2_C
Sphingobacter_sca1086 size473_1_473_-	GH2		Beta-galactosidase	Oligosaccharide-degrading enzymes	Glyco_hydro_2_C
Sphingobacter_sca12478 size231_1_231_+	GH2		Beta-galactosidase	Oligosaccharide-degrading enzymes	Glyco_hydro_2_C
Sphingobacter_sca16116 size210_1_210_-	GH2		Beta-galactosidase	Oligosaccharide-degrading enzymes	Glyco_hydro_2_C
Sphingobacter_sca203 size716_428_716_-	GH2		Beta-galactosidase	Oligosaccharide-degrading enzymes	Glyco_hydro_2_C
Sphingobacter_sca326 size640_1_640_+	GH2		Beta-galactosidase	Oligosaccharide-degrading enzymes	Glyco_hydro_2/DUF4038
Sphingobacter_sca364 size623_1_623_+	GH2		Beta-galactosidase	Oligosaccharide-degrading enzymes	Glyco_hydro_2_C
Sphingobacter_sca4108 size331_1_331_+	GH2		Beta-galactosidase	Oligosaccharide-degrading enzymes	Glyco_hydro_2_N
Sphingobacter_sca5154 size310_1_139_-	GH2		Beta-galactosidase	Oligosaccharide-degrading enzymes	Glyco_hydro_2_N
Sphingobacter_sca551 size563_1_311_+	GH2		Beta-galactosidase	Oligosaccharide-degrading enzymes	Glyco_hydro_2_C
Sphingobacter_sca6182 size293_1_293_-	GH2		Beta-galactosidase	Oligosaccharide-degrading enzymes	Glyco_hydro_2_C
Sphingoonadales_sca3076 size240_1_240_-	GH2		Beta-galactosidase	Oligosaccharide-degrading enzymes	Glyco_hydro_2_N
Bacteroidetes_sca2932 size235_1_235_+	GH20		Beta-N-acetylhexosaminida	Chitinase	Glyco_hydro_20
Bacteroidetes_sca3529 size218_1_218_-	GH20		Beta-N-acetylhexosaminida	Chitinase	Glyco_hydro_20
Bacteroidetes_sca3844 size210_1_210_-	GH20		Beta-N-acetylhexosaminida	Chitinase	Glyco_hydro_20
Cytophagales_sca2272 size274_1_274_+	GH20		Beta-N-acetylhexosaminida	Chitinase	Glyco_hydro_20
Enterobacteriales_sca5260 size221_1_221_-	GH20		Beta-N-acetylhexosaminida	Chitinase	Glyco_hydro_20
OtherBacteria_sca1960 size320_1_320_-	GH20		Beta-N-acetylhexosaminida	Chitinase	Glyco_hydro_20
OtherBacteria_sca3782 size262_1_262_+	GH20		Beta-N-acetylhexosaminida	Chitinase	Glyco_hydro_20
OtherBacteria_sca5526 size233_1_233_+	GH20		Beta-N-acetylhexosaminida	Chitinase	Glyco_hydro_20
Rhizobiales_sca6089 size226_1_226_-	GH20		Beta-N-acetylhexosaminida	Chitinase	Glyco_hydro_20
Rhizobiales_sca63 size696_230_547_-	GH20		Beta-N-acetylhexosaminida	Chitinase	Glyco_hydro_20
Sphingobacter_sca10167 size248_1_248_+	GH20		Beta-N-acetylhexosaminida	Chitinase	Glyco_hydro_20
Sphingobacter_sca12095 size234_122_234_-	GH20		Beta-N-acetylhexosaminida	Chitinase	Glyco_hydro_20
Sphingobacter_sca13636 size224_1_224_+	GH20		Beta-N-acetylhexosaminida	Chitinase	Glyco_hydro_20
Sphingobacter_sca14193 size221_1_221_+	GH20		Beta-N-acetylhexosaminida	Chitinase	Glyco_hydro_20
Sphingobacter_sca1540 size432_1_432_-	GH20		Beta-N-acetylhexosaminida	Chitinase	Glyco_hydro_20
Sphingobacter_sca16108 size210_1_210_-	GH20		Beta-N-acetylhexosaminida	Chitinase	Glyco_hydro_20
Sphingobacter_sca16614 size208_1_208_-	GH20		Beta-N-acetylhexosaminida	Chitinase	Glyco_hydro_20
Sphingobacter_sca2623 size375_1_375_-	GH20		Beta-N-acetylhexosaminida	Chitinase	Glyco_hydro_20
Sphingobacter_sca3883 size336_1_336_+	GH20		Beta-N-acetylhexosaminida	Chitinase	Glyco_hydro_20
Sphingobacter_sca4751 size317_1_317_+	GH20		Beta-N-acetylhexosaminida	Chitinase	Glyco_hydro_20
Sphingobacter_sca5852 size298_1_298_+	GH20		Beta-N-acetylhexosaminida	Chitinase	Glyco_hydro_20
Sphingobacter_sca6621 size287_1_287_+	GH20		Beta-N-acetylhexosaminida	Chitinase	Glyco_hydro_20
Sphingobacter_sca7002 size281_1_281_+	GH20		Beta-N-acetylhexosaminida	Chitinase	Glyco_hydro_20
Sphingobacter_sca9289 size256_1_256_+	GH20		Beta-N-acetylhexosaminida	Chitinase	Glyco_hydro_20
Xanthoonadales_sca120 size539_1_539_+	GH20		Beta-N-acetylhexosaminida	Chitinase	Glyco_hydro_20
Flavobacteriales_sca12929 size202_1_202_+	GH25		lysozyme		Glyco_hydro_25
Rhizobiales_sca173 size532_1_532_-	GH25		lysozyme		Glyco_hydro_25
Rhizobiales_sca6935 size217_1_217_+	GH25		lysozyme		Glyco_hydro_25
Rhizobiales_sca8626 size203_1_203_-	GH25		lysozyme		Glyco_hydro_25
Bacteroidetes_sca1251 size319_1_319_-	GH26		mannan endo-1,4-β-manno	Hemicellulase	Glyco_hydro_26
Enterobacteriales_sca4404 size234_1_234_+	GH26	3.2.1.78	mannan endo-1,4-β-manno	Hemicellulase	Glyco_hydro_26
Sphingobacter_sca8379 size265_1_265_-	GH26		mannan endo-1,4-β-manno	Hemicellulase	Glyco_hydro_26
Rhizobiales_sca128 size570_1_570_-	GH28		Polygalacturonase	pectin-degrading enzymes	Glyco_hydro_28
Sphingobacter_sca9274 size256_1_256_-	GH28		Polygalacturonase	pectin-degrading enzymes	Glyco_hydro_28
Bacteroidetes_sca1329 size313_102_313_-	GH3		β-glucosidase	Oligosaccharide-degrading enzymes	Glyco_hydro_3_C
Bacteroidetes_sca172 size542_1_542_+	GH3		β-glucosidase	Oligosaccharide-degrading enzymes	Glyco_hydro_3
Bacteroidetes_sca2596 size246_1_246_-	GH3		β-glucosidase	Oligosaccharide-degrading enzymes	Glyco_hydro_3_C
Bacteroidetes_sca554 size409_1_409_-	GH3		β-glucosidase	Oligosaccharide-degrading enzymes	Glyco_hydro_3_C

Bacteroidetes_sca736 size375_1_375_+	GH3		β-glucosidase	Oligosaccharide-degrading enzymes	Glyco_hydro_3
Burkholderiales_sca10961 size202_1_202_+	GH3		β-glucosidase	Oligosaccharide-degrading enzymes	Glyco_hydro_3_C
Burkholderiales_sca1989 size322_1_322_+	GH3		β-glucosidase	Oligosaccharide-degrading enzymes	Glyco_hydro_3
Burkholderiales_sca7161 size231_1_231_+	GH3		β-glucosidase	Oligosaccharide-degrading enzymes	Glyco_hydro_3
Burkholderiales_sca9028 size216_1_216_+	GH3		β-glucosidase	Oligosaccharide-degrading enzymes	Glyco_hydro_3
Cytophagales_sca1439 size320_1_320_+	GH3		β-glucosidase	Oligosaccharide-degrading enzymes	Glyco_hydro_3
Cytophagales_sca1819 size296_1_296_+	GH3		β-glucosidase	Oligosaccharide-degrading enzymes	Glyco_hydro_3
Enterobacteriales_sca688 size376_1_376_-	GH3		β-glucosidase	Oligosaccharide-degrading enzymes	Glyco_hydro_3
Enterobacteriales_sca92 size575_1_575_+	GH3		β-glucosidase	Oligosaccharide-degrading enzymes	Glyco_hydro_3_C
Flavobacteriales_sca11664 size214_1_214_-	GH3		β-glucosidase	Oligosaccharide-degrading enzymes	Glyco_hydro_3_C
Flavobacteriales_sca1437 size505_1_505_-	GH3		β-glucosidase	Oligosaccharide-degrading enzymes	Glyco_hydro_3
Flavobacteriales_sca1648 size484_1_484_-	GH3		β-glucosidase	Oligosaccharide-degrading enzymes	Glyco_hydro_3
Flavobacteriales_sca1864 size466_1_466_+	GH3		β-glucosidase	Oligosaccharide-degrading enzymes	Glyco_hydro_3
Flavobacteriales_sca2703 size407_1_407_+	GH3		β-glucosidase	Oligosaccharide-degrading enzymes	Glyco_hydro_3
Flavobacteriales_sca453 size705_1_705_-	GH3		β-glucosidase	Oligosaccharide-degrading enzymes	Glyco_hydro_3/Glyco_hydro_3_C
Flavobacteriales_sca5192 size314_1_314_+	GH3		β-glucosidase	Oligosaccharide-degrading enzymes	Glyco_hydro_3_C
Flavobacteriales_sca571 size659_1_659_+	GH3		β-glucosidase	Oligosaccharide-degrading enzymes	Glyco_hydro_3
Flavobacteriales_sca665 size632_1_632_+	GH3		β-glucosidase	Oligosaccharide-degrading enzymes	Glyco_hydro_3
Flavobacteriales_sca81 size1040_1_1039_+	GH3		β-glucosidase	Oligosaccharide-degrading enzymes	Glyco_hydro_3/Glyco_hydro_3_C
Flavobacteriales_sca9457 size239_1_239_-	GH3		β-glucosidase	Oligosaccharide-degrading enzymes	Glyco_hydro_3_C
Flavobacteriales_sca9684 size236_1_236_+	GH3		β-glucosidase	Oligosaccharide-degrading enzymes	Glyco_hydro_3
OtherBacteria_sca3012 size282_1_282_-	GH3		β-glucosidase	Oligosaccharide-degrading enzymes	Glyco_hydro_3
OtherBacteria_sca5386 size235_1_235_+	GH3		β-glucosidase	Oligosaccharide-degrading enzymes	Glyco_hydro_3
OtherBacteria_sca8263 size203_1_203_-	GH3		β-glucosidase	Oligosaccharide-degrading enzymes	Glyco_hydro_3_C
Pseudoonadales_sca3742 size243_115_243_	GH3		β-glucosidase	Oligosaccharide-degrading enzymes	Glyco_hydro_3_C
Pseudoonadales_sca6327 size202_1_202_-	GH3		β-glucosidase	Oligosaccharide-degrading enzymes	Glyco_hydro_3
Pseudoonadales_sca768 size382_1_382_+	GH3		β-glucosidase	Oligosaccharide-degrading enzymes	Glyco_hydro_3
Sphingobacter_sca10005 size249_1_249_+	GH3		β-glucosidase	Oligosaccharide-degrading enzymes	Glyco_hydro_3_C
Sphingobacter_sca1101 size472_27_472_+	GH3		β-glucosidase	Oligosaccharide-degrading enzymes	Glyco_hydro_3
Sphingobacter_sca11816 size235_1_235_+	GH3		β-glucosidase	Oligosaccharide-degrading enzymes	Glyco_hydro_3_C
Sphingobacter_sca15155 size215_1_215_+	GH3		β-glucosidase	Oligosaccharide-degrading enzymes	Glyco_hydro_3
Sphingobacter_sca15225 size215_1_215_+	GH3		β-glucosidase	Oligosaccharide-degrading enzymes	Glyco_hydro_3
Sphingobacter_sca15714 size212_1_212_-	GH3		β-glucosidase	Oligosaccharide-degrading enzymes	Glyco_hydro_3
Sphingobacter_sca254 size676_1_663_+	GH3		β-glucosidase	Oligosaccharide-degrading enzymes	Glyco_hydro_3
Sphingobacter_sca5031 size312_1_312_+	GH3		β-glucosidase	Oligosaccharide-degrading enzymes	Glyco_hydro_3
Sphingobacter_sca9138 size258_1_258_-	GH3		β-glucosidase	Oligosaccharide-degrading enzymes	Glyco_hydro_3
Sphingoonadales_sca1659 size286_1_286_-	GH3		β-glucosidase	Oligosaccharide-degrading enzymes	Glyco_hydro_3
Sphingoonadales_sca2416 size257_1_257_+	GH3		β-glucosidase	Oligosaccharide-degrading enzymes	Glyco_hydro_3
Sphingoonadales_sca3474 size232_11_232_-	GH3		β-glucosidase	Oligosaccharide-degrading enzymes	Glyco_hydro_3_C
Sphingoonadales_sca44 size605_1_605_+	GH3		β-glucosidase	Oligosaccharide-degrading enzymes	Glyco_hydro_3
Sphingoonadales_sca4871 size207_1_207_+	GH3		β-glucosidase	Oligosaccharide-degrading enzymes	Glyco_hydro_3
Sphingoonadales_sca495 size386_1_386_-	GH3		β-glucosidase	Oligosaccharide-degrading enzymes	Glyco_hydro_3
Sphingoonadales_sca648 size363_1_363_-	GH3		β-glucosidase	Oligosaccharide-degrading enzymes	Glyco_hydro_3
Xanthoonadales_sca2634 size202_1_202_-	GH3		β-glucosidase	Oligosaccharide-degrading enzymes	Glyco_hydro_3
Xanthoonadales_sca416 size365_1_365_+	GH3		β-glucosidase	Oligosaccharide-degrading enzymes	Glyco_hydro_3
Burkholderiales_sca4180 size268_1_268_+	GH30		beta-glucosidase	Oligosaccharide-degrading enzymes	Glyco_hydro_30
Cytophagales_sca108 size630_1_630_+	GH30		beta-glucosidase	Oligosaccharide-degrading enzymes	Glyco_hydro_30
Flavobacteriales_sca4251 size340_1_340_-	GH30		beta-glucosidase	Oligosaccharide-degrading enzymes	Glyco_hydro_30
Flavobacteriales_sca6548 size284_1_284_-	GH30		beta-glucosidase	Oligosaccharide-degrading enzymes	Glyco_hydro_30
Flavobacteriales_sca6794 size279_1_279_+	GH30		beta-glucosidase	Oligosaccharide-degrading enzymes	Glyco_hydro_30
OtherBacteria_sca4357 size251_1_251_-	GH30		beta-glucosidase	Oligosaccharide-degrading enzymes	Glyco_hydro_30
OtherBacteria_sca6509 size221_1_221_+	GH30		beta-glucosidase	Oligosaccharide-degrading enzymes	Glyco_hydro_30
Sphingobacter_sca17 size1257_1_1256_+	GH30		beta-glucosidase	Oligosaccharide-degrading enzymes	Glyco_hydro_30
Burkholderiales_sca10194 size207_1_207_+	GH31		alpha-glucosidase	Oligosaccharide-degrading enzymes	Glyco_hydro_31
Burkholderiales_sca2095 size319_1_319_-	GH31		alpha-glucosidase	Oligosaccharide-degrading enzymes	Glyco_hydro_31
Caulobacterales_sca3008 size205_1_205_-	GH31		alpha-glucosidase	Oligosaccharide-degrading enzymes	Glyco_hydro_31
Rhizobiales_sca2905 size273_1_273_+	GH31		alpha-glucosidase	Oligosaccharide-degrading enzymes	Glyco_hydro_31
Rhizobiales_sca2945 size272_1_272_-	GH31		alpha-glucosidase	Oligosaccharide-degrading enzymes	Glyco_hydro_31
Sphingobacter_sca10963 size241_1_176_-	GH31		alpha-glucosidase	Oligosaccharide-degrading enzymes	Glyco_hydro_31
Sphingobacter_sca12401 size231_1_187_-	GH31		alpha-glucosidase	Oligosaccharide-degrading enzymes	Glyco_hydro_31
Sphingobacter_sca12888 size228_1_228_-	GH31		alpha-glucosidase	Oligosaccharide-degrading enzymes	Glyco_hydro_31
Sphingobacter_sca18065 size201_1_201_-	GH31		alpha-glucosidase	Oligosaccharide-degrading enzymes	Glyco_hydro_31
Sphingobacter_sca3030 size361_1_361_+	GH31		alpha-glucosidase	Oligosaccharide-degrading enzymes	Glyco_hydro_31
Sphingobacter_sca376 size618_1_618_+	GH31		alpha-glucosidase	Oligosaccharide-degrading enzymes	Glyco_hydro_31
Sphingobacter_sca5337 size306_1_306_-	GH31		alpha-glucosidase	Oligosaccharide-degrading enzymes	Glyco_hydro_31
Sphingobacter_sca8659 size262_1_262_+	GH31		alpha-glucosidase	Oligosaccharide-degrading enzymes	Glyco_hydro_31
Sphingobacter_sca9790 size251_1_251_+	GH31		alpha-glucosidase	Oligosaccharide-degrading enzymes	Glyco_hydro_31
Xanthoonadales_sca385 size377_1_377_-	GH31		alpha-glucosidase	Oligosaccharide-degrading enzymes	Glyco_hydro_31
Xanthoonadales_sca768 size306_1_306_+	GH31		alpha-glucosidase	Oligosaccharide-degrading enzymes	Glyco_hydro_31
Sphingobacter_sca174 size749_1_749_-	GH31/GH17		alpha-glucosidase	Oligosaccharide-degrading enzymes	Glyco_hydro_31/MFS_2
Enterobacteriales_sca2828 size265_1_265_-	GH32		β-fructofuranosidase	Oligosaccharide-degrading enzymes	Glyco_hydro_32N
Enterobacteriales_sca4682 size230_1_230_-	GH32		β-fructofuranosidase	Oligosaccharide-degrading enzymes	Glyco_hydro_32N
Pseudoonadales_sca2182 size286_1_286_-	GH32		β-fructofuranosidase	Oligosaccharide-degrading enzymes	Glyco_hydro_32N
Sphingobacter_sca1717 size421_1_421_+	GH32		β-fructofuranosidase	Oligosaccharide-degrading enzymes	Glyco_hydro_32N
Caulobacterales_sca3022 size205_1_205_-	GH35		β-galactosidase	Oligosaccharide-degrading enzymes	Glyco_hydro_35
otherBacteria_contig_9691_1_230_+	GH37	3.2.1.28	Trehalase	Oligosaccharide-degrading enzymes	Trehalase
Enterobacteriales_contig_44150_11_251_-	GH37		Trehalase	Oligosaccharide-degrading enzymes	Trehalase
Enterobacteriales_contig_100823_1_209_-	GH37		Trehalase	Oligosaccharide-degrading enzymes	Trehalase
Enterobacteriales_contig_225116_1_273_+	GH37		Trehalase	Oligosaccharide-degrading enzymes	Trehalase
otherBacteria_contig_722138_1_210_-	GH37		Trehalase	Oligosaccharide-degrading enzymes	Trehalase
Bacteroidetes_contig_253855_1_313_-	GH37		Trehalase	Oligosaccharide-degrading enzymes	Trehalase
Xanthoonadales_contig_82636_1_240_+	GH37		Trehalase	Oligosaccharide-degrading enzymes	Trehalase
Enterobacteriales_sca545 size395_1_395_+	GH38		mannosidase	Oligosaccharide-degrading enzymes	Glyco_hydro_38
OtherBacteria_sca2677 size292_1_292_-	GH38		mannosidase	Oligosaccharide-degrading enzymes	Glyco_hydro_38
Rhizobiales_sca7674 size210_1_210_-	GH38		mannosidase	Oligosaccharide-degrading enzymes	Glyco_hydro_38C
Caulobacterales_sca1761 size238_1_238_-	GH39	3.2.1.37	α,α-trehalase	Oligosaccharide-degrading enzymes	Glyco_hydro_39

Caulobacterales_sca340 size363_1_363_-	GH39		α,α-trehalase	Oligosaccharide-degrading enzymes	Glyco_hydro_39
Rhizobiales_sca2423 size286_1_286_+	GH39		α,α-trehalase	Oligosaccharide-degrading enzymes	Glyco_hydro_39
Rhizobiales_sca5463 size233_1_233_+	GH39		α,α-trehalase	Oligosaccharide-degrading enzymes	Glyco_hydro_39
Enterobacteriales_sca3763 size245_1_245_-	GH4		β-galactosidase	Oligosaccharide-degrading enzymes	Glyco_hydro_4
Enterobacteriales_sca4311 size235_1_235_+	GH4		β-galactosidase	Oligosaccharide-degrading enzymes	Glyco_hydro_4
Enterobacteriales_sca5118 size223_1_223_-	GH4		β-galactosidase	Oligosaccharide-degrading enzymes	Glyco_hydro_4
Enterobacteriales_sca5356 size220_1_220_+	GH4		β-galactosidase	Oligosaccharide-degrading enzymes	Glyco_hydro_4
Enterobacteriales_sca6880 size201_1_201_-	GH4		β-galactosidase	Oligosaccharide-degrading enzymes	Glyco_hydro_4
Enterobacteriales_sca747 size370_1_370_-	GH4		β-galactosidase	Oligosaccharide-degrading enzymes	Glyco_hydro_4
Enterobacteriales_sca76 size601_1_476_-	GH4		β-galactosidase	Oligosaccharide-degrading enzymes	Glyco_hydro_4C
Rhizobiales_sca1150 size343_1_343_-	GH4		β-galactosidase	Oligosaccharide-degrading enzymes	Glyco_hydro_4
Rhizobiales_sca3335 size264_1_264_+	GH4		β-galactosidase	Oligosaccharide-degrading enzymes	Glyco_hydro_4C
Rhizobiales_sca3918 size254_1_254_+	GH4		β-galactosidase	Oligosaccharide-degrading enzymes	Glyco_hydro_4
Rhizobiales_sca620 size397_145_397_+	GH4	3.2.1.22	β-galactosidase	Oligosaccharide-degrading enzymes	Glyco_hydro_4
Rhizobiales_sca7291 size214_1_214_+	GH4		β-galactosidase	Oligosaccharide-degrading enzymes	Glyco_hydro_4C
Rhizobiales_sca8270 size206_1_206_+	GH4		β-galactosidase	Oligosaccharide-degrading enzymes	Glyco_hydro_4C
Enterobacteriales_sca110 size563_1_563_+	GH42		β-galactosidase	Oligosaccharide-degrading enzymes	Glyco_hydro_42M
Enterobacteriales_sca6178 size209_1_209_-	GH42		β-galactosidase	Oligosaccharide-degrading enzymes	Glyco_hydro_42M
Bacteroidetes_sca1705 size288_1_288_-	GH43	3.2.1.373.2.1.5	arabinanase	Oligosaccharide-degrading enzymes	Glyco_hydro_43
Bacteroidetes_sca1827 size280_1_280_+	GH43		arabinanase	Oligosaccharide-degrading enzymes	Xylanase
Bacteroidetes_sca1901 size277_1_277_+	GH43		arabinanase	Oligosaccharide-degrading enzymes	Glyco_hydro_43
Bacteroidetes_sca3541 size218_1_218_-	GH43		arabinanase	Oligosaccharide-degrading enzymes	Glyco_hydro_43
Bacteroidetes_sca498 size423_1_387_+	GH43		arabinanase	Oligosaccharide-degrading enzymes	Xylanase
Bacteroidetes_sca503 size422_1_422_+	GH43		arabinanase	Oligosaccharide-degrading enzymes	Glyco_hydro_43
Bacteroidetes_sca826 size362_1_362_+	GH43		arabinanase	Oligosaccharide-degrading enzymes	Xylanase
Caulobacterales_sca2206 size225_1_225_-	GH43		arabinanase	Oligosaccharide-degrading enzymes	Glyco_hydro_43
Enterobacteriales_sca1910 size296_1_296_-	GH43		arabinanase	Oligosaccharide-degrading enzymes	Glyco_hydro_43
Enterobacteriales_sca4537 size232_1_232_-	GH43		arabinanase	Oligosaccharide-degrading enzymes	Glyco_hydro_43
Enterobacteriales_sca626 size384_1_199_+	GH43		arabinanase	Oligosaccharide-degrading enzymes	Glyco_hydro_43
OtherBacteria_sca379 size539_1_539_-	GH43		arabinanase	Oligosaccharide-degrading enzymes	Glyco_hydro_43
OtherBacteria_sca7477 size211_1_211_+	GH43		arabinanase	Oligosaccharide-degrading enzymes	Glyco_hydro_43
Rhizobiales_sca5952 size227_1_227_-	GH43		arabinanase	Oligosaccharide-degrading enzymes	Glyco_hydro_43
Sphingobacter_sca10016 size249_1_249_-	GH43		arabinanase	Oligosaccharide-degrading enzymes	Glyco_hydro_43
Sphingobacter_sca1017 size482_1_482_+	GH43		arabinanase	Oligosaccharide-degrading enzymes	Glyco_hydro_43
Sphingobacter_sca2374 size385_155_385_+	GH43		arabinanase	Oligosaccharide-degrading enzymes	Glyco_hydro_43
Sphingobacter_sca3879 size336_1_336_+	GH43		arabinanase	Oligosaccharide-degrading enzymes	Glyco_hydro_43
Sphingobacter_sca4929 size314_1_314_+	GH43		arabinanase	Oligosaccharide-degrading enzymes	Glyco_hydro_43
Flavobacteriales_sca12857 size203_1_203_-	GH5		Cellulase	Cellulase	Cellulase-like
Sphingobacteriales_contig_82292_1_260_-	GH51		Alpha-L-arabinofuranosidas	Oligosaccharide-degrading enzymes	Alpha-L-AF_C
Firicutes_sca94 size374_1_374_+	GH52		xylosidase	Oligosaccharide-degrading enzymes	Glyco_hydro_52
Enterobacteriales_sca520 size400_1_400_+	GH53		endo-1,4- beta-galactanases	Hemicellulase	Glyco_hydro_53
Enterobacteriales_sca6125 size210_1_210_+	GH53		endo-1,4- beta-galactanases	Hemicellulase	Glyco_hydro_53
Bacteroidetes_sca1284 size317_1_317_-	GH67		Alpha-glucuronidases	Hemicellulase	Glyco_hydro_67M
Bacteroidetes_sca2787 size239_15_239_+	GH67		Alpha-glucuronidases	Hemicellulase	Glyco_hydro_67M
Bacteroidetes_sca3681 size214_1_214_+	GH67		Alpha-glucuronidases	Hemicellulase	Glyco_hydro_67M
Bacteroidetes_sca585 size402_1_402_-	GH67		Alpha-glucuronidases	Hemicellulase	Glyco_hydro_67M
Bacteroidetes_sca872 size356_1_356_+	GH67		Alpha-glucuronidases	Hemicellulase	Glyco_hydro_67C
Caulobacterales_sca387 size354_1_354_+	GH67	3.2.1.-	Alpha-glucuronidases	Hemicellulase	Glyco_hydro_67M
Cytophagales_sca2592 size262_1_262_-	GH67		Alpha-glucuronidases	Hemicellulase	Glyco_hydro_67C
Flavobacteriales_sca12638 size205_1_205_+	GH67		Alpha-glucuronidases	Hemicellulase	Glyco_hydro_67M
Flavobacteriales_sca5704 size301_55_301_-	GH67		Alpha-glucuronidases	Hemicellulase	Glyco_hydro_67M
Xanthoonadales_sca117 size540_1_540_+	GH67		Alpha-glucuronidases	Hemicellulase	Glyco_hydro_67M
Xanthoonadales_sca2175 size218_1_218_+	GH67		Alpha-glucuronidases	Hemicellulase	Glyco_hydro_67M
Bacteroidetes_sca1274 size317_1_317_+	GH76		alpha-1,6-mannanases	Oligosaccharide-degrading enzymes	Glyco_hydro_76
otherBacteria_contig_455872_1_236_-	GH78		α-L-rhamnosidase	Oligosaccharide-degrading enzymes	Bac_rhamnosid
Rhizobiales_contig_621013_1_318_-	GH78		α-L-rhamnosidase	Oligosaccharide-degrading enzymes	Bac_rhamnosid_N
otherBacteria_contig_188588_1_245_+	GH78		α-L-rhamnosidase	Oligosaccharide-degrading enzymes	Bac_rhamnosid
Caulobacterales_contig_166440_1_283_+	GH78		α-L-rhamnosidase	Oligosaccharide-degrading enzymes	Bac_rhamnosid
Bacteroidetes_contig_401319_1_334_-	GH78		α-L-rhamnosidase	Oligosaccharide-degrading enzymes	Bac_rhamnosid
Bacteroidetes_contig_55773_1_330_-	GH78		α-L-rhamnosidase	Oligosaccharide-degrading enzymes	Bac_rhamnosid_N
Cytophagales_contig_337090_1_221_+	GH78		α-L-rhamnosidase	Oligosaccharide-degrading enzymes	Bac_rhamnosid
Caulobacterales_contig_420289_1_201_+	GH78		α-L-rhamnosidase	Oligosaccharide-degrading enzymes	Bac_rhamnosid
Cytophagales_contig_720974_1_219_+	GH78		α-L-rhamnosidase	Oligosaccharide-degrading enzymes	Bac_rhamnosid
actinobacteria_contig_196689_1_256_+	GH78		α-L-rhamnosidase	Oligosaccharide-degrading enzymes	Bac_rhamnosid
Sphingobacteriales_contig_226000_1_269_+	GH78		α-L-rhamnosidase	Oligosaccharide-degrading enzymes	Bac_rhamnosid
Sphingobacteriales_contig_207200_1_259_+	GH78		α-L-rhamnosidase	Oligosaccharide-degrading enzymes	Bac_rhamnosid
actinobacteria_contig_250643_1_229_-	GH78		α-L-rhamnosidase	Oligosaccharide-degrading enzymes	Bac_rhamnosid
Flavobacteriales_contig_317800_1_301_-	GH78		α-L-rhamnosidase	Oligosaccharide-degrading enzymes	Bac_rhamnosid
Sphingobacteriales_contig_663719_1_220_-	GH78		α-L-rhamnosidase	Oligosaccharide-degrading enzymes	Bac_rhamnosid_N
Sphingobacteriales_contig_265566_1_375_-	GH78		α-L-rhamnosidase	Oligosaccharide-degrading enzymes	Bac_rhamnosid
Sphingobacteriales_contig_159767_1_132_+	GH78		α-L-rhamnosidase	Oligosaccharide-degrading enzymes	Bac_rhamnosid_N
Enterobacteriales_sca2037 size290_1_290_-	GH8		Cellulase	Cellulase	Glyco_hydro_8
Cytophagales_sca267 size507_109_507_-	GH9		Cellulase	Cellulase	Glyco_hydro_9
OtherBacteria_sca2358 size303_1_303_+	GH9		Cellulase	Cellulase	Glyco_hydro_9
Sphingobacter_sca4904 size314_1_314_-	GH9		Cellulase	Cellulase	Glyco_hydro_9
Bacteroidetes_sca1259 size319_1_319_+	GH92		alpha-1,2-mannosidases	Oligosaccharide-degrading enzymes	Glyco_hydro_92
Bacteroidetes_sca1631 size292_1_292_-	GH92		alpha-1,2-mannosidases	Oligosaccharide-degrading enzymes	Glyco_hydro_92
Bacteroidetes_sca210 size519_369_519_+	GH92		alpha-1,2-mannosidases	Oligosaccharide-degrading enzymes	Glyco_hydro_92
Bacteroidetes_sca2197 size262_1_262_+	GH92		alpha-1,2-mannosidases	Oligosaccharide-degrading enzymes	Glyco_hydro_92
Bacteroidetes_sca2231 size261_1_261_-	GH92		alpha-1,2-mannosidases	Oligosaccharide-degrading enzymes	Glyco_hydro_92
Bacteroidetes_sca2252 size260_1_260_-	GH92		alpha-1,2-mannosidases	Oligosaccharide-degrading enzymes	Glyco_hydro_92
Bacteroidetes_sca2429 size253_1_253_+	GH92		alpha-1,2-mannosidases	Oligosaccharide-degrading enzymes	Glyco_hydro_92
Bacteroidetes_sca303 size476_1_476_-	GH92		alpha-1,2-mannosidases	Oligosaccharide-degrading enzymes	Glyco_hydro_92
Bacteroidetes_sca3454 size220_1_220_+	GH92		alpha-1,2-mannosidases	Oligosaccharide-degrading enzymes	Glyco_hydro_92
Bacteroidetes_sca361 size457_1_457_+	GH92		alpha-1,2-mannosidases	Oligosaccharide-degrading enzymes	Glyco_hydro_92
Bacteroidetes_sca388 size450_1_450_-	GH92		alpha-1,2-mannosidases	Oligosaccharide-degrading enzymes	Glyco_hydro_92

Bacteroidetes_sca3995 size207_1_207_-	GH92		alpha-1,2-mannosidases	Oligosaccharide-degrading enzymes	Glyco_hydro_92
Bacteroidetes_sca4123 size204_1_204_-	GH92		alpha-1,2-mannosidases	Oligosaccharide-degrading enzymes	Glyco_hydro_92
Bacteroidetes_sca427 size439_1_403_+	GH92		alpha-1,2-mannosidases	Oligosaccharide-degrading enzymes	Glyco_hydro_92
Bacteroidetes_sca440 size435_1_435_-	GH92		alpha-1,2-mannosidases	Oligosaccharide-degrading enzymes	Glyco_hydro_92
Bacteroidetes_sca512 size419_1_419_+	GH92		alpha-1,2-mannosidases	Oligosaccharide-degrading enzymes	Glyco_hydro_92
Bacteroidetes_sca840 size360_1_360_-	GH92		alpha-1,2-mannosidases	Oligosaccharide-degrading enzymes	Glyco_hydro_92
Bacteroidetes_sca96 size617_1_342_+	GH92		alpha-1,2-mannosidases	Oligosaccharide-degrading enzymes	Glyco_hydro_92
Cytophagales_sca2691 size259_1_259_+	GH92	3.2.1.-	alpha-1,2-mannosidases	Oligosaccharide-degrading enzymes	Glyco_hydro_92
Cytophagales_sca678 size399_1_399_+	GH92		alpha-1,2-mannosidases	Oligosaccharide-degrading enzymes	Glyco_hydro_92
Flavobacteriales_sca12814 size203_1_203_+	GH92		alpha-1,2-mannosidases	Oligosaccharide-degrading enzymes	Glyco_hydro_92
Flavobacteriales_sca12898 size202_1_202_+	GH92		alpha-1,2-mannosidases	Oligosaccharide-degrading enzymes	Glyco_hydro_92
Flavobacteriales_sca7155 size272_1_272_+	GH92		alpha-1,2-mannosidases	Oligosaccharide-degrading enzymes	Glyco_hydro_92
Flavobacteriales_sca7348 size269_1_269_+	GH92		alpha-1,2-mannosidases	Oligosaccharide-degrading enzymes	Glyco_hydro_92
Flavobacteriales_sca7561 size266_1_266_-	GH92		alpha-1,2-mannosidases	Oligosaccharide-degrading enzymes	Glyco_hydro_92
Flavobacteriales_sca9860 size234_1_234_-	GH92		alpha-1,2-mannosidases	Oligosaccharide-degrading enzymes	Glyco_hydro_92
OtherBacteria_sca5435 size234_1_234_+	GH92		alpha-1,2-mannosidases	Oligosaccharide-degrading enzymes	Glyco_hydro_92
Sphingobacter_sca11202 size240_1_240_+	GH92		alpha-1,2-mannosidases	Oligosaccharide-degrading enzymes	Glyco_hydro_92
Sphingobacter_sca12291 size232_1_232_+	GH92		alpha-1,2-mannosidases	Oligosaccharide-degrading enzymes	Glyco_hydro_92
Sphingobacter_sca12308 size232_1_232_+	GH92		alpha-1,2-mannosidases	Oligosaccharide-degrading enzymes	Glyco_hydro_92
Sphingobacter_sca12991 size228_1_228_-	GH92		alpha-1,2-mannosidases	Oligosaccharide-degrading enzymes	Glyco_hydro_92
Sphingobacter_sca1345 size449_1_346_+	GH92		alpha-1,2-mannosidases	Oligosaccharide-degrading enzymes	Glyco_hydro_92
Sphingobacter_sca13550 size224_1_224_-	GH92		alpha-1,2-mannosidases	Oligosaccharide-degrading enzymes	Glyco_hydro_92
Sphingobacter_sca15147 size215_1_215_+	GH92		alpha-1,2-mannosidases	Oligosaccharide-degrading enzymes	Glyco_hydro_92
Sphingobacter_sca15187 size215_1_215_-	GH92		alpha-1,2-mannosidases	Oligosaccharide-degrading enzymes	Glyco_hydro_92
Sphingobacter_sca15947 size211_1_211_-	GH92		alpha-1,2-mannosidases	Oligosaccharide-degrading enzymes	Glyco_hydro_92
Sphingobacter_sca17473 size204_1_204_-	GH92		alpha-1,2-mannosidases	Oligosaccharide-degrading enzymes	Glyco_hydro_92
Sphingobacter_sca4331 size326_1_326_+	GH92		alpha-1,2-mannosidases	Oligosaccharide-degrading enzymes	Glyco_hydro_92
Sphingobacter_sca4382 size325_219_325_+	GH92		alpha-1,2-mannosidases	Oligosaccharide-degrading enzymes	Glyco_hydro_92
Sphingobacter_sca5651 size301_1_301_+	GH92		alpha-1,2-mannosidases	Oligosaccharide-degrading enzymes	Glyco_hydro_92
Sphingobacter_sca602 size550_168_550_-	GH92		alpha-1,2-mannosidases	Oligosaccharide-degrading enzymes	Glyco_hydro_92
Sphingobacter_sca6942 size282_1_282_-	GH92		alpha-1,2-mannosidases	Oligosaccharide-degrading enzymes	Glyco_hydro_92
Sphingobacter_sca9761 size251_1_251_-	GH92		alpha-1,2-mannosidases	Oligosaccharide-degrading enzymes	Glyco_hydro_92
Xanthoonadales_sca1055 size279_1_279_-	GH92		alpha-1,2-mannosidases	Oligosaccharide-degrading enzymes	Glyco_hydro_92
Xanthoonadales_sca1406 size254_1_254_+	GH92		alpha-1,2-mannosidases	Oligosaccharide-degrading enzymes	Glyco_hydro_92
Xanthoonadales_sca2607 size203_1_203_-	GH92		alpha-1,2-mannosidases	Oligosaccharide-degrading enzymes	Glyco_hydro_92
Bacteroidetes_sca2029 size270_1_270_+	GH97		alpha- galactosidases	Oligosaccharide-degrading enzymes	Glyco_hydro_97
Bacteroidetes_sca239 size509_1_509_-	GH97		alpha- galactosidases	Oligosaccharide-degrading enzymes	Glyco_hydro_97
Bacteroidetes_sca2898 size236_1_236_+	GH97		alpha- galactosidases	Oligosaccharide-degrading enzymes	Glyco_hydro_97
Bacteroidetes_sca523 size415_1_415_-	GH97		alpha- galactosidases	Oligosaccharide-degrading enzymes	Glyco_hydro_97
Cytophagales_sca2266 size274_1_274_-	GH97		alpha- galactosidases	Oligosaccharide-degrading enzymes	Glyco_hydro_97
Cytophagales_sca2619 size262_1_262_-	GH97		alpha- galactosidases	Oligosaccharide-degrading enzymes	Glyco_hydro_97
Cytophagales_sca3242 size241_1_241_+	GH97		alpha- galactosidases	Oligosaccharide-degrading enzymes	Glyco_hydro_97
Cytophagales_sca468 size444_1_444_-	GH97		alpha- galactosidases	Oligosaccharide-degrading enzymes	Glyco_hydro_97
Flavobacteriales_sca2735 size405_1_405_+	GH97		alpha- galactosidases	Oligosaccharide-degrading enzymes	Glyco_hydro_97
Flavobacteriales_sca2940 size394_1_394_-	GH97		alpha- galactosidases	Oligosaccharide-degrading enzymes	Glyco_hydro_97
Flavobacteriales_sca5437 size307_1_307_+	GH97		alpha- galactosidases	Oligosaccharide-degrading enzymes	Glyco_hydro_97
Flavobacteriales_sca6558 size284_1_284_+	GH97		alpha- galactosidases	Oligosaccharide-degrading enzymes	Glyco_hydro_97
Flavobacteriales_sca7584 size265_1_265_+	GH97		alpha- galactosidases	Oligosaccharide-degrading enzymes	Glyco_hydro_97
Flavobacteriales_sca7889 size261_1_261_+	GH97		alpha- galactosidases	Oligosaccharide-degrading enzymes	Glyco_hydro_97
Flavobacteriales_sca9742 size235_1_235_-	GH97		alpha- galactosidases	Oligosaccharide-degrading enzymes	Glyco_hydro_97
OtherBacteria_sca2539 size297_1_297_+	GH97		alpha- galactosidases	Oligosaccharide-degrading enzymes	Glyco_hydro_97
OtherBacteria_sca3275 size274_1_274_+	GH97		alpha- galactosidases	Oligosaccharide-degrading enzymes	Glyco_hydro_97
OtherBacteria_sca5042 size240_1_240_+	GH97		alpha- galactosidases	Oligosaccharide-degrading enzymes	Glyco_hydro_97
Xanthoonadales_sca1693 size237_1_237_+	GH97		alpha- galactosidases	Oligosaccharide-degrading enzymes	Glyco_hydro_97
Xanthoonadales_sca249 size433_1_433_+	GH97		alpha- galactosidases	Oligosaccharide-degrading enzymes	Glyco_hydro_97
Bacteroidetes_sca1851 size279_1_279_-	PL10		pectate lyase	pectate lyase	Pec_lyase
Enterobacteriales_sca3784 size245_1_245_+	PL10		pectate lyase	pectate lyase	Pec_lyase
OtherBacteria_sca8091 size205_1_205_+	PL10		pectate lyase	pectate lyase	Pec_lyase
Xanthoonadales_sca386 size376_130_376_+	PL10		pectate lyase	pectate lyase	Pec_lyase
Enterobacteriales_sca810 size363_1_297_-	PL2		pectate lyase	pectate lyase	Pectate_lyase_2
Pseudoonadales_contig_109482_1_208_-	PL7		Alginate lyase		Alginate_lyase2

Supplementary Table S8 Pupative genes related to Nitrogen metabolism in tomato root gall-associated microbiome

Functional category	pupative gene	root ga	Burkholde	Enterobac	Flavobact	Pseudomon	Rhizobia	Rhodocyc	Sphingoba	Sphingom	otherBacteria
Allantoin Utilization	2-hydroxy-3-oxopropionate reductase (EC	25	2	2		2	7	6		3	2
Allantoin Utilization	Allantoate amidohydrolase (EC 3.5.3.9)	3	2				1				
Allantoin Utilization	Allantoinase (EC 3.5.2.5)	2					2				
Allantoin Utilization	Glycerate kinase (EC 2.7.1.31)	7		4		1			1		2
Allantoin Utilization	Glyoxylate carboligase (EC 4.1.1.47)	4				3					
Allantoin Utilization	Ureidoglycolate dehydrogenase (EC 1.1.1	3									2
Allantoin Utilization	Ureidoglycolate hydrolase (EC 3.5.3.19)	1					1				
Ammonia assimilation	Ammonium transporter	39	4		6	3	2	6	4	2	8
Ammonia assimilation	Ammonium transporter family	3			1				2		
Ammonia assimilation	Ferredoxin-dependent glutamate synthase	28	2		8	2	1	1	8	2	2
Ammonia assimilation	Glutamate synthase [NADPH] large chain	96	4	4	2	9	10	8	32	7	10
Ammonia assimilation	Glutamate synthase [NADPH] putative Gl	2				1	1				
Ammonia assimilation	Glutamate synthase [NADPH] small chain	39	6	3		2	2	1	10	5	8
Ammonia assimilation	Glutamate-ammonia-ligase adenylyltrans	31	3	5		3	5	3		2	8
Ammonia assimilation	Glutamine amidotransferase protein GlxB	1					1				
Ammonia assimilation	Glutamine amidotransferase, class-II	3	1					1			1
Ammonia assimilation	Glutamine synthetase type I (EC 6.3.1.2	16	2	1		3	5			2	2
Ammonia assimilation	Glutamine synthetase type II, eukaryoti	1									1
Ammonia assimilation	Glutamine synthetase type III, GlnN (EC	51			17				25		2
Ammonia assimilation	Nitrogen regulation protein NR(I)	18	5	1			2	7			2
Ammonia assimilation	Nitrogen regulation protein NR(II) (EC	4				1		1			2
Ammonia assimilation	Nitrogen regulatory protein P-II	6	1	1				2			2
Ammonia assimilation	Nitrogen regulatory protein P-II, glnK	1		1							
Ammonia assimilation	[Protein-PII] uridylyltransferase (EC 2	34	5	2		1	9	5		4	8
Denitrification	Copper-containing nitrite reductase (EC	11	1		2		1		1		3
Denitrification	Cytochrome cd1 nitrite reductase (EC:1.	14	2					9			1
Denitrification	Nitric oxide -responding transcriptiona	8	1			2		5			
Denitrification	Nitric oxide -responding transcriptiona	1					1				
Denitrification	Nitric oxide reductase activation prote	7	1		1	1	2	1			1
Denitrification	Nitric oxide reductase activation prote	7	1					5			
Denitrification	Nitric oxide reductase activation prote	3			1			2			
Denitrification	Nitric-oxide reductase (EC 1.7.99.7), c	18	4		4	1		1	1		7
Denitrification	Nitric-oxide reductase subunit B (EC 1.	14	1		4	2	3	4			
Denitrification	Nitric-oxide reductase subunit C (EC 1.	3			1			2			
Denitrification	Nitrite reductase accessory protein Nir	2					2				
Denitrification	Nitrous oxide reductase maturation peri	3	1				2				
Denitrification	Nitrous oxide reductase maturation prot	17	4		2	1	1	2	5		3
Denitrification	Nitrous oxide reductase maturation prot	3						2			2
Denitrification	Nitrous oxide reductase maturation prot	9	4			3					1
Denitrification	Nitrous oxide reductase maturation prot	5	1					3			1
Denitrification	Nitrous oxide reductase maturation tran	3	1					1	1		
Denitrification	Nitrous-oxide reductase (EC 1.7.99.6)	22	5		2			6	3		7
Denitrification	NnrS protein involved in response to NO	9	1			1	1	5			1
Denitrification	NnrU family protein, required for expre	5	1				4				
Dissimilatory nitrite rec	Cytochrome c551 NirM	1						1			
Dissimilatory nitrite rec	Cytochrome c55X precursor NirC	1						1			
Dissimilatory nitrite rec	Cytochrome cd1 nitrite reductase (EC:1.	14	2					9			1
Dissimilatory nitrite rec	Heme d1 biosynthesis protein NirD	2	1					1			
Dissimilatory nitrite rec	Heme d1 biosynthesis protein NirH	2				1		1			
Dissimilatory nitrite rec	Heme d1 biosynthesis protein NirJ	8	2			1		4			
Dissimilatory nitrite rec	Heme d1 biosynthesis protein NirL	2	1					1			
Dissimilatory nitrite rec	Nitrite reductase associated c-type cyt	4				1		3			
Dissimilatory nitrite rec	Uroporphyrinogen-III methyltransferase	20	3			3	3			3	7
Nitrate and nitrite ammor	Assimilatory nitrate reductase large su	28	9	5		4	3	3		1	2
Nitrate and nitrite ammor	Cytochrome c-type protein NapC	9	1				1	3			4
Nitrate and nitrite ammor	Ferredoxin-type protein NapF (periplasm	2					1	1			
Nitrate and nitrite ammor	Ferredoxin-type protein NapG (periplasm	6		1				4			1
Nitrate and nitrite ammor	Nitrate ABC transporter, ATP-binding pr	8	3					1		2	1
Nitrate and nitrite ammor	Nitrate ABC transporter, nitrate-bindin	25	2			2	6	3		2	
Nitrate and nitrite ammor	Nitrate ABC transporter, permease prote	7	1	3		2	1	4			1
Nitrate and nitrite ammor	Nitrate reductase cytochrome c550-type	2					1	1			
Nitrate and nitrite ammor	Nitrate/nitrite response regulator prot	9	1			4	1	1	2		
Nitrate and nitrite ammor	Nitrate/nitrite sensor protein (EC 2.7.	14	4	5				3			
Nitrate and nitrite ammor	Nitrate/nitrite transporter	39	8	7	3	7	1		4		9
Nitrate and nitrite ammor	Nitrite reductase [NAD(P)H] large subun	38	3	4		9	6	3	2		6
Nitrate and nitrite ammor	Nitrite reductase [NAD(P)H] small subun	8	4	1		1	1	1			
Nitrate and nitrite ammor	Nitrite reductase probable [NAD(P)H] su	2					1		1		
Nitrate and nitrite ammor	Nitrite transporter from formate/nitrit	3	1					1			1
Nitrate and nitrite ammor	Periplasmic nitrate reductase precursor	14	1	1			2	5			2
Nitrate and nitrite ammor	Polyferredoxin NapH (periplasmic nitrat	3						3			
Nitrate and nitrite ammor	Putative thiol:disulfide oxidoreductase	1									1
Nitrate and nitrite ammor	Respiratory nitrate reductase alpha cha	29	5	7		1		1			12
Nitrate and nitrite ammor	Respiratory nitrate reductase beta chai	2									2
Nitrate and nitrite ammor	Respiratory nitrate reductase delta cha	1		1							
Nitrate and nitrite ammor	Respiratory nitrate reductase gamma cha	6	1	1		2					2
Nitrate and nitrite ammor	Response regulator NasT	14	1	3		2	2	1		2	3

Nitric oxide synthase	Manganese superoxide dismutase (EC 1.15	28			6	1			9		9
Nitric oxide synthase	putative cytochrome P450 hydroxylase	16	6			3	1			1	5
Nitrilase	Plant-induced nitrilase (EC 3.5.5.1), h	2				2					
Nitrilase	Transcriptional regulator in custer wit	2				2					
Nitrogen fixation	4Fe-4S ferredoxin, nitrogenase-associat	1						1			
Nitrogen fixation	Cysteine desulfurase (EC 2.8.1.7), NifS	1							1		
Nitrogen fixation	Homocitrate synthase (EC 2.3.3.14)	2						2			
Nitrogen fixation	NifM protein	1						1			
Nitrogen fixation	NifX-associated protein	2						1			1
Nitrogen fixation	Nitrogenase (molybdenum-iron) alpha cha	6	1					4			
Nitrogen fixation	Nitrogenase (molybdenum-iron) beta chai	3						3			
Nitrogen fixation	Nitrogenase (molybdenum-iron) reductase	3						1	1		1
Nitrogen fixation	Nitrogenase (molybdenum-iron)-specific	4			1		1	1	1		
Nitrogen fixation	Nitrogenase FeMo-cofactor carrier prote	1						1			
Nitrogen fixation	Nitrogenase FeMo-cofactor scaffold and	3						2			1
Nitrogen fixation	Nitrogenase FeMo-cofactor scaffold and	1									1
Nitrogen fixation	Nitrogenase FeMo-cofactor synthesis FeS	1									1
Nitrosative stress	Anaerobic nitric oxide reductase flavor	4		3							1
Nitrosative stress	Flavohemoprotein	7	2	2					1		2
Nitrosative stress	Functional role page for Anaerobic nitr	7	2			2					
Nitrosative stress	Hydroxylamine reductase (EC 1.7.-.-)	6		2							4
Nitrosative stress	NADH oxidoreductase hcr (EC 1.-.-.-)	1		1							
Nitrosative stress	Nitric oxide reductase FlRd-NAD(+) redu	2		2							
Nitrosative stress	Nitric oxide-dependent regulator DnrN c	1	1								
Nitrosative stress	Nitric-oxide reductase (EC 1.7.99.7), c	18	4		4	1		1		1	7
Nitrosative stress	Nitrite-sensitive transcriptional repre	5	1					1		1	2
Nitrosative stress	NnrS protein involved in response to NO	9	1			1	1	5			1

Supplementary Table S9 Pupative genes related to pathways of synthesis IAA (indole-3-acetic acid) in tomato root gall-associated microbiome

EC number	Pupative gene	Taxonomic assignment
EC:4.1.99.1	tryptophanase	Bacteroidetes
EC:4.1.99.1	tryptophanase	Bacteroidetes
EC:4.1.99.1	tryptophanase	Sphingobacteriales
EC:4.1.99.1	tryptophanase	Enterobacteriales
EC:1.13.12.3	tryptophan 2-monooxygenas	Pseudomonadales
EC:4.1.1.74	indolepyruvate decarboxylas	Enterobacteriales
EC:4.1.1.74	indolepyruvate decarboxylas	Enterobacteriales
EC:4.1.1.74	indolepyruvate decarboxylas	Enterobacteriales
EC:4.1.1.74	indolepyruvate decarboxylas	Enterobacteriales
EC:4.1.1.74	indolepyruvate decarboxylas	Enterobacteriales
EC:4.1.1.74	indolepyruvate decarboxylas	Enterobacteriales
EC:4.1.1.74	indolepyruvate decarboxylas	Enterobacteriales
EC:4.1.1.74	indolepyruvate decarboxylas	Enterobacteriales
EC:1.2.1.3	aldehyde dehydrogenase	Burkholderiales
EC:1.2.1.3	aldehyde dehydrogenase	actinobacteria
EC:1.2.1.3	aldehyde dehydrogenase	Pseudomonadales
EC:1.2.1.3	aldehyde dehydrogenase	Rhizobiales
EC:1.2.1.3	aldehyde dehydrogenase	Rhizobiales
EC:1.2.1.3	aldehyde dehydrogenase	Flavobacteriales
EC:1.2.1.3	aldehyde dehydrogenase	Rhizobiales
EC:1.2.1.3	aldehyde dehydrogenase	Sphingobacteriales
EC:1.2.1.3	aldehyde dehydrogenase	Burkholderiales
EC:1.2.1.3	aldehyde dehydrogenase	Rhizobiales
EC:1.2.1.3	aldehyde dehydrogenase	Rhizobiales
EC:1.2.1.3	aldehyde dehydrogenase	Rhizobiales
EC:1.2.1.3	aldehyde dehydrogenase	Rhizobiales
EC:1.2.1.3	aldehyde dehydrogenase	Rhizobiales
EC:1.2.1.3	aldehyde dehydrogenase	otherBacteria
EC:1.2.1.3	aldehyde dehydrogenase	Rhizobiales
EC:1.2.1.3	aldehyde dehydrogenase	Sphingomonadales
EC:1.2.1.3	aldehyde dehydrogenase	Pseudomonadales
EC:1.2.1.3	aldehyde dehydrogenase	Rhizobiales
EC:1.2.1.3	aldehyde dehydrogenase	Flavobacteriales
EC:1.2.1.3	aldehyde dehydrogenase	Flavobacteriales
EC:1.2.1.3	aldehyde dehydrogenase	Rhizobiales
EC:1.2.1.3	aldehyde dehydrogenase	Sphingobacteriales
EC:1.2.1.3	aldehyde dehydrogenase	Caulobacterales
EC:1.2.1.3	aldehyde dehydrogenase	Burkholderiales
EC:1.2.1.3	aldehyde dehydrogenase	Pseudomonadales
EC:1.2.1.3	aldehyde dehydrogenase	Pseudomonadales
EC:1.2.1.3	aldehyde dehydrogenase	otherBacteria
EC:1.2.1.3	aldehyde dehydrogenase	Rhodocyclales
EC:1.2.1.3	aldehyde dehydrogenase	Flavobacteriales
EC:1.2.1.3	aldehyde dehydrogenase	Burkholderiales
EC:1.2.1.3	aldehyde dehydrogenase	Rhizobiales
EC:1.2.1.3	aldehyde dehydrogenase	Pseudomonadales
EC:1.2.1.3	aldehyde dehydrogenase	Flavobacteriales
EC:1.2.1.3	aldehyde dehydrogenase	Flavobacteriales
EC:1.2.1.3	aldehyde dehydrogenase	Flavobacteriales
EC:1.2.1.3	aldehyde dehydrogenase	Flavobacteriales
EC:1.2.1.3	aldehyde dehydrogenase	Sphingobacteriales
EC:1.2.1.3	aldehyde dehydrogenase	Rhodocyclales
EC:1.2.1.3	aldehyde dehydrogenase	Xanthomonadales
EC:1.2.1.3	aldehyde dehydrogenase	Rhodocyclales
EC:1.2.1.3	aldehyde dehydrogenase	Cytophagales
EC:1.2.1.3	aldehyde dehydrogenase	Sphingobacteriales
EC:1.2.1.3	aldehyde dehydrogenase	Sphingobacteriales
EC:1.2.1.3	aldehyde dehydrogenase	Sphingobacteriales
EC:1.2.1.3	aldehyde dehydrogenase	Burkholderiales
EC:1.2.1.3	aldehyde dehydrogenase	Sphingobacteriales
EC:1.2.1.3	aldehyde dehydrogenase	Cytophagales
EC:1.2.1.3	aldehyde dehydrogenase	Flavobacteriales
EC:1.2.1.3	aldehyde dehydrogenase	otherBacteria
EC:1.2.1.3	aldehyde dehydrogenase	Sphingobacteriales
EC:1.2.1.3	aldehyde dehydrogenase	Sphingobacteriales
EC:1.2.1.3	aldehyde dehydrogenase	otherBacteria
EC:1.2.1.3	aldehyde dehydrogenase	Sphingobacteriales
EC:1.2.1.3	aldehyde dehydrogenase	Rhizobiales
EC:1.2.1.3	aldehyde dehydrogenase	Flavobacteriales
EC:1.2.1.3	aldehyde dehydrogenase	Flavobacteriales
EC:1.2.1.3	aldehyde dehydrogenase	Flavobacteriales

EC:1.2.1.3	aldehyde dehydrogenase	Flavobacteriales
EC:1.2.1.3	aldehyde dehydrogenase	Flavobacteriales
EC:1.2.1.3	aldehyde dehydrogenase	Burkholderiales
EC:1.2.1.3	aldehyde dehydrogenase	Burkholderiales
EC:1.2.1.3	aldehyde dehydrogenase	Burkholderiales
EC:1.2.1.3	aldehyde dehydrogenase	Flavobacteriales
EC:1.2.1.3	aldehyde dehydrogenase	Sphingobacteriales
EC:1.2.1.3	aldehyde dehydrogenase	Xanthomonadales
EC:1.2.1.3	aldehyde dehydrogenase	Flavobacteriales
EC:1.2.1.3	aldehyde dehydrogenase	Sphingobacteriales
EC:1.2.1.3	aldehyde dehydrogenase	Sphingobacteriales
EC:1.2.1.3	aldehyde dehydrogenase	Firmicutes
EC:1.2.1.3	aldehyde dehydrogenase	otherBacteria
EC:1.2.1.3	aldehyde dehydrogenase	Caulobacterales
EC:1.2.1.3	aldehyde dehydrogenase	Rhizobiales
EC:1.2.1.3	aldehyde dehydrogenase	Rhizobiales
EC:1.2.1.3	aldehyde dehydrogenase	Burkholderiales
EC:1.2.1.3	aldehyde dehydrogenase	Burkholderiales
EC:1.2.1.3	aldehyde dehydrogenase	Burkholderiales
EC:1.2.1.3	aldehyde dehydrogenase	Flavobacteriales
EC:1.2.1.3	aldehyde dehydrogenase	Enterobacteriales
EC:1.2.1.3	aldehyde dehydrogenase	otherBacteria
EC:1.2.1.3	aldehyde dehydrogenase	Flavobacteriales
EC:1.2.1.3	aldehyde dehydrogenase	otherBacteria
EC:1.2.1.3	aldehyde dehydrogenase	Sphingobacteriales
EC:1.2.1.3	aldehyde dehydrogenase	Burkholderiales
EC:1.2.1.3	aldehyde dehydrogenase	Burkholderiales
EC:1.2.1.3	aldehyde dehydrogenase	Burkholderiales
EC:1.2.1.3	aldehyde dehydrogenase	Rhizobiales
EC:1.2.1.3	aldehyde dehydrogenase	Caulobacterales
EC:1.2.1.3	aldehyde dehydrogenase	Burkholderiales
EC:1.2.1.3	aldehyde dehydrogenase	otherBacteria
EC:1.2.1.3	aldehyde dehydrogenase	Burkholderiales
EC:1.2.1.3	aldehyde dehydrogenase	Burkholderiales
EC:1.2.1.3	aldehyde dehydrogenase	Burkholderiales
EC:1.2.1.3	aldehyde dehydrogenase	Burkholderiales
EC:1.2.1.3	aldehyde dehydrogenase	Burkholderiales
EC:1.2.1.3	aldehyde dehydrogenase	Cytophagales
EC:3.5.5.1	nitrilase	Rhizobiales
EC:3.5.5.1	nitrilase	Burkholderiales
EC:3.5.5.1	nitrilase	Pseudomonadales
EC:3.5.5.1	nitrilase	Pseudomonadales
EC:3.5.5.1	nitrilase	Burkholderiales
EC:3.5.5.1	nitrilase	Burkholderiales
EC:3.5.5.1	nitrilase	Rhodocyclales
EC:3.5.5.1	nitrilase	otherBacteria
EC:3.5.5.1	nitrilase	Rhodocyclales
EC:3.5.1.4	amidase	Burkholderiales
EC:3.5.1.4	amidase	Xanthomonadales
EC:3.5.1.4	amidase	Pseudomonadales
EC:3.5.1.4	amidase	Burkholderiales
EC:3.5.1.4	amidase	Rhizobiales
EC:3.5.1.4	amidase	Burkholderiales
EC:3.5.1.4	amidase	Pseudomonadales
EC:3.5.1.4	amidase	Burkholderiales
EC:3.5.1.4	amidase	Caulobacterales
EC:3.5.1.4	amidase	Pseudomonadales
EC:3.5.1.4	amidase	Xanthomonadales
EC:3.5.1.4	amidase	Burkholderiales
EC:3.5.1.4	amidase	Burkholderiales
EC:3.5.1.4	amidase	Sphingomonadales
EC:1.4.3.4	monoamine oxidase	Rhizobiales
EC:1.4.3.4	monoamine oxidase	Pseudomonadales
EC:1.4.3.4	monoamine oxidase	Bdellovibrionales
EC:1.4.3.4	monoamine oxidase	Bdellovibrionales
EC:1.4.3.4	monoamine oxidase	Rhizobiales
EC:1.4.3.4	monoamine oxidase	Caulobacterales
EC:1.11.1.6	catalase	Enterobacteriales
EC:1.11.1.6	catalase	Enterobacteriales
EC:1.11.1.6	catalase	Enterobacteriales
EC:1.4.3.4	monoamine oxidase	Cytophagales
EC:1.4.3.4	monoamine oxidase	Enterobacteriales
EC:1.4.3.4	monoamine oxidase	otherBacteria

Supplementary Table S10 Pupative genes related to biosynthesis of siderophore group nonribosomal peptides in tomato root gall-associated microbiome

Taxonomic assignment	Assembly	Pupative gene [EC number]
Burkholderiales	contig_239354_92_253	isochorismate pyruvate-lyase [EC:4.1.3.-]
	contig_345734_1_204	isochorismate pyruvate-lyase [EC:4.1.3.-]
	contig_622251_1_209	nonribosomal peptide synthetase DhbF
Caulobacterales	contig_102366_1_203	enterobactin synthetase component F [EC:2.7.7.-]
	contig_213289_1_224	enterobactin synthetase component F [EC:2.7.7.-]
	contig_311017_1_476	2,3-dihydro-2,3-dihydroxybenzoate dehydrogenase
	contig_635470_1_227	mycobactin salicyl-AMP ligase [EC:6.3.2.-]
Cytophagales	contig_375107_1_287	nonribosomal peptide synthetase DhbF
Enterobacteriales	contig_289054_1_252	enterobactin synthetase component F [EC:2.7.7.-]
	contig_289055_1_264	enterobactin synthetase component F [EC:2.7.7.-]
	contig_314147_1_301	enterobactin synthetase component F [EC:2.7.7.-]
	contig_314147_1_301	enterobactin synthetase component F [EC:2.7.7.-]
	contig_369477_1_214	enterobactin synthetase component F [EC:2.7.7.-]
	contig_373316_1_78	enterobactin synthase subunit E (EC:1.2.3.5)
	contig_387443_1_209	enterobactin synthetase component F [EC:2.7.7.-]
	contig_41286_1_204	enterobactin synthetase component F [EC:2.7.7.-]
	contig_439872_1_219	enterobactin synthetase component F [EC:2.7.7.-]
	contig_460421_1_210	enterobactin synthetase component F [EC:2.7.7.-]
	contig_727668_1_216	enterobactin synthase subunit E (EC:1.2.3.5)
otherBacteria	contig_123318_1_251	enterobactin 2,3-dihydroxybenzoate-AMP ligase/S-dihydroxybenzoyltransferase
	contig_637320_1_251	yersiniabactin nonribosomal peptide/polyketide synthase
Pseudomonadales	contig_131541_1_448	dihydroaeruginoic acid synthetase
	contig_216595_1_217	pyochelin synthetase
	contig_216596_1_365	pyochelin synthetase
	contig_243928_1_336	pyochelin biosynthetic protein PchG
	contig_354252_1_219	dihydroaeruginoic acid synthetase
	contig_412229_1_274	pyochelin biosynthesis protein PchD
	contig_4409_147_315	pyochelin synthetase
	contig_461131_1_267	pyochelin synthetase
	contig_56304_1_277	pyochelin biosynthesis protein PchD
Rhizobiales	contig_190480_286_400	enterobactin synthetase component F [EC:2.7.7.-]
	contig_350985_1_207	enterobactin synthase subunit E
	contig_415084_1_288	nonribosomal peptide synthetase VibF
	contig_450153_1_217	enterobactin isochorismatase [EC:3.3.2.1]
	contig_474769_1_235	enterobactin synthase subunit E (EC:2.7.7.58)
Rhodocyclales	contig_100011_1_294	isochorismate pyruvate-lyase [EC:4.1.3.-]
Sphingomonadales	contig_103899_380_500	isochorismate pyruvate-lyase [EC:4.1.3.-]
Xanthomonadales	contig_215151_192_258	enterobactin synthetase component F [EC:2.7.7.-]

Supplementary Table S11 Subsystem functional categories significantly enriched in major bacterial groups of root gall-associated metagenome

Bacterial group	Functional category	p-value	Functional contribution
Sphingobacteriales	Carbohydrates	0.0409	
	Glycoside hydrolases	0.0048	enrich
	Central carbohydrate r	0.0477	enrich
	Protein metabolism		
	protein biosynthesis	0.036	enrich
	Secondary metabolism		
	plant alkaloids	0.0064	enrich
	Fatty acids, and Isoprenoids		
	Isoprenoids	0.0443	enrich
	Cofactors, vitamins, prosthetic groups, pigments		
	Lipoic acid	0.0243	enrich
	Clustering-based subsystems		
	Oxidative stress	0.0082	
	probably GTP and GMI	0.0321	
	Pyruvate kinase associ	0.0436	
	RNA Metabolism		
	transcription	0.0403	
Rhodocyclales	Nitrogen Metabolism	0.0137	enrich
	Carbohydrates		
	Di- and oligosaccharide	0.0053	decrease
	polysaccharides	0.0223	decrease
	Membrane transpot	0.0047	decrease
	protein secretion syste	0.0353	decrease
	Iron acquisition and meta	0.026	decrease
Rhizobiales	Regulation and Cell signa	0.0028	enrich
	Oxidative stress	0.0217	enrich
Flavobacteriales	Regulation and Cell signa	0.0234	enrich
	Sulfur Metabolism	0.035	decrease
Burkholderiales	Stress Response	0.0493	enrich
	Oxidative stress	0.0226	
	Membrane transpot	0.0047	enrich
	Sulfur Metabolism	0.0404	enrich
Pseudomonadales	Secondary metabolism		
	biosynthesis of phenyl	0.0451	enrich
Enterobacteriales	Stress Response	0.0493	
	Dessication stress	0	enrich
	Detoxification	0.05	enrich
	Regulation and Cell signa	0.05	
	Quorum sensing and b	0.0051	enrich
